# Enhancing carbon nanotubes production from pyrolysis–catalysis of plastic waste through monolithic heating

**DOI:** 10.1093/nsr/nwag143

**Published:** 2026-03-09

**Authors:** Ruming Pan, Jie Yu, Youwei Yang, Ilman Nuran Zaini, Xibo He, Muhammad Rafique, Yibo Wu, Kuandong Jiang, Leilei Dai, Yi Fang, Youchuang Chao, Yuming Wen, Yanming Guo, Gérald Debenest, Weihong Yang, Wangliang Li, Yaning Zhang, Yong Shuai

**Affiliations:** School of Energy Science and Engineering, Harbin Institute of Technology, Harbin 150001, China; School of Energy Science and Engineering, Harbin Institute of Technology, Harbin 150001, China; School of Energy Science and Engineering, Harbin Institute of Technology, Harbin 150001, China; Faculty of Mechanical and Aerospace Engineering, Institut Teknologi Bandung, Bandung 40132, Indonesia; School of Energy Science and Engineering, Harbin Institute of Technology, Harbin 150001, China; School of Energy Science and Engineering, Harbin Institute of Technology, Harbin 150001, China; Chongqing Research Institute of Harbin Institute of Technology, Chongqing 400020, China; School of Energy Science and Engineering, Harbin Institute of Technology, Harbin 150001, China; Institut de Mécanique des Fluides de Toulouse (IMFT)-Université de Toulouse, CNRS-INPT-UPS, Toulouse 31400, France; Center for Biorefining, and Department of Bioproducts and Biosystems Engineering, University of Minnesota Twin Cities, St. Paul, MN 55108, USA; CAS Key Laboratory of Green Process and Engineering, Institute of Process Engineering, Chinese Academy of Sciences, Beijing 100190, China; University of Chinese Academy of Sciences, Beijing 100049, China; School of Energy Science and Engineering, Harbin Institute of Technology, Harbin 150001, China; Institute of Sustainability for Chemicals, Energy and Environment (ISCE2), Agency for Science, Technology and Research (A*STAR), Singapore 627833, Singapore; Department of Chemical and Biomolecular Engineering, National University of Singapore, Singapore 117585, Singapore; School of Energy Science and Engineering, Harbin Institute of Technology, Harbin 150001, China; Institut de Mécanique des Fluides de Toulouse (IMFT)-Université de Toulouse, CNRS-INPT-UPS, Toulouse 31400, France; Department of Materials Science and Engineering, KTH Royal Institute of Technology, Stockholm 114 28, Sweden; CAS Key Laboratory of Green Process and Engineering, Institute of Process Engineering, Chinese Academy of Sciences, Beijing 100190, China; University of Chinese Academy of Sciences, Beijing 100049, China; School of Energy Science and Engineering, Harbin Institute of Technology, Harbin 150001, China; School of Energy Science and Engineering, Harbin Institute of Technology, Harbin 150001, China

**Keywords:** plastic waste, carbon nanotubes, monolithic heating, porous catalysts, pyrolysis–catalysis

## Abstract

In this study, we developed a novel monolithic heating method by utilizing electromagnetic induction to enhance carbon nanotube (CNT) production from plastic waste via pyrolysis–catalysis. Metal porous catalysts, including iron (Fe), nickel (Ni) and Fe–Ni alloy, enabled rapid volumetric heating, improved catalytic efficiency and reduced energy consumption compared with conventional methods. The porous Fe catalyst demonstrated superior CNT yield, while Ni provided the highest hydrogen (H_2_) production. We achieved carbon-recovery efficiencies of 86%, 84% and 82% for low-density polyethylene, high-density polyethylene and polypropylene, respectively, by using the Fe catalyst. Our economic analysis indicated that CNT production from waste polyethylene and waste polypropylene is feasible, with break-even selling prices of $4.02 and $4.87 kg^−1^, respectively. Our work supports the development of a circular economy by enabling the efficient conversion of plastic waste into valuable CNTs and H_2_ via pyrolysis–catalysis.

## Teaser

Electromagnetic induction–heated metal porous catalysts enable efficient conversion of plastic waste into high-value carbon nanotubes and hydrogen, demonstrating promising economic feasibility for circular waste utilization.

## INTRODUCTION

Plastics—particularly polyolefins such as polyethylene (PE) and polypropylene (PP)—are major contributors to global waste accumulation, with billions of tons having been either landfilled or discharged into the environment [1,2]. Due to their persistent nature and harmful effects on ecosystems, human health and the economy, plastic pollution has been identified as a critical global issue requiring immediate action [3]. In response, recycling technologies have received increasing attention and they are generally categorized into physical recycling, chemical recycling and energy-recovery methods [4,5]. Physical recycling involves the mechanical processing of plastic waste through cleaning, shredding and re-melting. While economically viable and technologically mature, physical recycling requires high material purity and often results in reduced performance after multiple cycles [6]. Energy recovery incinerates plastic waste to generate heat but produces harmful substances, such as dioxins, raising environmental and health concerns [7]. Chemical recycling converts plastic polymers into monomers or low-molecular-weight compounds, enabling the recovery from mixed or contaminated waste streams [8,9]. Catalytic pyrolysis of waste plastics at low to medium temperatures has been widely investigated as a chemical recycling strategy. Operating typically at 300–650°C, this pathway targets the selective production of light olefins and aromatic hydrocarbons through catalytic cracking and aromatization [10,11]. Zeolite-based catalysts—especially HZSM-5 and its derivatives—play a key role in regulating product selectivity via acidity and pore-diffusion

effects [12,13]. In contrast, high-temperature catalytic reforming (>700°C) promotes deep dehydrogenation and carbon restructuring, enabling the co-production of hydrogen (H_2_) and solid carbon materials such as carbon nanotubes (CNTs) [14]. In this process, plastics are first thermally decomposed into hydrocarbon vapors, which are subsequently transformed into CNTs and H_2_ through catalytic reactions [15]. CNT formation generally proceeds via the decomposition of hydrocarbon precursors into atomic carbon, followed by carbon diffusion and saturation within metal catalyst particles and subsequent precipitation as ordered tubular carbon structures [16,17]. H_2_ produced through this route provides a more sustainable option compared with fossil-fuel-based methods, offering lower greenhouse gas emissions and reduced dependence on natural gas [18–20]. Meanwhile, CNTs—valued for their excellent mechanical, electrical and thermal properties—support advanced applications in electronics, composite materials and energy-storage systems [21–23]. By transforming plastic waste into functional materials, this approach contributes to the circular economy and reduces dependency on fossil-based resources.

Catalysts are essential for enhancing the selectivity of catalytic reforming toward desirable products, such as CNTs and H_2_, primarily by lowering the reaction activation energy [24]. Nickel (Ni)-based catalysts have been widely investigated for H_2_ production due to their high activity and availability. Their performance can be improved through the incorporation of promoters, such as ruthenium and cerium, which enhance H_2_ yield and mitigate surface-encapsulating amorphous coke deposition [25,26]. Iron (Fe)-based catalysts also demonstrate good durability, particularly in cost-sensitive applications, as Fe exhibits greater resistance to sintering under high-temperature conditions compared with Ni. However, deactivation due to carbon (C) accumulation remains a concern. This issue can be mitigated by incorporating manganese, which promotes C gasification and improves catalyst stability [27]. Bimetallic catalysts, such as Ni–Fe, have been utilized to exploit the synergistic effects of both metals, resulting in improved catalytic activity. These catalysts have shown strong potential for CNT formation by facilitating C solubility, enhancing diffusion and suppressing coke formation [18,28]. Zeolite-supported systems, including Ni/ZSM-5, offer advantages, such as enhanced thermal stability and sintering resistance. The porous structure of zeolites enables better metal dispersion, which reduces catalyst agglomeration and prolongs activity [19]. Nonetheless, coke deposition remains an unresolved issue, albeit less severe than with unsupported Ni catalysts [29]. Conventional granular catalysts are susceptible to sintering and agglomeration at elevated temperatures. Furthermore, the CNTs synthesized on these particles are challenging to separate mechanically. Recently, mesh-type stainless-steel catalysts have demonstrated the ability to efficiently convert pyrolysis vapors into high yields of H_2_ and CNTs [14]. The CNTs were subsequently separated from the catalyst surface by using ultrasound without destroying the structure. However, this method still required acid washing and ablation, and its catalytic efficiency declined markedly after five cycles. Additionally, limitations in heat and mass transfer during catalytic reforming have led to nonuniform heating and incomplete conversion of volatiles. These challenges have remained inadequately addressed in previous studies, particularly concerning the energy demands of catalytic reforming. The reactor design also contributes to inefficiencies, as traditional systems often fail to provide the necessary temperature control, heat uniformity and mass transport required for optimizing CNT yield and quality.

**Figure 1.**
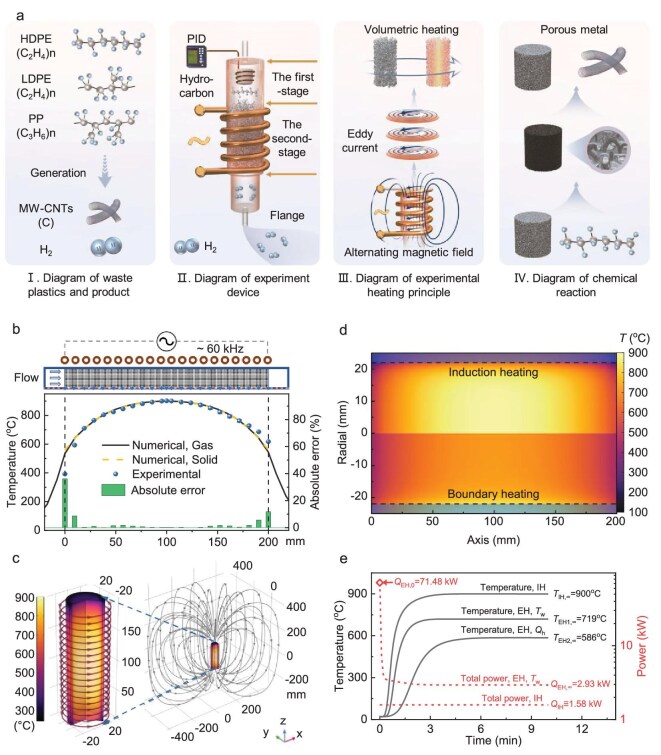
Comparison between electromagnetic induction heating and traditional electric heating. (a) Schematic of CNT production from pyrolysis–catalysis of plastic waste with electromagnetic induction monolith heating. (b) Comparison of experimental and numerical simulated temperatures. (c) Magnetic field and metal porous media temperature distributions during electromagnetic induction heating. (d) Comparison of the temperature distributions of induction heating and conventional electric heating. (e) Comparison of temperature and power-consumption changes during electromagnetic induction heating and conventional electric heating. The subscripts 0 and ∞ represent the initial and final states; *T*_w_ and *Q*_h_ represent the conventional electric heating method that maintains the same constant wall temperature and the same power as electromagnetic induction heating.

This study developed a monolithic heated metal porous catalyst for the pyrolysis–catalytic conversion of waste plastics into CNTs and H_2_. Because waste plastics recovered from municipal solid waste streams typically contain impurities such as dirt and metals [30,31], we specifically investigated and compared the pyrolysis-reforming behavior of virgin and waste polyolefin plastics. Rapid volumetric heating of the metal porous structure was achieved through electromagnetic induction (Fig. 1a), consuming approximately half the power required by conventional electric heating. We evaluated the catalytic performance of Ni, Fe and Fe–Ni porous catalysts. While the Ni-based catalyst yielded the highest H_2_ output, the Fe-based catalyst exhibited superior performance for CNT formation. The difference was attributed to the physical adsorption of dissociated C atoms by Ni and chemical adsorption by Fe. Record-high C-recovery efficiencies, indicated by the CNT yield, were achieved across various polyolefin plastics, with no significant decline in catalytic performance after 10 reaction cycles. The CNTs were effectively separated from the catalyst through mechanical vibration and no notable changes were observed in catalyst properties before and after use. A techno-economic assessment confirmed the economic viability of CNT and H_2_ production from waste PE (WPE) and waste PP (WPP), highlighting significant cost advantages. The results demonstrate the feasibility of efficient plastic-waste valorization through the integration of a low-cost, durable Fe porous catalyst and electromagnetic induction heating.

## RESULTS AND DISCUSSION

### Electromagnetic induction heating for rapid monolithic temperature rise

Figure 1a and Figs S1 and S2 show the experimental setup for CNT production via the pyrolysis–catalysis of plastic waste by using electromagnetic induction heating. In the first-stage pyrolysis, plastics are thermally decomposed to generate hydrocarbon volatiles, which are subsequently reformed in the second-stage catalysis to produce H_2_ and C. At appropriate temperatures, C can be deposited onto metal catalysts and converted into CNTs [32]. However, the accumulation of CNTs on the catalyst surface over time reduces the active contact area, hindering catalytic performance and necessitating periodic regeneration [14]. To address the limitations of fixed-bed reactors in achieving rapid heating, we designed and fabricated metal porous media composed of pure Ni, pure Fe and Fe–Ni alloy as catalysts for the reforming of hydrocarbon volatiles. The porous metal catalysts used in this study exhibit negligible micro-/mesoporosity (Fig. S3) and have pore-per-inch (PPI) values of 10, 20 and 30, corresponding to average macropore diameters of 2.54, 1.27 and 0.85 mm, respectively. The porous metal exhibited no detectable sintering or structural deformation during the reaction. Electromagnetic induction heating was applied to enable rapid and uniform temperature elevation across the monolithic structure. The use of porous media can enhance catalytic efficiency due to its high specific surface area and minimize pressure drops in the reactor in comparison with conventional packed-bed systems [33].

We developed an equivalent porous medium model and the relevant governing equations to simulate the electromagnetic induction heating of metal porous media (Equations S41–S43); these were then implemented in COMSOL software for computational fluid dynamics modeling (Figs S4 and S5). A comparison between the experimental data and equivalent porous medium model predictions (Fig. 1b) demonstrated their good agreement, with deviations within 2% except at the inlet and outlet regions, where increased convective and radiative losses likely caused lower observed temperatures. A radial temperature gradient of ∼250°C was recorded, which could be attributed to the nonuniform distribution of the eddy-current density. Nevertheless, the porous media exhibited acceptable radial temperature uniformity, even in the absence of thermal insulation (Fig. 1c).

Figure 1d presents a comparison of the temperature distributions in tubular reactors filled with porous media under conventional electric heating (boundary heating) and electromagnetic induction heating. Electromagnetic induction heating significantly improved the radial temperature uniformity. When conventional electric heating was adjusted to match the boundary temperature of the induction heating, the required power was twice that for the induction heating, while the center temperature was 181°C lower, reaching only 719°C (Fig. 1e). Under equal power conditions, conventional heating produced a center temperature that was >300°C lower, resulting in a temperature of 586°C, and required twice the time to reach steady state. These results indicate that electromagnetic induction heating enables a rapid and uniform temperature rise, making it highly suitable for the pyrolysis–catalysis of plastic waste.

### Boosting CNT production from the pyrolysis–catalysis process by monolithically heating the metal porous catalyst

**Figure 2.**
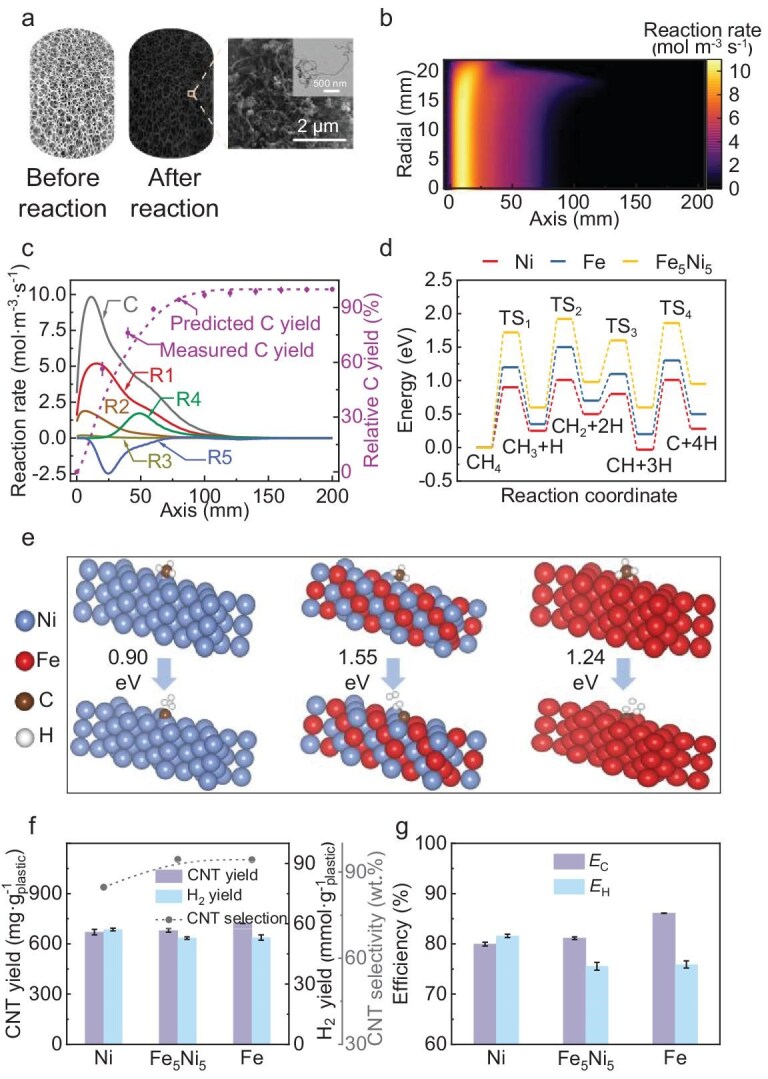
Conversion mechanism and yields of waste plastics into CNTs and H_2_ using porous media catalysts. (a) Appearance changes of the Fe metal porous media in plastic pyrolysis–catalysis. (b) C-deposition rate on the Fe metal porous media calculated by using computational fluid dynamics (CFD) simulation. (c) Reaction rates for plastic catalytic reforming sub-reactions and comparison of predicted and experimental C yields. (d) DFT calculation for methane dissociation over Ni, Fe and Fe_5_Ni_5_ porous media catalysts. (e) Configurations of methane dissociation over Ni, Fe and Fe_5_Ni_5_ porous media catalysts. (f) CNT selectivity and CNT and H_2_ yields using Ni, Fe and Fe_5_Ni_5_ porous media catalysts. (g) *E*_C_ and *E*_H_ using Ni, Fe and Fe_5_Ni_5_ porous media catalysts. TS represents the transition state.

The Fe metal porous media exhibited visible darkening after the catalytic reforming of low-density polyethylene (LDPE), indicating C deposition (Fig. 2a and Fig. S6). A significant fluid temperature (*T*_f_) drop of ≤200°C was recorded at the porous media inlet (Fig. S7). Temperature sensitivity analysis revealed that this drop was primarily attributed to the change in the thermophysical properties of the gas mixture, specifically its thermal conductivity and specific heat capacity, whereas the effect of reaction enthalpy on the temperature distribution was minimal. The reacting gas mixture in the reforming zone exhibited significantly higher effective thermal conductivity and specific heat capacity compared with the pure carrier gas. As a result, when the reacting gas contacted the hot metal porous surface, a larger amount of thermal energy was absorbed to raise the internal energy of the gas, leading to a transient reduction in the local temperature. These findings indicate that temperature reduction during reforming is unavoidable, facilitating C deposition at lower temperatures. As the temperature subsequently increased, amorphous C was progressively converted into CNTs [22]. The H_2_ fraction stabilized at 84 vol%, with 16 vol% of methane (CH_4_) remaining, suggesting that the gas-phase reaction had reached equilibrium and could not completely decompose CH_4_. This observation aligns with prior results [14], in which >10 vol% of CH_4_ persisted even at 900°C.

Figure 2b presents the C-deposition rate on Fe metal porous media, with deposition primarily occurring within the region of 0–100 mm and a peak rate of 9.84 mol·m^−3^·s^−1^ observed at *z* = 11 mm. The catalytic reforming of plastic pyrolysis volatiles was characterized by five sub-reactions, R1–R5 (Equations S28–S32). The predicted C yields from the proposed reaction model aligned well with the experimental results, confirming the accuracy of the model (Fig. 2c). It was observed that pyrolysis volatiles consumed part of the self-generated H_2_ (R1) to form ethane (C_2_H_6_, R2) and methane (CH_4_, R3), thereby reducing the C yield. The experimental data also indicated that excessive temperatures decreased C deposition [14], as higher temperatures promoted reactions R2 and R3. Consequently, C accumulation was reduced in the high-temperature zones of the metal porous media (Fig. 2b).

We investigated the effects of the reforming temperature (700–900°C, Fig. S8) and porous media pore size (10–30 PPI, Fig. S9) on the pyrolysis–catalysis of LDPE. An increase in temperature led to a rise of 11.19 wt% in the solid yield, attributed to enhanced cracking of gaseous hydrocarbons. Hydrocarbons with two (C_2_ products) or more C atoms were fully decomposed through reactions R1–R4 and the CH_4_ content gradually declined at >800°C. The proportion of CNTs increased to 850°C but decreased by ∼2 wt% at 900°C due to severe metal crystalline sintering, which enlarged the metal nanoparticle sizes [34]. The amorphous C was more likely to encapsulate larger metal nanoparticles, thereby suppressing CNT formation [35]. Despite this, the highest atom-recovery efficiencies for carbon (*E*_C_) and H_2_ (*E*_H_) were achieved at 900°C, resulting from the maximum solid yield and substantial CNT selectivity. Additionally, the pore size of the metal porous media influenced the specific surface area at constant porosity [36]. Smaller pore sizes (larger PPI) provided greater surface contact with pyrolysis volatiles and prolonged the residence time in the reaction zone, increasing the solid yield while decreasing the gas yield. H_2_ selectivity increased by 4.34 vol% as the pore size decreased within the tested range. Accordingly, the highest *E*_C_ and *E*_H_ values were obtained at a pore size of 30 PPI.

Ni and Fe are the most-commonly used metals for the catalytic reforming of plastic waste. In light of this, we evaluated the catalytic performance of three porous media: pure Ni, pure Fe and Fe_5_Ni_5_ alloy. The experimental results demonstrated that the pure metals enhanced CH_4_ cracking (CH_4_ → 2H_2_ + C), resulting in higher C yields than the alloy (Fig. S10). Experimental results obtained at different temperatures (Fig. S8) indicated that CH_4_ conversion played a crucial role in determining both the carbon-recovery efficiency and the hydrogen-recovery efficiency. Therefore, despite the compositional complexity of pyrolysis volatiles (Table S12), CH_4_ was selected as a representative reactant to evaluate and compare the catalytic activity of different catalysts. We then carried out density functional theory (DFT) calculations (Figs S11–S13), which indicated that the energy barriers for CH_4_ dissociation followed the order Ni < Fe < Fe_5_Ni_5_, with values of 0.90, 1.24 and 1.55 eV, respectively (Fig. 2d and e). Consequently, Ni porous media achieved the highest C production (79.12 wt%) and H_2_ selectivity (91.41 vol%), followed by Fe (76.57 wt% and 87.51 vol%) and Fe_5_Ni_5_ (72.04 wt% and 83.49 vol%). The highest H_2_ yield and H atom-recovery efficiency (*E*_H_) were also observed for Ni (57.12 mmol·g^−1^ plastic and 81.60%), with lower values for Fe (53.12 mmol·g^−1^ and 75.89%) and Fe_5_Ni_5_ (52.88 mmol·g^−1^ and 75.54%) (Fig. 2f and g). The dissociated C atoms formed chemical bonds with Fe (chemical adsorption), whereas physical adsorption occurred on the Ni surfaces (Fig. S12). However, Ni catalysts resulted in the lowest CNT selectivity (84.71 wt%), attributed to the absence of Fe_3_C, which is considered a key intermediate in the transformation of amorphous C to CNTs [37]. CNTs produced using Ni catalysts were of lower quality, with reduced crystallinity compared with those produced by using Fe-based catalysts [38,39]. Based on both the C yield and the CNT selectivity, the CNT yields were 721.93, 680.15 and 670.18 mg·g^−1^ plastic for the Fe, Fe_5_Ni_5_ and Ni catalysts, respectively. The highest C atom-recovery efficiency (*E*_C_) was also achieved with Fe (86.11%), followed by Fe_5_Ni_5_ (81.13%) and Ni (79.94%).

The plastic-to-catalyst mass ratio is a key parameter that determines the effective utilization of catalytic active sites and ultimately controls the carbon and hydrogen efficiencies [40]. As the plastic-to-catalyst mass ratio was increased from 1:0.5 to 1:4, the *E*_C_ increased from 66.97% to 85.63% and the *E*_H_ increased from 61.64% to 72.96% (Fig. S14), indicating the enhanced availability of active catalytic sites at higher catalyst loadings. When the plastic-to-catalyst mass ratio was increased from 1:4 to 1:40, the *E*_C_ increased only marginally, from 85.63% to 86.12%. This suggests that, at a ratio of 1:4, the catalyst already provides enough active sites to effectively promote the catalytic reforming process and increasing the catalyst loading further does not lead to a meaningful improvement in the carbon efficiency.

**Figure 3.**
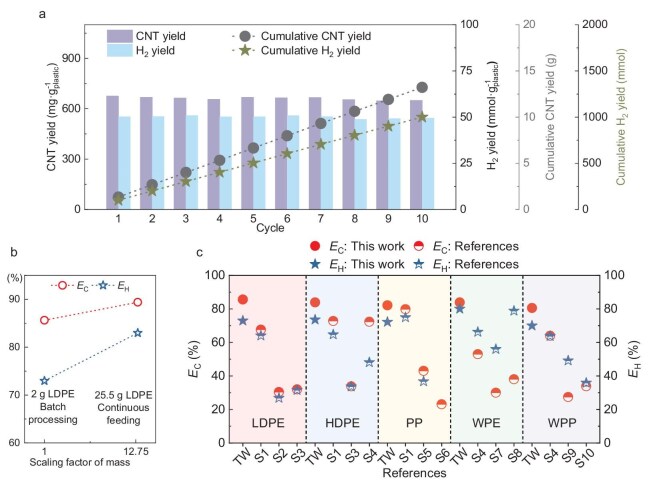
Recyclability and performance comparison of Fe porous catalysts. (a) Fe porous media catalyst cycling performance considering CNT yield, H_2_ yield, cumulative CNT yield and cumulative H_2_ yield changes. (b) *E*_C_ and *E*_H_ in batch processing and continuous feeding. (c) *E*_C_ and *E*_H_ using the Fe porous media catalyst with electromagnetic induction monolith heating and values in previous studies. HDPE: high-density polyethylene.

We next evaluated the cycling performance of the Fe metal porous catalyst at a plastic-to-catalyst mass ratio of 1:2, avoiding excessive catalyst loading and ensuring that the catalyst reached an equilibrium state (Fig. 3a). The experimental results revealed no significant decline in the C yield (701.01–717.12 mg·g^−1^ plastic, Fig. S15) and H_2_ yield (48.55–50.89 mmol·g^−1^ plastic, Fig. 3a) after 10 cycles. However, with an increasing cycle number, a moderate decrease in CNT selectivity was observed, declining by 4 wt% from 94.64 to 90.64 wt% (Fig. S15), leading to a corresponding decrease in the CNT yield from 675.34 to 646.48 mg·g^−1^ plastic. Despite this selectivity decrease, the overall cycling performance remains acceptable and competitive compared with reported catalysts in the literature [14]. Surface-roughness measurements indicated no notable change in the catalyst before and after the reaction (Fig. S16). We conducted further pyrolysis–catalysis experiments under larger-scale continuous-feeding conditions (Fig. S2), which are considered key for evaluating the scalability of plastic-waste processing [41]. A total of 25.5 g of plastic samples were processed within 60 min, matching the batch-processing rate. The C (*E*_C_) and H (*E*_H_) atom-recovery efficiencies increased from 85.63% and 72.97% under batch processing to 89.39% and 82.98%, respectively, under continuous feeding (Fig. 3b and Fig. S17), likely due to the higher local heating rate of the C-deposit layer [33,42]. The maintenance of high *E*_C_ and *E*_H_, along with a >10-fold increase in the plastic-processing capacity, confirms the scalability of catalytic reforming via monolithic heating.

Figure 3c shows a comparison of *E*_C_ and *E*_H_ values obtained by using the Fe porous media catalyst at a plastic-to-catalyst mass ratio of 1:4 with electromagnetic induction monolith heating against values reported in previous studies. To the best of the current knowledge, the *E*_C_ values achieved in this study are the highest reported to date. In addition, our experimental results revealed that the highest catalyst mass activity (i.e. product yield per unit mass of catalyst) is achieved at a plastic-to-catalyst mass ratio of 1:0.5, which was consistent with trends reported in the literature (Table S2). Notably, the obtained *E*_C_ remained higher than or comparable to the highest values reported in previous studies under similar conditions. These results demonstrated that the high carbon efficiency achieved in this work was not simply a consequence of excessive catalyst usage, but reflected the intrinsic effectiveness of the induction-heated metal porous catalyst system. Also, the CNTs obtained in this work exhibited a lower defect density and improved purity compared with those reported in previous studies (Table S3).

The pyrolysis–catalysis process efficiently converted WPE and WPP into CNTs, with CNT proportions reaching 96.55 and 98.65 wt%, respectively (Fig. S18). This high performance was maintained despite the notable ash contents of WPE and WPP (3.07 and 15.74 wt%, respectively, Table 1) and the considerable solid fractions formed during first-stage pyrolysis (3.95 and 17.00 wt%, respectively, Fig. S18). Elemental analysis shows that the ash fraction is dominated by Si (primarily as SiO_2_), Ca, Al and Mg, which together account for ∼90 wt% of the total metal content (Table S5), in good agreement with reported literature data [31]. The ash contents were retained in the solid residue during the initial pyrolysis stage and did not volatilize into the pyrolysis vapors. As a result, they did not participate in or interfere with the subsequent catalytic reforming process, which was responsible for CNT formation. Consequently, the presence of ash in the feedstock did not adversely affect catalyst longevity, CNT yield or product purity. The *E*_C_ values of WPE and WPP were enhanced by approximately 30 and 20 wt%, respectively, compared with the highest previously reported values. This outcome is significant for the recycling of real-world waste plastics, as it confirms that the presence of impurities does not adversely affect the yield or quality of CNTs produced through monolithically heated pyrolysis reforming. The *E*_H_ values for various types of plastics also reached the highest levels among the existing reports, with the exception of PP. This exception can be attributed to the use of an oxygen-containing catalyst in [S1], which promoted the oxidation of C deposits, resulting in the formation of CO_2_. The generated CO_2_ subsequently reacted with CH_4_ to produce additional H_2_ and CO (CO_2_ + CH_4_ → 2H_2_ + 2CO), thereby enhancing the H_2_ yield. However, the catalytic activity in that study declined after five cycles due to oxygen depletion in the catalyst, necessitating periodic reactivation. Such a requirement limits the long-term stability and practical applicability of the catalyst. In contrast, our study demonstrated consistent H_2_ production, with the total H_2_ yield increasing to 997.6 mmol over 10 cycles (Fig. 3a), confirming the superior durability and efficiency of the catalytic system. In summary, this study demonstrates that an oxygen-free, base-metal porous catalyst can achieve high C and H atom-recovery efficiencies while maintaining stable and effective catalytic performance.

**Table 1.** Ultimate and proximate analyses of plastics.

	Ultimate analysis (wt%)					Proximate analysis (wt%)			
Plastics	C	H	N	S	O	Volatiles	Ash	Fixed carbon	Moisture
LDPE	83.84	14.00	0.00	0.00	1.10	98.93	0.00	1.07	0.00
HDPE	83.79	13.97	0.00	0.00	0.63	98.39	0.00	1.61	0.00
PP	84.01	14.17	0.00	0.00	1.15	99.33	0.67	0.00	0.00
WPE	82.17	12.81	0.04	0.00	1.91	96.06	3.07	0.87	0.00
WPP	69.16	9.71	0.49	0.00	4.90	80.49	15.74	3.77	0.00

### Characterization of produced CNTs

The CNTs obtained from the monolithically heated pyrolysis-reforming process were comprehensively characterized (Figs S19–S34) to evaluate their potential for commercial applications. Transmission electron microscopy confirmed the formation of multi-walled CNTs, with no single-walled structures detected. Elevated temperatures led to an increase in the average outer diameter from 23.59 to 36.19 nm and promoted clustering behavior (Fig. S19), attributed to an increased number of graphene layers (Table S7). The crystallite size *D*_C_ was also found to increase from 14.13 to 17.22 nm with temperature, while the lattice spacing *d*_(002)_ remained unchanged. Consequently, the average number of graphene layers *n* = *D*_C_/*d*_(002)_ rose from 41.80 to 50.95. The highest graphitization degree (*g*), determined by using X-ray diffraction (XRD), was observed at 900°C. Raman spectroscopy revealed a decreasing *I*_D_/*I*_G_ ratio and an increasing *I*_G'_/*I*_G_ ratio with temperature, indicating reduced structural defects and enhanced graphitization. CNTs synthesized using Fe, Fe_5_Ni_5_ and Ni porous catalysts exhibited average outer diameters of 38.06, 29.18 and 24.93 nm, respectively (Fig. S20). The presence of Fe has been found to enlarge the crystallite size *D*_C_ and increase the number of graphene layers *n* [43], resulting in larger CNT diameters. Moreover, the formation of Fe_3_C (#35–0772, Fig. S34) facilitated the transformation of amorphous C into filamentous C [15]. The Fe catalyst yielded optimal Raman ratios (*I*_D_/*I*_G_ = 0.47, *I*_G'_/*I*_G_ = 0.52), signifying high structural quality and graphitization (Fig. S28). A lattice spacing *d*_(002)_ of 0.338 nm was confirmed by using high-resolution transmission electron microscopy (Fig. S31), consistent with the XRD results (Table S8). Additionally, high-angle annular dark field imaging revealed that the elemental distributions corresponded closely with the CNT morphology (Fig. S31B). Increasing the plastic-to-catalyst mass ratio from 1:0.5 to 1:4 led to a decrease in the *I*_D_/*I*_G_ ratio and an overall increasing tendency in the *I*_G'_/*I*_G_ ratio at high plastic-to-catalyst mass ratios ratios (Fig. S29), accompanied by an increase in the graphitization degree (*g*) (Table S9), indicating an overall improvement in the CNT quality. However, excessive catalyst loading (plastic-to-catalyst mass ratio of 1:40) had a negligible effect on further enhancing the CNT quality.

CNTs synthesized from waste plastics, such as WPE and WPP, exhibited smaller average outer diameters, measuring 27.88 and 24.33 nm, respectively, compared with those obtained from virgin plastics. This reduction in diameter was attributed to smaller crystallite sizes and fewer graphene layers (Fig. S22 and Table S10). Raman spectroscopy showed higher *I*_D_/*I*_G_ ratios (0.50 for WPE and 0.67 for WPP) and lower *I*_G'_/*I*_G_ ratios (0.60 for WPE and 0.57 for WPP), indicating a lower graphitization degree and increased structural defects relative to CNTs derived from virgin plastics (Fig. S28). However, the CNT selectivity in C deposits from waste plastics was found to be comparable to that from virgin plastics (Fig. S18).

**Figure 4.**
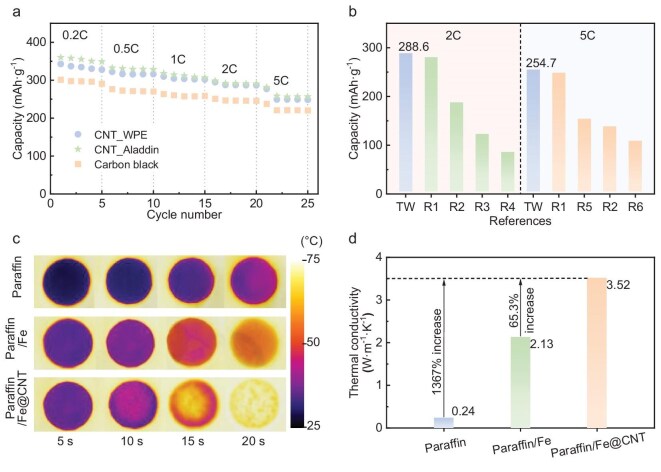
Applications of WPE-derived CNTs. (a) Rate performance of carbon anodes with conductive materials of CNT_WPE, CNT_Aladdin and carbon black for sodium-ion batteries. (b) Capacity of carbon anodes with conductive materials of CNT_WPE and other carbon materials in previous studies. (c) Temperature distributions of paraffin, paraffin/Fe and paraffin/Fe@CNT under constant boundary temperature heating at 70°C. (d) Thermal conductivities of paraffin, paraffin/Fe and paraffin/Fe@CNT. CNT_WPE and CNT_Aladdin represent CNTs obtained from WPE and commercial products of Aladdin. Paraffin/Fe and paraffin/Fe@CNT represent paraffin with pure Fe porous media and CNT-coated Fe porous media (post-reaction Fe porous catalyst).

The global CNT market is undergoing significant expansion, primarily driven by rising demand for conductive materials in batteries and energy-storage systems due to the superior electrical and thermal conductivity of CNTs [44,45]. Multi-walled CNTs currently dominate both sales volume and production capacity, with the market projected to grow from $5.25 billion in 2021 to $10.75 billion by 2028, reflecting a compound annual growth rate of 10.8% [46]. In this study, CNTs derived from waste plastics were evaluated as conductive additives in hard C anodes for sodium-ion batteries (Fig. S35) and as thermal-conductivity enhancers in phase-change materials (Fig. S36). The electrochemical performance of these CNTs was compared with those of commercial multi-walled CNTs and C black. The commercial CNTs showed a higher graphitization degree (*g* = 0.75 vs. 0.64, Table S10) and a lower defect density (*I*_D_/*I*_G_ = 0.31 vs. 0.50, Fig. S30) than the CNTs derived from waste plastics, indicating the generally higher structural quality of the commercial CNTs. However, compared with CNTs produced from waste plastics in previous studies, the CNTs obtained in this work exhibited a lower *I*_D_/*I*_G_ ratio and a higher CNT proportion, indicating superior structural quality (Table S3). The CNTs from waste plastics exhibited slightly lower rate performance than commercial CNTs but outperformed C black (Fig. 4a). Notably, the hard C anode incorporating CNTs derived from WPE delivered a significantly higher specific capacity at high current densities compared with anodes using other advanced C materials, such as the expanded graphite reported in R1 [47] (Fig. 4b). The specific capacity was nearly 100 mAh·g^−1^ greater than those of anodes composed of conventional hard C materials (Table S11). This improvement was attributed to the synergistic interaction between the CNTs and the hard C matrix, which enhances sodium-ion transport and storage efficiency.

The paraffin/Fe@CNT composite exhibited superior performance as a heat-transfer-enhancement medium for phase-change materials (Fig. 4c and d). Compared with pure paraffin and paraffin/Fe composites, paraffin/Fe@CNT achieved significantly higher thermal conductivity, reaching 3.52 W·m^−1^·K^−1^—an increase of ∼1367% relative to that of pure paraffin. Infrared thermal imaging (Fig. 4c) showed that paraffin/Fe@CNT enabled rapid and uniform heat distribution within 20 s of heating at 70°C, substantially outperforming the other two materials. This enhanced thermal performance can be attributed to the synergistic interaction between the CNT coating and the porous Fe framework. The CNTs created additional heat-conduction pathways, effectively lowering thermal resistance and facilitating rapid energy transfer throughout the phase change material matrix [48]. Consequently, the Fe@CNT composite successfully mitigated the inherent thermal-conductivity limitations of paraffin, delivering improved heat-transfer efficiency and a faster thermal response. These findings highlight the strong potential of this composite for applications requiring efficient thermal energy storage and management.

### Techno-economic analysis of plastic-waste pyrolysis–catalysis for CNT production

**Figure 5.**
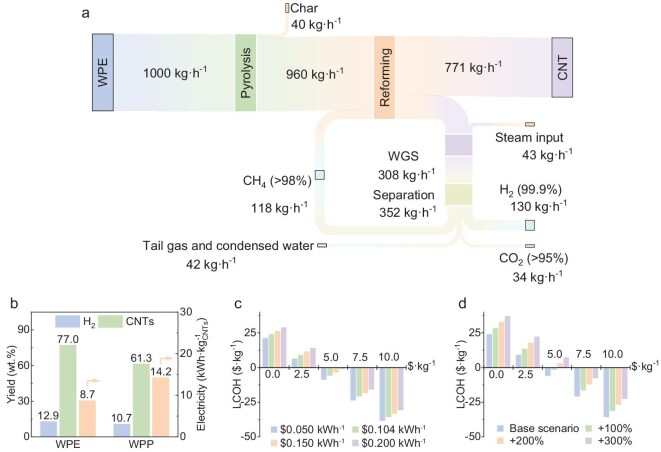
TEA for process of converting plastic waste into CNTs and H_2_. (a) Mass balance diagram of the proposed co-production system utilizing WPE as feedstock, derived from process simulation. (b) Comparison of required electricity, CNT yield and H_2_ yield when using WPE versus WPP as feedstock. In the simulated reformer, the conversion rate of C atoms in the feedstock to CNTs is based on experimental results, while the gas-fraction yields are determined by using an equilibrium approach at 900°C. (c) Estimated LCOH ($·kg^−1^) produced from WPE as a function of electricity price ($·kWh^−1^) assuming induction-heater efficiency of 90%, calibrated to meet different levels of the CNT selling price at $2.5-kg^−1^ intervals (*x*-axis). (d) Estimated LCOH ($·kg^−1^) produced from WPE as a function of the capital cost of an induction-heated reformer (% of original cost), evaluated at varying levels of CNT selling price with the electricity price set at $0.104 kWh^−1^.

We developed a process model to conduct techno-economic analysis (TEA) of a system designed for the co-production of CNTs and H_2_, with H_2_ as a valuable byproduct. The proposed system, depicted in Fig. S37, consists of a pyrolyser, an induction-heated reformer, a water–gas shift module and a pressure swing adsorption unit for gas separation. The TEA focuses exclusively on WPE and WPP as feedstocks due to their relevance to real-world unrecoverable waste streams, which are not typically suitable for mechanical recycling and present significant management challenges. A feed rate of 1000 kg·h^−1^ was applied for comparative purposes. When WPE was used, the system produced 771 kg·h^−1^ of CNTs and 130 kg·h^−1^ of H_2_ (Fig. 5a). Due to its higher ash content, WPP resulted in lower product yields. Accordingly, the specific electricity consumption for CNT production was lower for WPE (8.7 kWh·kg^−1^ CNT) compared with that for WPP (14.2 kWh·kg^−1^ CNT) (Fig. 5b). Further details regarding the process model architecture, simulation parameters, assumptions and methodological procedures used in the TEA are provided in the Supplementary information.

An economic assessment was performed for both the WPE and WPP feedstocks based on mass and energy balances derived from the process model. The evaluation included estimations of total capital investment (TCI), operating expenses (OPEX), the projected selling price of CNTs and the levelized cost of H_2_ (LCOH). For a system processing 1000 kg·h^−1^ of WPE, the TCI was estimated at ∼$48.9 million (Fig. S38). The major capital expenditures were attributed to the pyrolysis and reforming units (62.6%), followed by gas-separation systems (23.4%) and water–gas shift reactors (10.1%). The annual OPEX were projected at $20.2 million, with the most significant contributions arising from reformer catalyst costs (49.7%), electricity (27.8%) and fixed costs including labor, maintenance and insurance (18.1%). The cost of plastic-waste feedstock contributed a relatively minor portion (4.5%) (Fig. S38). These calculations assumed baseline waste-plastics prices of $112.7 ton^−1^ and an electricity rate of $0.104 kWh^−1^, reflecting current values in China. The catalyst-consumption rates were derived from experimental results, while the material costs used for synthesizing the catalyst were based on the data in Table S15. Under these assumptions, the catalyst expenses were estimated at $1255.3 per ton of WPE and $1021.8 per ton of WPP. Consequently, the reforming unit remains the primary driver of the OPEX. Reducing both the catalyst-consumption rate and the production cost is therefore critical for improving the economic viability of this process in future scale-up implementations.

This study assessed the required CNT price and LCOH needed to achieve a net present value of zero over a 25-year plant lifespan, assuming a discount rate of 8%. In the base-case scenario using WPE feedstock (priced at $112.7 ton^−1^), with electricity at $0.104 kWh^−1^ and an induction-heater efficiency of 90%, the break-even LCOH of $0 kg^−1^ can be achieved when CNTs are sold at $4.02 kg^−1^ (Fig. 5c). When WPP is used as the feedstock, the minimum required CNT selling price increases to $4.87 kg^−1^ to reach the same break-even condition for H_2_ production. These results indicate that, even at relatively low CNT prices, revenue from CNT sales is sufficient to offset the H_2_-production costs. Currently, the market price for powdered multi-walled CNTs is ∼$100 kg^−1^, especially for applications in high-voltage cables, semiconductors and electromagnetic shielding. CNT paste (5% CNT, 95% *N*-methyl pyrrolidone), used widely in the battery industry, is priced at between $5 and $10 kg^−1^ [49]. Therefore, the proposed plastic-waste-to-CNT route offers a considerable cost advantage, supporting competitive market entry. Conversely, a CNT price near $0 kg^−1^ would result in an LCOH exceeding $23 kg^−1^, which is substantially higher than the LCOH of $1.5–2.0 kg^−1^ that is typical for fossil-fuel-derived H_2_ (gray H_2_). This highlights that, without CNT revenue, the economic feasibility of the process would be significantly compromised.

The LCOH is significantly affected by electricity prices and CNT market value. We estimated the LCOH ($·kg^−1^) across a range of electricity prices from $0.050 to $0.200 kWh^−1^—encompassing the median electricity price in the EU in 2023 [50]—under an assumed induction-heater efficiency of 90% (Fig. 5c). The analysis considered CNT selling prices from $0.0 to $10.0 kg^−1^, in $2.5 kg^−1^ increments. The results demonstrate that, when CNTs hold little or no market value, the LCOH remains substantially above $21 kg^−1^, far exceeding the cost of fossil-fuel-based H_2_. However, as the CNT price increases, the LCOH declines sharply and becomes negative when the CNT price exceeds $5.0 kg^−1^. These findings indicate that the co-production of CNTs at a competitive price can render H_2_ production from WPE economically viable, even with elevated electricity costs.

Figure 5d presents a sensitivity analysis of the capital cost of the induction-heated reformer on the LCOH, expressed as a percentage of the baseline capital expenditure. We performed this analysis at a fixed electricity price of $0.104 kWh^−1^ while varying the CNT selling prices. The results indicate that increases in the capital cost raise the LCOH; however, the trend mirrors that observed in the electricity price analysis: as the CNT prices rise, the LCOH declines. Specifically, when the CNT prices reach ≥$7.5 kg^−1^, the LCOH becomes negative across all capital-cost scenarios, signifying profitability even with elevated initial investment. Considering that the current market price for multi-walled CNTs ranges from $340 to $25 000 kg^−1^, the CNTs produced via waste-plastics pyrolysis–catalysis offer substantial cost advantages [44]. This analysis highlights that WPE-based H_2_ production remains economically viable when CNTs are marketed at competitive prices. The capital expenditure of the induction-heated reformer introduces cost uncertainty; however, its effect on the LCOH is significantly offset when CNTs are sold at elevated market prices. Overall, the analysis indicates that WPE represents a potentially economically sustainable feedstock for H_2_ production, especially in markets characterized by strong CNT demand and favorable pricing.

## CONCLUSIONS

This work demonstrated the effectiveness of novel electromagnetic induction heating in enhancing the pyrolysis–catalysis of plastic waste for CNT and H_2_ production. The technique enabled rapid monolithic heating of the metal porous catalyst with substantially lower energy consumption than conventional electric heating. It also promoted more uniform temperature distribution and shortened reaction times, and improved the overall efficiency of the catalytic reforming process. Among the metal porous catalysts evaluated (Ni, Fe and Fe–Ni alloy), the Fe porous catalyst exhibited the highest efficiency for CNT production, attributed to its strong chemical adsorption of C atoms that facilitated the conversion of amorphous C into CNTs. In contrast, Ni achieved the highest H_2_ selectivity, while the Fe–Ni alloy delivered intermediate performance, indicating inherent trade-offs between CNT yield and H_2_ production based on the catalyst composition. A continuous-feeding pyrolysis–catalysis system incorporating monolithic heating processed 25.5 g of plastics—over an order of magnitude higher than the batch mode—while maintaining high *E*_C_ and *E*_H_ (89.39% and 82.98%, respectively), indicating its scalability. Furthermore, the presence of impurities (dirt and metals) in waste plastics showed minimal influence on the CNT yield or quality, underscoring the viability of this approach for recycling real-world plastic waste.

The TEA of plastic-waste pyrolysis–catalysis for CNTs and H_2_ production confirmed the economic feasibility of utilizing WPE and WPP as feedstocks. Although the process was sensitive to electricity pricing and induction-heater efficiency, the estimated CNT selling price remained well below the current market levels across a range of capital-cost scenarios, reinforcing the economic viability of this waste-to-resource strategy. Overall, the integration of electromagnetic induction heating with metal porous catalysts, particularly Fe, demonstrated a robust, scalable and energy-efficient pathway for sustainable plastic-waste valorization. Our approach enables both effective waste reduction and the generation of valuable materials, such as CNTs and H_2_, supporting its potential for large-scale deployment.

## METHODS

### Materials

LDPE, high-density polyethylene, PP, WPE and WPP were supplied by Zhoushan Jinke Renewable Resources Co., Ltd, China. Waste plastics, sourced from municipal solid waste with particle sizes of ∼3 mm, were dried at 100°C for 24 h in a vacuum oven to eliminate moisture before pyrolysis reforming. Ultimate and proximate analyses of the plastic samples are presented in Table 1. Metal porous catalysts composed of Fe, Fe_5_Ni_5_ and Ni were obtained from Kunshan Baiyida Electronic New Materials Co., Ltd, China. The cylindrical catalysts measured 43 mm in diameter and 10 mm in height, with a porosity of 0.97. They were categorized by pore densities of 10, 20 and 30 PPI. The commercial CNTs were provided by Aladdin Biochemical Technology Co., Ltd, Shanghai, China (CAS No. 308068-56-6).

### Experimental design

CNT and H_2_ were produced from waste plastics via pyrolysis–catalysis in a tandem fixed-bed system comprising four integrated units: a gas-supply module, a pyrolysis chamber, a catalytic reforming unit and a gas-collection module (Fig. S1). Waste plastics were initially pyrolysed in a dedicated unit comprising a copper crucible and a resistance wire heating element, generating pyrolysis volatiles. Control experiments confirmed that the choice of crucible material had a negligible influence on the experimental results (Fig. S39) and that the pyrolysis process did not affect the operating temperature of the downstream reforming stage (Fig. S40). These volatiles were subsequently decomposed in a catalytic reforming unit to produce CNTs and H_2_. The reforming unit was equipped with a variable-frequency power supply, an electromagnetic induction-heating coil, a water cooler and metal porous catalysts. Plastic samples (2 g) were placed in the crucible (18 mm inner diameter, 1 mm wall thickness, 55 mm height). Before pyrolysis, argon gas was purged through the system at 300 mL·min^−1^ for 20 min to establish an inert atmosphere and then maintained at 25 mL·min^−1^ during the process. The catalytic reforming unit, containing 20 catalyst pieces, was preheated to the target temperature by using electromagnetic induction. Once stabilized, the pyrolysis unit was heated to 500°C at a rate of 10°C·min^−1^, based on the thermal decomposition behavior of the plastics (Fig. S41). Gas products were collected in gas bags and analysed by using gas chromatography.

A continuous-feeding plastic pyrolysis–catalysis system was constructed to demonstrate the scalability of the process (Fig. S2). Molten salt (58 wt% LiCl + 42 wt% KCl, melting point 350°C) was employed as a heat-transfer medium in the pyrolysis reactor. Before the experiment, the molten salt was heated to 500°C, while the Fe porous catalyst in the reforming reactor was preheated to 900°C. Plastic samples were fed into the pyrolysis reactor via a screw feeder at a rate of ∼0.4 g·min^−1^, matching the batch-processing throughput. Upon contact with the molten salt, the plastics decomposed into pyrolysis volatiles, which were subsequently directed into the reforming reactor for catalytic conversion into CNTs and H_2_. A total of 25.5 g of plastic waste was processed over 60 min in the continuous-feeding experiment.

### Experimental data analysis

The gaseous products primarily consisted of H_2_, CO, CH_4_, C_2_H_4_ and C_2_H_6_. No liquid oil or wax was detected in the products when the catalytic reforming temperature exceeded 700°C. The total mass of gaseous products was calculated based on the principle of mass conservation, as follows:


(1) \begin{eqnarray*}
{m}_{\mathrm{g}} = {m}_{\mathrm{p}} - {m}_{\mathrm{s}} - {m}_{\mathrm{c}},
\end{eqnarray*}

where *m*_g_, *m*_p_, *m*_s_ and *m*_c_ represent the masses of the gas products, plastic feed, catalytic reforming solids and pyrolysis char, respectively.

The mass of the produced CNTs, *m*_CNT_, was determined as follows:


(2) \begin{eqnarray*}
{m}_{{\mathrm{CNT}}} = {m}_s \cdot {w}_{{\mathrm{CNT}}},
\end{eqnarray*}

where *w*_CNT_ denotes the mass fraction of CNTs in the solid products, determined by using temperature-programmed oxidation analysis.

The selectivity of the CNTs, defined as the proportion of CNTs in the solid products, is given by:


(3) \begin{eqnarray*}
{x}_{{\mathrm{CNT}}} = {w}_{{\mathrm{CNT}}} \times 100{\mathrm{\% }}.
\end{eqnarray*}

The CNT yield *Y*_CNT_ (mg·g^−1^ plastic) was determined as follows:


(4) \begin{eqnarray*}
{Y}_{{\mathrm{CNT}}} = \frac{{{m}_{{\mathrm{CNT}}}}}{{{m}_p}} \times 1000.
\end{eqnarray*}

The selectivity of each gas component *x_i_* (%) was calculated as follows:


(5) \begin{eqnarray*}
{x}_i = \frac{{{V}_i}}{{\sum {V}_i}} \times 100{\mathrm{\% }},
\end{eqnarray*}

where *V_i_* represents the volume of gas component *i* (H_2_, CO, CH_4_, C_2_H_4_ and C_2_H_6_).

The yield of each gas component *Y_i_* (mmol·g^−1^ plastic) was defined as follows:


(6) \begin{eqnarray*}
{Y}_i = 1000 \times \frac{{{m}_{\mathrm{g}}}}{{{m}_{\mathrm{p}}}} \times \frac{{{V}_i}}{{\sum {V}_i{M}_i}},
\end{eqnarray*}

where *M_i_* denotes the molar mass of gas component *i*.

The production efficiency of the CNTs and H_2_ from various plastics was evaluated by calculating the atom-recovery efficiencies for C (*E*_C_) and H_2_ (*E*_H_), as follows:


(7) \begin{eqnarray*}
{E}_{\mathrm{C}} = \frac{{{m}_{{\mathrm{CNTs}}}}}{{m_{{\mathrm{plastic}}}^{\mathrm{C}}}} \times 100{\mathrm{\% }},
\end{eqnarray*}

(8) \begin{eqnarray*}
{E}_{\mathrm{H}} = \frac{{{m}_{{{\mathrm{H}}}_2}}}{{m_{{\mathrm{plastic}}}^{\mathrm{H}}}} \times 100{\mathrm{\% }},
\end{eqnarray*}

where ${m}_{{\mathrm{CNTs}}}$, $m_{{\mathrm{plastic}}}^{\mathrm{C}}$, ${m}_{{{\mathrm{H}}}_2}$ and $m_{{\mathrm{plastic}}}^{\mathrm{H}}$ represent the mass of the produced CNTs, the C content in the plastic, the mass of the produced H_2_ and the H content in the plastic, respectively.

### Computational methods

Computational fluid dynamics (CFD) simulations were performed by using COMSOL Multiphysics 6.0 to evaluate the heat and mass transfer during the catalytic reforming of the plastic pyrolysis volatiles under electromagnetic induction heating. DFT calculations were conducted by using the Vienna *ab initio* simulation package to investigate the catalytic reforming mechanisms of the plastic pyrolysis products on various Fe–Ni metal catalysts. Additionally, Aspen Plus was employed to construct the process flowsheet for the TEA and determine the mass and energy balances used for subsequent cost and economic assessments.

## Supplementary Material

nwag143_Supplemental_File

## Data Availability

All data are available in the main text and/or the Supplementary materials.

## References

[1] Conk RJ, Stahler JF, Shi JX et al. Polyolefin waste to light olefins with ethylene and base-metal heterogeneous catalysts. Science 2024; 385: 1322–7. 10.1126/science.adq7316 39208080

[2] Yadav G, Singh A, Dutta A et al. Techno-economic analysis and life cycle assessment for catalytic fast pyrolysis of mixed plastic waste. Energy Environ Sci 2023; 16: 3638–53. 10.1039/D3EE00749A

[3] Cottom JW, Cook E, Velis CA. A local-to-global emissions inventory of macroplastic pollution. Nature 2024; 633: 101–8. 10.1038/s41586-024-07758-6 39232151 PMC11374682

[4] Singh N, Hui D, Singh R et al. Recycling of plastic solid waste: a state of art review and future applications. Compos Part B-Eng 2017; 115: 409–22. 10.1016/j.compositesb.2016.09.013

[5] Qin J, Wu F, Dou Y et al. Advanced catalysts for the chemical recycling of plastic waste. Adv Mater 2025; 37: 2418138. 10.1002/adma.202418138 39748624

[6] Ragaert K, Delva L, Van Geem K. Mechanical and chemical recycling of solid plastic waste. Waste Manage 2017; 69: 24–58. 10.1016/j.wasman.2017.07.044 28823699

[7] Lombardi L, Carnevale E, Corti A. A review of technologies and performances of thermal treatment systems for energy recovery from waste. Waste Manage 2015; 37: 26–44. 10.1016/j.wasman.2014.11.010 25535103

[8] Huang J, Veksha A, Chan WP et al. Chemical recycling of plastic waste for sustainable material management: a prospective review on catalysts and processes. Renew Sust Energ Rev 2022; 154: 111866. 10.1016/j.rser.2021.111866

[9] Jiao X, Zheng K, Hu Z et al. Conversion of waste plastics into value-added carbonaceous fuels under mild conditions. Adv Mater 2021; 33: 2005192. 10.1002/adma.202005192 33834571

[10] Rahimi A, García JM. Chemical recycling of waste plastics for new materials production. Nat Rev Chem 2017; 1: 0046. 10.1038/s41570-017-0046

[11] Jehanno C, Alty JW, Roosen M et al. Critical advances and future opportunities in upcycling commodity polymers. Nature 2022; 603: 803–14. 10.1038/s41586-021-04350-0 35354997

[12] Duan J, Chen W, Wang C et al. Coking-resistant polyethylene upcycling modulated by zeolite micropore diffusion. J Am Chem Soc 2022; 144: 14269–77. 10.1021/jacs.2c05125 35914188

[13] Bi T, Chen Y, Lin L et al. Closed-loop recycling of polyethylene to ethylene and propylene via a kinetic decoupling–recoupling strategy. Nat Chem Eng 2025; 2: 650–61. 10.1038/s44286-025-00290-y

[14] Liu Q, Jiang D, Zhou H et al. Pyrolysis–catalysis upcycling of waste plastic using a multilayer stainless-steel catalyst toward a circular economy. Proc Natl Acad Sci USA 2023; 120: e2305078120. 10.1073/pnas.2305078120 37695879 PMC10523629

[15] Acomb JC, Wu C, Williams PT. The use of different metal catalysts for the simultaneous production of carbon nanotubes and hydrogen from pyrolysis of plastic feedstocks. Appl Catal B-Environ 2016; 180: 497–510. 10.1016/j.apcatb.2015.06.054

[16] Li Q, Yan H, Zhang J et al. Effect of hydrocarbons precursors on the formation of carbon nanotubes in chemical vapor deposition. Carbon 2004; 42: 829–35. 10.1016/j.carbon.2004.01.070

[17] Tessonnier JP, Su DS. Recent progress on the growth mechanism of carbon nanotubes: a review. ChemSusChem 2004; 4: 824–47. 10.1002/cssc.201100175 21732543

[18] Yao D, Wu C, Yang H et al. Co-production of hydrogen and carbon nanotubes from catalytic pyrolysis of waste plastics on Ni-Fe bimetallic catalyst. Energy Convers Manage 2017; 148: 692–700. 10.1016/j.enconman.2017.06.012

[19] Wu C, Nahil MA, Miskolczi N et al. Processing real-world waste plastics by pyrolysis-reforming for hydrogen and high-value carbon nanotubes. Environ Sci Technol 2014; 48: 819–26. 10.1021/es402488b 24283272

[20] Kwon S, Kang J, Lee B et al. Nonviable carbon neutrality with plastic waste-to-energy. Energy Environ Sci 2023; 16: 3074–87. 10.1039/D3EE00969F

[21] Williams PT . Hydrogen and carbon nanotubes from pyrolysis-catalysis of waste plastics: a review. Waste Biomass Valori 2021; 12: 1–28. 10.1007/s12649-020-01054-w

[22] Jie X, Li W, Slocombe D et al. Microwave-initiated catalytic deconstruction of plastic waste into hydrogen and high-value carbons. Nat Catal 2020; 3: 902–12. 10.1038/s41929-020-00518-5

[23] Zhang S, Jiang SF, Huang BC et al. Sustainable production of value-added carbon nanomaterials from biomass pyrolysis. Nat Sustain 2020; 3: 753–60. 10.1038/s41893-020-0538-1

[24] Cai N, Liu Q, Li X et al. Identify the impact of pyrolysis temperature on preparation of carbon nanotubes by catalytic reforming polypropylene. Waste Manage 2024; 190: 161–8. 10.1016/j.wasman.2024.09.016 39321601

[25] Miyazawa T, Kimura T, Nishikawa J et al. Catalytic performance of supported Ni catalysts in partial oxidation and steam reforming of tar derived from the pyrolysis of wood biomass. Catal Today 2006; 115: 254–62. 10.1016/j.cattod.2006.02.055

[26] Namioka T, Saito A, Inoue Y et al. Hydrogen-rich gas production from waste plastics by pyrolysis and low-temperature steam reforming over a ruthenium catalyst. Appl Energ 2011; 88: 2019–26. 10.1016/j.apenergy.2010.12.053

[27] He S, Li C, Sun H et al. Promotion of manganese on Fe-based catalyst for the production of carbon nanotubes (CNTs) from plastics. Chem Eng J 2024; 492: 152306. 10.1016/j.cej.2024.152306

[28] Liu X, Shen B, Wu Z et al. Producing carbon nanotubes from thermochemical conversion of waste plastics using Ni/ceramic based catalyst. Chem Eng Sci 2018; 192: 882–91. 10.1016/j.ces.2018.07.047

[29] Wong SL, Armenise S, Nyakuma BB et al. Plastic pyrolysis over HZSM-5 zeolite and fluid catalytic cracking catalyst under ultra-fast heating. J Anal Appl Pyrolysis 2023; 169: 105793. 10.1016/j.jaap.2022.105793

[30] López A, de Marco I, Caballero BM et al. Pyrolysis of municipal plastic wastes: influence of raw material composition. Waste Manage 2010; 30: 620–7. 10.1016/j.wasman.2009.10.014 19926462

[31] Li Y, Williams PT. Waste derived ash as catalysts for the pyrolysis-catalytic steam reforming of waste plastics for hydrogen-rich syngas production. J Anal Appl Pyrolysis 2024; 177: 106374. 10.1016/j.jaap.2024.106374

[32] Ren ZF, Huang Z, Xu JW et al. Synthesis of large arrays of well-aligned carbon nanotubes on glass. Science 1998; 282: 1105–7. 10.1126/science.282.5391.1105 9804545

[33] Yang H, Zaini IN, Pan R et al. Distributed electrified heating for efficient hydrogen production. Nat Commun 2024; 15: 3868. 10.1038/s41467-024-47534-8 38719793 PMC11078997

[34] He L, Liao G, Hu S et al. Effect of temperature on multiple competitive processes for co-production of carbon nanotubes and hydrogen during catalytic reforming of toluene. Fuel 2020; 264: 116749. 10.1016/j.fuel.2019.116749

[35] Ramirez A, Royo C, Latorre N et al. Unraveling the growth of vertically aligned multi-walled carbon nanotubes by chemical vapor deposition. Mater Res Express 2014; 1: 045604. 10.1088/2053-1591/1/4/045604

[36] Koponen A, Kataja M, Timonen J. Permeability and effective porosity of porous media. Phys Rev E 1997; 56: 3319–25. 10.1103/PhysRevE.56.3319

[37] Yamanaka A, Jono R, Tejima S et al. Molecular dynamics simulation of carbon nanotube growth under a tensile strain. Sci Rep 2024; 14: 5625. 10.1038/s41598-024-56244-6 38454043 PMC10920857

[38] Liu X, Zhang Y, Nahil MA et al. Development of Ni- and Fe-based catalysts with different metal particle sizes for the production of carbon nanotubes and hydrogen from thermo-chemical conversion of waste plastics. J Anal Appl Pyrolysis 2017; 125: 32–9. 10.1016/j.jaap.2017.05.001

[39] Lee CJ, Park J, Jeong AY. Catalyst effect on carbon nanotubes synthesized by thermal chemical vapor deposition. Chem Phys Lett 2002; 360: 250–5. 10.1016/S0009-2614(02)00831-X

[40] Sebestyén Z, Barta-Rajnai E, Bozi J et al. Thermo-catalytic pyrolysis of biomass and plastic mixtures using HZSM-5. Appl Energy 2017; 207: 114–22. 10.1016/j.apenergy.2017.06.032

[41] Dong Q, Lele AD, Zhao X et al. Depolymerization of plastics by means of electrified spatiotemporal heating. Nature 2023; 616: 488–94. 10.1038/s41586-023-05845-8 37076729

[42] Liu B, Slocombe DR, Wang J et al. Microwaves effectively examine the extent and type of coking over acid zeolite catalysts. Nat Commun 2017; 8: 514. 10.1038/s41467-017-00602-8 28894113 PMC5593951

[43] Ago H, Uehara N, Yoshihara N et al. Gas analysis of the CVD process for high yield growth of carbon nanotubes over metal-supported catalysts. Carbon 2006; 44: 2912–8. 10.1016/j.carbon.2006.05.049

[44] Modekwe HU, Olaitan AO, Onu MA et al. The Current Market for Carbon Nanotube Materials and Products: Handbook of Carbon Nanotubes. Cham: Springer International Publishing, 2021.

[45] Wyss KM, Li JT, Advincula PA et al. Upcycling of waste plastic into hybrid carbon nanomaterials. Adv Mater 2023; 35: 2209621. 10.1002/adma.202209621 36694364

[46] The Insight Partners. Multi-walled carbon nanotubes market demand, size & forecast by 2034. Market Research Report TIPRE00027438. https://www.theinsightpartners.com/reports/multi-walled-carbon-nanotubes-market (15 March 2026, date last accessed).

[47] Cheng Z, Zhang H, Cui J et al. Interlayer-expanded carbon anodes with exceptional rates and long-term cycling via kinetically decoupled carbonization. Joule 2025; 9: 101812. 10.1016/j.joule.2024.101812

[48] Wu S, Yan T, Kuai Z et al. Thermal conductivity enhancement on phase change materials for thermal energy storage: a review. Energy Storage Mater 2020; 25: 251–95. 10.1016/j.ensm.2019.10.010

[49] Liu K, Song W, Cui C et al. Process simulation of diesel into aromatics and carbon nanotubes: a techno and economic analyses. ACS Omega 2023; 8: 17941–7. 10.1021/acsomega.3c01135 37251164 PMC10210222

[50] Department for Energy Security and Net Zero. International non-domestic energy prices. Statistical Data Set QEP 5.4.1–5.4.4. https://www.gov.uk/government/statistical-data-sets/international-industrial-energy-prices (15 March 2026, date last accessed).

