# Supplementary material for: Enhancing carbon nanotubes production from pyrolysis–catalysis of plastic waste through monolithic heating
Source: Natl Sci Rev. 2026 Mar 9;13(10):nwag143. doi: 10.1093/nsr/nwag143 (PMC13240853; doi:10.1093/nsr/nwag143)
Supplement: nwag143_Supplemental_File [file nwag143_supplemental_file.pdf]

Supporting Information

Enhancing carbon nanotubes production from pyrolysis-catalysis of plastic waste through monolithic heating

Ruming Pan,† Jie Yu,† Youwei Yang,† Ilman Nuran Zaini,† Xibo He, Muhammad Rafique, Yibo Wu, Kuandong Jiang, Leilei Dai, Yi Fang, Youchuang Chao, Yuming Wen, Yanming Guo, Gérald Debenest, Weihong Yang, Yaning Zhang,* Yong Shuai,**

E-mail: Ruming Pan (ruming.pan@hit.edu.cn), Yaning Zhang (ynzhang@hit.edu.cn), Yong Shuai (shuaiyong@hit.edu.cn)

Ruming Pan, Jie Yu, Youwei Yang, and Ilman Nuran Zaini contributed equally to this work.

This PDF file includes:

Supplementary Text
Figures S1 to S41
Tables S1 to S15
References (1 to 76)

Supplementary Text

Experimental investigation of plastic pyrolysis-catalysis

We investigated the effects of catalytic temperature (700–900 °C), pore size of porous media (10–30 PPI, pore per inch), and porous media material (pure Fe, pure Ni, and Fe_5Ni_5 alloy) on the plastic pyrolysis-catalysis to determine the optimal operating conditions for the production of CNT. Low-density polyethylene (LDPE) was chosen to be the reactant as a representative plastic species.

CNT deposited on the metal catalyst was retrieved via mechanical vibration. Although mechanical vibration is effective for CNT detachment at the laboratory scale, its implementation in large-scale applications remains challenging due to engineering constraints associated with uniform vibration transmission and mechanical robustness. After each experiment, solid residues were removed from the pyrolysis crucible to prevent any potential interference with subsequent operations. Except for the catalyst cycling performance experiments, all other pyrolysis–reforming experiments were repeated at least twice to ensure the reliability of the experimental data.

We experimentally verified the mutual influence of pyrolysis and catalytic reforming temperatures to ensure the precise regulation of pyrolysis and reforming processes. The pyrolysis temperature was set to ramp from 20 °C to 500 °C at 10 $°C·min^{-1}$. Figure S40A shows that the practical pyrolysis temperature with induction heating was higher than the value without induction heating before 290 °C, attributing to the indirect heating of the high-temperature porous metal in the second-stage reforming process. The practical pyrolysis temperature with induction heating remained consistent with the value without induction heating and was increased from 290 °C to 500 °C at 10 $°C·min^{-1}$. Note that waste plastics begin to pyrolyze after 380 °C, so the second-stage induction heating would not affect the first-stage pyrolysis process of waste plastics. Also, the practical reforming temperatures were 900 °C with the pyrolysis heating and 898.8 °C without the pyrolysis heating, with an insignificant temperature difference. Therefore, the pyrolysis and reforming processes could maintain independent temperature control.

We conducted control experiments using an inert boron nitride (BN) crucible in place of the copper (Cu) crucible under otherwise identical pyrolysis conditions (Figure S39). The results show that when the Cu crucible was used as the pyrolysis chamber, the final solid yield was 76.08%, while a nearly identical solid yield of 76.07% was obtained using the BN crucible. Correspondingly, the H_2 selectively changed only marginally from 86.36 vol.% (Cu) to 86.44 vol.% (BN). Comparison of carbon efficiency (E_C) and hydrogen efficiency (E_H) further indicates minimal differences: E_C and E_H were 85.63% and 72.97% for the Cu crucible, compared with 85.05% and 73.19% for the BN crucible, respectively. Within experimental uncertainty, these results demonstrate that the use of a Cu crucible does not measurably influence pyrolysis behavior or downstream reforming performance, and that any potential catalytic contribution from copper is negligible under the conditions studied. Therefore, we conclude that the Cu crucible does not affect the validity of the experimental conclusions.

Characterizations of plastics, catalysts, produced CNT and gases

The plastic pyrolysis processes were identified by thermogravimetric analysis (TGA). This study characterized CNT via scanning electron microscope (SEM), transmission electron microscopy (TEM), X-ray diffractometer (XRD), Raman spectrometer, and high-angle annular dark field (HAADF). The catalyst elements were analyzed using SEM coupled with energy dispersive spectroscopy (EDS). Besides, the catalyst surface roughness was measured by 3D laser confocal microscope (LCM).

The elemental distribution of the catalysts was analyzed using energy-dispersive X-ray spectroscopy. The morphological features of the CNT and catalysts were characterized via a TESCAN AMBER SEM, while surface roughness before and after the reaction was evaluated using a Keyence color 3D laser confocal microscope. HRTEM was performed using a Talos F200 X operated at 200 kV to observe the detailed morphology and structure of CNT, with elemental mapping conducted through HAADF imaging. The lattice parameters of CNT were determined using a D8 ADVANCE XRD under a 40 kV working voltage, scanning from 10° to 90° at a rate of $5°\cdot min^{-1}$. Temperature-programmed oxidation (TPO) analysis was employed to assess CNT purity using an STA449F5 synchronous thermal analyzer. Samples were exposed to high-purity air at a flow rate of 60 $mL\cdot min^{-1}$, initially heated from 25°C to 110°C at $20°C\cdot min^{-1}$, and held for 10 min to remove moisture, followed by ramping to 800°C at $10°C\cdot min^{-1}$. Raman spectroscopy was conducted using a LabRAM HR evolution spectrometer with a 5 s integration time and an excitation wavelength of 532 nm. Spectra were collected in the 500–3000 cm^{-1} range. The intensity ratios of the D to G bands (I_D/I_G) and G' to G bands ($I_{G'}/I_G$) were used to evaluate the defect density and purity of the CNT.

The separated CNT powder was homogenized by mortar grinding to obtain a uniformly sample for analysis. For SEM sample preparation, approximately 10 mg of the homogenized CNT powder was directly sprinkled onto aluminum stubs covered with conductive double-sided carbon tape. Owing to the intrinsically high electrical conductivity of CNT, no additional conductive coating was required. For HRTEM sample preparation, about 1 mg of the homogenized CNT powder was dispersed in 10 mL of anhydrous ethanol and ultrasonicated for 15–20 min. A 3–5 μL aliquot of the resulting suspension was then drop-cast onto carbon-supported copper grids and allowed to dry naturally at room temperature in a clean Petri dish prior to imaging. All other characterizations were performed using directly CNT sample without special pretreatment.

The concentrations of H_2 and CO were determined using a SCION 8500 GC equipped with a flame ionization detector (FID), while CH_4, C_2H_4, and C_2H_6 were quantified using a thermal conductivity detector (TCD). The TCD and FID detectors are operated in parallel in the GC system. To ensure accurate quantification, we employed an external standard calibration method for absolute quantification. Prior to gas sample measurements, certified standard gas mixtures with precisely known concentrations were injected into the GC to establish linear calibration curves correlating peak area with absolute concentration for each target component.

Characterizations of plastics

This study conducted thermogravimetric analyses of LDPE, HDPE, and PP to determine the heat-up procedure for the pyrolysis process. Also, the thermogravimetric analyses of WPE and WPP have been conducted in the previous studies [1][2].

Table 1 lists the ultimate and proximate analyses of LDPE, HDPE, PP, WPE, and WPP. Note that WPE and WPP contain considerable amounts of ash with 3.07 wt.% and 13.23 wt.% respectively.

Characterizations of catalysts

The surface roughness of Fe porous media was determined by 3D LCM. Figure S16 shows that there was no significant change in catalyst surface roughness before and after the reaction.

We used SEM coupled with EDS to determine the Fe porous catalyst surface morphology and elemental distribution. Figure S6A reveals the microscopic characteristics of rough and uneven porous skeleton. The surface of porous catalyst before the reaction contained only Fe element, and the surface after the reaction contained 94.08 wt.% of C element and 5.92 wt.% of Fe element. The surface contained Fe element after the reaction was attributed to the precursor Fe_3C in the formation of CNT.

Characterizations of CNT

Raman spectroscopy is an analytical technique that can be used to characterize the quality of CNT. The D peak at ~1350 cm^{-1} corresponds to the disordered structure or defects in the graphite lattice, and the G peak at ~1600 cm^{-1} is related to the tangential vibration of the carbon atoms in the ordered graphite. The relative intensity ratio of the D and G bands, i.e., I_D/I_G, is an indicator of CNT quality, of which higher values of I_D/I_G are associated with fewer defects in the CNT as well as a higher degree of graphitization. The G' band at ~2705 cm^{-1} is associated with double resonance Raman scattering from double phonon emission. $I_{G'}/I_G$ ratio can be used to characterize the purity of CNT in the carbon deposit, with higher values representing higher CNT purity [3].

CNT as conductive materials in anodes for sodium-ion batteries (SIBs)

In this study, the active material employed in the anodes for sodium-ion batteries (SIBs) was commercial Type 2 hard carbon (KURANODE™) supplied by Kuraray Co., Ltd. For comparative analysis, two conductive materials were utilized. The first was Super P carbon black, produced by Imerys Graphite & Carbon. The second was multi-walled CNT supplied by Shanghai Aladdin Biochemical Technology Co., Ltd., with a CAS No. of 308068-56-6. These multi-walled CNT possess a purity exceeding 95%, an outer diameter ranging from 30 to 80 nm, and lengths between 0.5 and 2.0 μm.

As shown in Figure S35, the active material (commercial Type 2 hard carbon), polyvinylidene fluoride (PVDF) binder, and conductive materials (carbon black, commercial or WPE-derived CNT) were mixed in a mass ratio of 8:1:1 via mechanical stirring to form a homogeneous slurry. The slurry was subsequently cast onto copper foil current collectors and dried under vacuum at 60 °C for 24 h. The mass loading of active material was controlled within 1.0–1.5 $mg \cdot cm^{-2}$. Electrochemical characterization was conducted using CR2032-type coin cells, which were assembled in an argon-filled glovebox ($H_2O/O_2 < 0.1$ ppm). High-purity sodium foil served as the counter electrode, Whatman GF/D glass fiber as the separator, and 1 M sodium hexafluorophosphate ($NaPF_6$) in diglyme as the electrolyte. Galvanostatic charge–discharge tests were carried out at room temperature using a CT2001A test system (Wuhan Land Electronics Co., Ltd., China) within a voltage window of 0.01–2.5 V (vs. Na^+/Na).

CNT as thermal conductivity enhancers in phase change materials

In this study, paraffin wax (Product No. P00928) supplied by Shanghai Aladdin Co., Ltd. was employed as the phase change material (PCM). The key thermophysical properties of the paraffin are as follows: solidification temperature of 326 K, melting point of 327 K, latent heat of fusion of 174.6 $kJ \cdot kg^{-1}$, specific heat capacity of 3.22 $kJ \cdot kg^{-1} \cdot K^{-1}$, and thermal conductivity of 0.26 $W \cdot m^{-1} \cdot K^{-1}$. The Fe porous foam used in the composite PCM fabrication featured a diameter of 43 mm, a thickness of 10 mm, and a pore size of 30 PPI. Among the prepared composite PCMs, the mass fraction of Fe porous foam in the paraffin/Fe composite was 52 wt.%, while the mass fraction of Fe@CNT—CNT grown on Fe porous foam via pyrolysis-catalysis of WPE—in the paraffin/Fe@CNT composite was 57 wt.%.

The paraffin/Fe@CNT composite PCM was prepared by immersing post-reaction Fe porous catalyst—obtained via pyrolysis-catalysis of WPE—into molten paraffin wax. This impregnation process was conducted in a vacuum drying oven (Shanghai Yike Scientific Instrument Co., Ltd., model DZFD-6012) at 75 °C under a vacuum pressure of 0.03 MPa for 15 min. Subsequently, the composite PCM was extracted from the molten paraffin and allowed to cool at room temperature for 20 min, after which any excess paraffin on the surface was carefully removed.

For thermal performance evaluation, the bottom surface of the sample was placed directly onto a flat-plate heater (IKA, RCT basic) set to 70 °C. An infrared thermal imaging camera (FLUKE Ti450) was employed to monitor the overall temperature distribution and the temperature variation at the center of the sample. Measurements and thermal images were recorded at 5 s intervals. Identical procedures were applied to prepare and test paraffin/Fe and pure paraffin samples of the same dimensions to facilitate comparative analysis.

Computational fluid dynamics (CFD) simulation

This study conducted the CFD simulation of catalytic reforming of plastic waste pyrolysis volatiles by electromagnetic induction heating, involving the electromagnetic field, fluid mechanics, heat transfer, and chemical reactions.

Physical model

Figure S4A shows the physical model of catalytic reforming of plastic waste pyrolysis volatiles with electromagnetic induction monolith heating. To achieve geometric symmetry in the simulation model, the distance from the porous medium to the outlet was increased from the experimental value of 100 mm to 200 mm, which did not influence the simulation results. The electromagnetic induction coil heats metal porous media by generating Joule heat through eddy currents. The pyrolyzed volatiles enter the reactor from the inlet and are heated and decomposed through metal porous media to generate hydrogen (H_2) and carbon nanotube (CNT). H_2 gas flows out of the reactor outlet and CNT are attached to the metal porous media.

Porous media feature a solid skeleton and pores. For electromagnetic induction heating of plastic pyrolysis volatiles catalytic reforming by direct numerical simulation (DNS), i.e., using the actual geometry of the porous medium, is computationally intensive and inefficient due to the complex multi-physical field coupling involved. The equivalent porous medium (EPM) model treats the entire porous medium as an equivalent continuum that satisfies the assumption of a

continuum medium, so that the existing continuum medium theory can be applied to analyze the macroscopic properties of the porous medium [4]. The EPM model can enhance the calculation efficiency, especially for complicated multi-physical field coupling numerical simulations. However, the EPM model for electromagnetic induction heating of metal porous media is still underdeveloped at present.

This study develops an EPM model for electromagnetic induction heating of metal porous media and validates the developed EPM model via DNS. Although the porous medium itself is irregular, it still shows a certain periodicity as an overall structure. A porous media skeleton with a simple cubic structure was established, as shown in Figure S4B, which has the same porosity and pore size as that of the metal porous media used in this study. Since the size of the pore is significantly smaller than the overall height of the porous skeleton, it can be considered as an infinite height model. Based on this assumption, an electromagnetically equivalent infinite-height model can be obtained by extracting 1/2 of the cell height and using symmetry boundaries in the height direction. Symmetry boundaries can also be used in the circumferential direction, considering 1/8 of the circumference (Figure S4C).

Governing and boundary equations

In the simulation process, the effect of temperature on the physical properties of the electromagnetic field is not considered, so the electromagnetic field can be solved individually. Electromagnetic induction heating is realized by eddy currents and magnetic hysteresis on the target object. The alternating electromagnetic field is transmitted to the target object (conductor), forming eddy currents on the conductor and generating Joule heat. Hysteresis is the repeated magnetization of a material with ferromagnetism under the action of an alternating magnetic field, resulting in internal hysteresis loss and hence heat generation [5]. The hysteresis phenomenon and its thermal effects can be ignored because the eddy current heat is substantially greater than the hysteresis heat for most induction heating scenarios.

The electromagnetism in induction heating can be described by Maxwell's equations for time-harmonic fields:

$$\nabla \times \mathbf{H} = \mathbf{J} \tag{S1}$$

$$\mathbf{J} = \sigma\mathbf{E} + \mathrm{j}\omega\mathbf{D} + \sigma\mathbf{v}\times\mathbf{B} + \mathbf{J}_e \tag{S2}$$

$$\mathbf{B} = \nabla \times \mathbf{A} \tag{S3}$$

$$\mathbf{E} = -\mathrm{j}\omega\mathbf{A} \tag{S4}$$

where $\mathbf{E}$ and $\mathbf{D}$ are the electric field intensity and electric flux density, $\mathbf{B}$ and $\mathbf{H}$ are the magnetic flux density and magnetic field intensity, $\mathbf{A}$ is the magnetic vector potential that guarantees Gauss's law for magnetism, $\mathbf{J}$ is the electric current density, $\mathbf{J}_e$ is the externally generated current density, $\mathbf{v}$ is the velocity of the conductor (0 m·s^{-1}), σ is the electrical conductivity, j is the imaginary unit, and ω is the angular frequency.

Herein, the constitutive relations are given by:

$$\mathbf{D} = \epsilon\mathbf{E} \tag{S5}$$

$$\mathbf{H} = \mu\mathbf{B} \tag{S6}$$

where ϵ and μ are the electric permittivity and magnetic permeability.

The externally generated current density $\mathbf{J}_e$ can be calculated by homogenized multiturn model [6]:

$$\mathbf{J}_{\mathrm{e}} = \frac{NV_{\mathrm{coil}}}{AR_{\mathrm{coil}}}\mathbf{e}_{\mathrm{coil}} = \frac{NI_{\mathrm{coil}}}{A}\mathbf{e}_{\mathrm{coil}} \tag{S7}$$

where N, A, V_{coil}, R_{coil}, I_{coil}, and $\mathbf{e}_{\mathrm{coil}}$ represent the number of coil turns (equals to 20), coil cross-sectional area, coil voltage, coil resistance, coil current, and unit vector.

The eddy current heat source term generated by electromagnetic induction heating is determined by:

$$Q_{\mathrm{E}} = \mathbf{J}\cdot\mathbf{E} \tag{S8}$$

The continuity, momentum and energy equations in porous media are described by:

$$\nabla\cdot(\rho_{\mathrm{f}}\mathbf{u}_{\mathrm{f}}) = -R_{\mathrm{CNT}} \tag{S9}$$

$$(\mathbf{u}_{\mathrm{f}}\cdot\nabla)\mathbf{u}_{\mathrm{f}} = \nu(\nabla^2\mathbf{u}_{\mathrm{f}} + \frac{1}{3}\nabla(\nabla\cdot\mathbf{u}_{\mathrm{f}})) - \frac{1}{\rho_{\mathrm{f}}}\nabla p + \mathbf{S}_{\mathrm{por}} \tag{S10}$$

$$\rho_{\mathrm{f}}C_{p,\mathrm{f}}\mathbf{u}_{\mathrm{f}}\cdot(\nabla T_{\mathrm{f}}) = \nabla\cdot(\lambda_{\mathrm{f,e}}\nabla T_{\mathrm{f}}) + Q_{\mathrm{f,LTNE}} + Q_{\mathrm{react}} \tag{S11}$$

$$\nabla\cdot(\lambda_{\mathrm{s,e}}\nabla T_{\mathrm{s}}) + Q_{\mathrm{s,LTNE}} + \nabla\cdot\mathbf{q}_{\mathrm{r}} + Q_{\mathrm{E}} = 0 \tag{S12}$$

where ϕ is the porosity, ρ_{f} is the fluid density, $\mathbf{u}_{\mathrm{f}}$ is the fluid velocity, R_{CNT} is the CNT generation rate, ν is the fluid kinematic viscosity, p is the fluid pressure, $\mathbf{S}_{\mathrm{por}}$ is the additional momentum source of the porous skeleton to the fluid, $C_{p,\mathrm{f}}$ is the fluid specific heat capacity, T_{f} and T_{s} are the fluid and solid temperatures, $\lambda_{\mathrm{f,e}}$ and $\lambda_{\mathrm{s,e}}$ are the fluid and solid equivalent thermal conductivities ($\lambda_{\mathrm{f,e}} = \phi\lambda_{\mathrm{f}}$ and $\lambda_{\mathrm{s,e}} = (1-\phi)\lambda_{\mathrm{s}}$), $Q_{\mathrm{f,LTNE}}$ and $Q_{\mathrm{s,LTNE}}$ are the heat exchange between fluid and solid phases using the local thermal non-equilibrium model (LTNE), Q_{react} is the fluid phase reaction heat source term, and $\mathbf{q}_{\mathrm{r}}$ is the radiation heat flux.

$\mathbf{S}_{\mathrm{por}}$ is determined by Darcy-Forchheimer law, adopting Wu model [7]:

$$\mathbf{S}_{\mathrm{por}} = -\frac{1039-1002\phi}{d_{\mathrm{p}}^2}\nu\mathbf{u}_{\mathrm{f}} - \frac{0.5138\phi^{-5.739}}{d_{\mathrm{p}}}|\mathbf{u}_{\mathrm{f}}|\mathbf{u}_{\mathrm{f}} \tag{S13}$$

where d_{p} is the pore diameter, which is calculated by $d_{\mathrm{p}} = 0.028(\mathrm{PPI})^{-0.721}\ \mathrm{mm}$ (PPI: pore per inch) [8].

$Q_{\mathrm{f,LTNE}}$ and $Q_{\mathrm{s,LTNE}}$ are calculated by:

$$Q_{\mathrm{f,LTNE}} = -Q_{\mathrm{s,LTNE}} = h_{\mathrm{sv}}(T_{\mathrm{s}} - T_{\mathrm{f}}) \tag{S14}$$

where h_{sv} is the fluid-solid heat transfer coefficient, which is calculated by Vafai model [9]:

$$h_{\mathrm{sv}} = \frac{6(1-\phi)\lambda_{\mathrm{f}}(2+1.1Pr^{1/3}+Re^{0.6})}{d_{\mathrm{p}}^2} \quad \text{(S15)}$$

$\lambda_{\mathrm{f,e}}$ and $\lambda_{\mathrm{s,e}}$ are calculated by [10]:

$$\lambda_{\mathrm{f,e}} = \phi\lambda_{\mathrm{f}} \quad \text{(S16)}$$

$$\lambda_{\mathrm{s,e}} = \frac{(1-\phi)\lambda_{\mathrm{s}}}{3} \quad \text{(S17)}$$

where λ_{f} and λ_{s} are the fluid and solid thermal conductivities.

The porous medium in this study has a large optical thickness. The radiative transfer inside the porous medium can be considered as an isotropic radiation absorbing-scattering medium, which can be determined by the Rosseland approximation [11]:

$$\mathbf{q}_{\mathrm{r}} = \frac{16n^2\sigma_{\mathrm{SB}}T_{\mathrm{s}}^3}{3(\kappa_{\mathrm{a}}+\kappa_{\mathrm{s}})}\nabla T_{\mathrm{s}} \quad \text{(S18)}$$

where n is the refractive index (equals to 1), σ_{SB} is the Stefan-Boltzmann constant, κ_{a} and κ_{s} are the absorption and scattering coefficients of porous media, which are given by [10]:

$$\kappa_{\mathrm{a}} = \frac{3\varepsilon_{\mathrm{por}}(1-\phi)}{2d_{\mathrm{p}}} \quad \text{(S19)}$$

$$\kappa_{\mathrm{s}} = \frac{3(2-\varepsilon_{\mathrm{por}})(1-\phi)}{2d_{\mathrm{p}}} \quad \text{(S20)}$$

where $\varepsilon_{\mathrm{por}}$ is the surface emissivity of porous skeleton.

It is also necessary to consider the heat flow to the external surface at the boundary of the porous medium, which is predominantly the radiation transfer. For simplicity of calculation, the upper ($\mathbf{q}_{\mathrm{u}}$) and down boundaries ($\mathbf{q}_{\mathrm{d}}$) of the porous medium are regarded as radiation heat transfer with the environment; and the sides ($\mathbf{q}_{\mathrm{p\text{->}w}}$ and $\mathbf{q}_{\mathrm{w\text{->}p}}$) are surface-to-surface radiation heat transfer with the glass tube inner wall. $\mathbf{q}_{\mathrm{u}}$, $\mathbf{q}_{\mathrm{d}}$, $\mathbf{q}_{\mathrm{p\text{->}w}}$, and $\mathbf{q}_{\mathrm{w\text{->}p}}$ are determined by:

$$\mathbf{q}_{\mathrm{u}} = -(\varepsilon_{\mathrm{por}}\sigma_{\mathrm{SB}}T_{\mathrm{s,u}}^4)\hat{\mathbf{e}} \quad \text{(S21)}$$

$$\mathbf{q}_{\mathrm{d}} = -(\varepsilon_{\mathrm{por}}\sigma_{\mathrm{SB}}T_{\mathrm{s,b}}^4)\hat{\mathbf{e}} \quad \text{(S22)}$$

$$\mathbf{q}_{\mathrm{p\text{->}w}} = [(1-\alpha_{\mathrm{wall}})J_2 - J_1]\hat{\mathbf{e}} \quad \text{(S23)}$$

$$\mathbf{q}_{\mathrm{w\text{->}p}} = (J_1 - J_2)\hat{\mathbf{e}} \quad \text{(S24)}$$

where $\hat{\mathbf{e}}$, $T_{\mathrm{s,u}}$, $T_{\mathrm{s,d}}$, α_{wall}, J_1, and J_2 are the normal vector, upper boundary temperature, down boundary temperature, wall absorptivity, radiosity from the glass tube inner wall to the porous medium side wall, and radiosity from the porous medium side wall to the glass tube inner wall. J_1 and J_2 are calculated by:

$$J_1 = \varepsilon_{\mathrm{wall}}\sigma_{\mathrm{SB}}T_{\mathrm{wall}}^4 + (1-\varepsilon_{\mathrm{wall}}-\alpha_{\mathrm{wall}})J_2 \quad \text{(S25)}$$

$$J_2 = \varepsilon_{\text{por}} \sigma_{\text{SB}} T_{\text{s}}^4 + (1-\varepsilon_{\text{por}}) J_1 \tag{S26}$$

where $\varepsilon_{\text{wall}}$ and T_{wall} are the glass tube inner wall emissivity and temperature.

The transportation of reactive species is described by:

$$\nabla \cdot (-\rho_{\text{f}} D_{\text{df}}^i \nabla \xi_i + \rho_{\text{f}} \xi_i D_{\text{df}}^i \frac{\nabla M_n}{M_n}) + \rho_{\text{f}} (\mathbf{u}_{\text{f}} \cdot \nabla) \xi_i = R_i \tag{S27}$$

where D_{df}^i, ξ_i, M_n, R_i represent the diffusion coefficient of species i, mass fraction of species i, mean Molecular weight, and reaction rate of species i.

The predominant polyolefin pyrolysis products were alkanes with equivalent molecular formulae of $C_{20.7}H_{42.6}$–$C_{23.2}H_{46.8}$ [12]. Here, the plastic waste pyrolysis products are simplified to $C_{2m}H_{4m}$. Reactions R1–R3 are formulated using a lumped-component strategy, in which hydrocarbon species with an effective H/C ratio of 2:1 are treated as representative reactants. In particular, when m =1, reactions R1–R3 represent the reaction pathways of C_2H_4. The catalytic reforming of pyrolysis products can be expressed by [13][14][15]:

$$\text{R1: } \text{C}_{2\text{m}}\text{H}_{4\text{m}} \rightarrow 2\text{mC+2mH}_2 \tag{S28}$$

$$\text{R2: } \text{C}_{2\text{m}}\text{H}_{4\text{m}}\text{+mH}_2 \rightarrow \text{mC}_2\text{H}_6 \tag{S29}$$

$$\text{R3: } \text{C}_{2\text{m}}\text{H}_{4\text{m}}\text{+2mH}_2 \rightarrow 2\text{mCH}_4 \tag{S30}$$

$$\text{R4: } \text{C}_2\text{H}_6 \rightarrow \text{C}_2\text{H}_4\text{+H}_2 \tag{S31}$$

$$\text{R5: } \text{CH}_4 \rightarrow \text{C+2H}_2 \tag{S32}$$

The reaction rates of R1–R3 are given by [13]:

$$r_i = \frac{\text{d}c[\text{C}_{2\text{m}}\text{H}_{4\text{m}}]}{\text{d}t} = a_{\text{s}} \cdot A_i \exp(-\frac{E_i}{RT_{\text{f}}}) \cdot p[\text{C}_{2\text{m}}\text{H}_{4\text{m}}]^{n_i} \tag{S33}$$

where a_{s}, p, R, A, E, n are the specific surface area of the porous catalyst, gas partial pressure (bar), universal gas constant, pre-exponential factor, activation energy, and reaction order, as listed in Table S1.

The specific surface area of the porous catalyst a_{s} is calculated by Inayat model [16]:

$$a_{\text{s}} = 6.49 \frac{[1-0.971(1-\phi)^{0.5}]}{d_{\text{p}}(1-\phi)^{0.5}} (1-\phi) \tag{S34}$$

The reaction rate of R4 is given by [17]:

$$r_4 = \frac{\text{d}c[\text{C}_2\text{H}_6]}{\text{d}t} = A_4 \exp(-\frac{E_4}{RT_{\text{f}}}) c[\text{C}_2\text{H}_6] \tag{S35}$$

where $A_4 = 4.6 \times 10^{13}$ (s^{-1}) and $E_4 = 272.8$ (kJ/mol).

The reaction rate of R5 is given by [18][19]:

$$r_5 = \frac{\text{d}c[\text{CH}_4]}{\text{d}t} = S_{\text{por}} \cdot k_5 \frac{p[\text{CH}_4] - p[\text{H}_2]^2 / K_{\text{e5}}}{(1 + K_{\text{H5}} \sqrt{p[\text{H}_2]})^2} \tag{S36}$$

$$k_5 = A_5 \exp(-\frac{E_5}{RT_f}) \tag{S37}$$

$$K_{e5} = 5.088\times10^5 \exp(-\frac{9.12\times10^4 \text{ (J/mol)}}{RT_f}) \text{ (bar)} \tag{S38}$$

$$K_{H5} = \exp(-22.426 + \frac{1.632\times10^5 \text{ (J/mol)}}{RT_f}) \text{ (bar)} \tag{S39}$$

where $A_5 = 9.797\times10^6 \text{ (mol}\cdot\text{m/(m}^3\cdot\text{bar}\cdot\text{s))}$ and $E_5 = 104.2 \text{ (kJ/mol)}$.

Note that the model for methane cracking described above may result in negative reaction rates, suggesting that C and H_2 would be consumed to produce methane. Since C is a solid phase and does not participate in diffusion or convection, this makes the total reaction rate for C non-negative. Therefore, the reaction rate of R5 is rewritten in the following form:

$$r_5^* = \begin{cases} r_5, & r_5 \geq 0 \\ -\min(2r_1, -r_5), & r_5 < 0 \end{cases} \tag{S40}$$

Model validation of metal porous media heated by electromagnetic induction

To the best of our knowledge, there are no numerical models for electromagnetic induction heating of metal porous media. Here, we proposed the effective electrical conductivity σ_{eff} and effective magnetic permeability μ_{eff} for metal porous media, which are expressed by:

$$\sigma_{eff} = (1-\phi)\sigma_s \tag{S41}$$

$$\mu_{r,eff} = (1-\phi)\mu_{r,s} + \phi \tag{S42}$$

$$\mu_{eff} = \mu_{r,eff}\mu_{air} = \mu_{r,eff} \cdot 4\pi\times10^{-7}\,\text{N}\cdot\text{A}^{-2} \tag{S43}$$

where σ_s, $\mu_{r,s}$, $\mu_{r,eff}$, and μ_{air} are the metal electrical conductivity, the metal relative magnetic permeability, the relative effective magnetic permeability of metal porous media, and the air magnetic permeability.

This study conducted the model validation of electromagnetic induction heating of metal porous media without chemical reactions. A FNIRSI-2C23T handheld oscilloscope was used to measure the electromagnetic induction frequency and the peak voltage in the experiment. Consequently, the coil current frequency was set to 60 kHz and the current density was set to 6.67×10^5 A/m^2 in the simulation. The pore sizes of 2mm and 5mm were chosen to investigate based on the experiments in which different PPI's of metal porous media were used.

Figure S5 compares the power density and cumulative power distributions of direct numerical simulations using different pore sizes with calculations using the solid metal and the equivalent metal porous media model proposed in the present study (Eqs. S41–S43). It can be concluded that the equivalent model calculation results are close to the direct numerical simulation results, indicating the accuracy of the equivalent metal porous media model.

Numerical simulation of electromagnetic-fluid flow-heat transfer-chemical reaction multi-physics field coupling

Figure S7A shows that the fluid temperature T_f drops when the reaction of plastic pyrolysis volatiles reforming occurs, with a maximum temperature drop of nearly 200 °C at the porous media inlet. Note that the presence or absence of a reaction introduces a change in three factors for the gas flow-heat transfer: the reaction enthalpy, the change in the gas velocity, and the change in thermophysical properties such as thermal conductivity and specific heat capacity. To determine the dominate factors responsible for the change in T_f, we conducted the sensitivity analyses of factors affecting temperature change. Figure S7B indicates that the change in gas velocity is the dominant determinant of the change in porous media inlet temperature in the reactor. However, changes in the thermophysical properties of the gas mixture due to temperature, pressure and gas components determine the average and center temperatures in the reactor. The reaction enthalpy is insignificant compared to the total heat source and other heat losses. Considering the mass flow rate of reactants in the experiment and the cracking enthalpy per unit mass of pyrolysis hydrocarbons, it was also verified that the temperature drop in the reactor is insignificantly related to the reaction enthalpy. The temperature difference between the gas-solid (T_f and T_s) is most significant at the porous media inlet, and therefore the change in gas velocity has the strongest effect on the value of the maximum temperature difference at the porous media inlet. Nevertheless, the influence of the gas velocity still contributes minimally to the overall temperature drop of the reactor.

Figure S7C confirms that the reforming reaction reached the steady state after 1.5 min. Note that the transient-state case describes the change of all gas components including the purge gas argon. While the steady-state case describes the relative gas fraction changes without considering the argon component (Figure S7A). The H_2 fraction eventually stabilized at 84 vol.%, and the pyrolysis-catalysis gas still contained 16 vol.% CH_4 that could not be completely cracked, due to the fact that the gas-phase reaction had reached reaction equilibrium.

Density functional theory (DFT) calculation

We adopted the density functional theory (DFT) to investigate the catalytic reforming mechanism of plastic waste pyrolysis products by different Fe-Ni metal catalysts, i.e., pure iron (Fe), pure nickel (Ni), and Fe-Ni alloy (Fe_5Ni_5). Methane (CH_4) was chosen as a representative reactant according to the experimental results, which showed that the variation of solid carbon (C) content is mainly due to CH_4 cracking.

DFT description

Density functional theory (DFT) method is considered as an accurate tool to calculate the fundamental properties, reaction process, thermodynamic, and dynamic stability of materials on the atomic scale [20]. Its applicability ranges from atoms, molecules and solids to nuclei and quantum and classical mechanics. The original DFT has been generalized to deal with many different situations such as spin polarized models, multi constituent systems, calculating free energy at finite temperatures, superconductors, relativistic electrons, time-dependent problems and excited state systems, bosons and molecular dynamics (MD) calculations. It is well known that the quantum mechanical wave function can provide all the information about the given system. For a single hydrogen atom, we can use Schrodinger Equation and can obtain the allowed energy state of a single H atom. However, the main dilemma appears for solving Schrodinger Equation for a given N-body system. Therefore, it is necessary to involve some approximations to obtain the desired properties of a given N-body system. DFT is a method for

obtaining an approximate solution to Schrodinger Equation for N-body system. It is primarily a theory of electronic ground state structure in terms of electronic density distribution n(r). The time dependent Schrodinger Equation is given as:

$$\hat{H}\Psi_i\left(\vec{x}_1,\vec{x}_2,\vec{x}_3,.....\vec{x}_N,\vec{R}_1,\vec{R}_2,\vec{R}_3,.....\vec{R}_M\right)=E_i\Psi_i\left(\vec{x}_1,\vec{x}_2,\vec{x}_3,.....\vec{x}_N,\vec{R}_1,\vec{R}_2,\vec{R}_3,.....\vec{R}_M\right) \quad \text{(S44)}$$

where H is the Hamiltonian for a system consisting of M nuclei and N atoms, calculated by:

$$H=-\frac{1}{2m_e}\sum_{i=1}^{N}\nabla_i^2-\frac{1}{2m_n}\sum_{i=1}^{M}\nabla_j^2+\frac{1}{2}\sum_{i=1}^{N}\sum_{k=1,i\neq k}^{N}\frac{e^2}{\left|r_i-r_k\right|}-\sum_{i=1}^{N}\sum_{j=1}^{M}\frac{z_j e^2}{\left|r_i-r_k\right|}+\frac{1}{2}\sum_{j=1}^{N}\sum_{w=1,w\neq j}^{N}\frac{z_j z_w e^2}{\left|r_j-r_w\right|} \quad \text{(S45)}$$

$$H=T_e+T_n+V_{ee}+V_{en}+V_{nn} \quad \text{(S46)}$$

The first two terms in Eq. S46 represent the kinetic energy of electrons T_e and nuclei T_n; the third term is electron-electron potential energy V_{ee}; the fourth term is potential energy between electron and nuclei V_{en}; and the last term is the nuclei-nuclei potential energy V_{nn}. A detailed introduction about DFT method is given in Ref. [20].

First principles-DFT calculations were performed to analyze CH_4 direct dissociation on Fe, Ni, and Fe_5Ni_5 alloy on their inherent surface states. These calculations were conducted within the Generalized Gradient Approximation (GGA) framework, using the Perdew-Burke-Ernzerhof (PBE) exchange-correlation (XC) functional [21], which is renowned for its efficient convergence in cluster [22] and surface-based calculations [23]. The Vienna *ab-initio* Simulation Package (VASP) was employed, adopting the projector-augmented-wave (PAW) formalism. Additionally, the D3 dispersion correction (PBE-D3), developed by Grimme, was included to account for van der Waals interactions [24].

To obtain accurate convergence and optimized geometries of CH_4, Fe, Ni, and Fe_5Ni_5 systems, the energy convergence values was set to 1×10^{-6} eV/atom, and the forces per atom were fixed at 0.01 eV/Å. The energy cut-off value was applied at 500 eV, and a Monkhorst-Pack grid of 13×13×1 k-points was used for Brillouin zone sampling. A vacuum thickness of 20 Å was applied along the Z-direction to prevent interlayer interactions. All calculations were performed on 3×2×1 supercell configuration of the Fe, Ni, and Fe_5Ni_5 alloy systems.

Molecular structure

In order to determine the CH_4 dissociation on Fe, Ni, and Fe_5Ni_5 alloy, one CH_4 molecule was adsorbed on mentioned materials as shown in Figure S11, respectively, marked as initial geometries. As illustrated in Figure S11, a 3×2×1 supercell for each Fe, Ni, and Fe_5Ni_5 geometry was modelled; and one CH_4 molecule was placed on atop site of given materials. The CH_4 molecule was placed in such a manner that C atom's center weight was facing the surface of base material. The Fe_5Ni_5 alloy's ratio was maintained by proportionating the atoms of Fe and Ni as per given ratio of atoms for the Fe_5Ni_5 alloy. Later on, all the structures were fully relaxed until the energy and force convergence criterion was not achieved.

The CH_4 dissociation and the respective reaction intermediates on catalytic surfaces were explored after obtaining the optimized structures of CH_4 adsorbed on the Fe, Ni, and Fe_5Ni_5 alloy. Consequent dissociation of CH_4 into C and H_2 entities was thoroughly studied. The Gibbs Free Energy diagram was calculated for the elementary reactions involved in CH_4 dissociation to sufficiently understand the kinetics of CH_4 dissociation. During the cracking of CH_4 into C and

H_2, the transition from CH_4 to C and H_2 involved multiple steps as follows:

Step 1: $CH_4 \rightarrow CH_3^* + H^*$
Step 2: $CH_3^* + H^* \rightarrow CH_2^* + H_2$
Step 3: $CH_2^* + 2H^* \rightarrow CH^* + 3H^*$
Step 4: $CH^* + 3H^* \rightarrow C + 2H_2$

The Gibbs free energy (G) for each state along the reaction coordinate can be calculated using the equation given below [25]:

$$\Delta G = E_{Total} + ZPE + K_B T \ln Q - TS_{vib} \quad \text{(S47)}$$

where E_{Total} is the total energy of the given system, i.e., CH_4 loaded catalytic system, ZPE defines the zero-point energy, $K_B$is the is the Boltzmann constant, T is the standard temperature, Q refers to the partition function, and S_{vib} is the vibrational entropy, obtained from the vibrational frequencies of given system.

The CH_4 dissociation reaction pathway is shown along the reaction coordinate, with various transition states (TSs) and intermediates (IMs) of TS_1, IM_1, TS_2, IM_2, TS_3, and IM_3. Table S4 lists the activation energies for the elementary reactions on the catalytic surfaces. Moreover, the TSs energies were calculated through CI-NEB technique in Figure 2D [26]. The total energy of CH_4 molecule and the catalytic surface was taken as the energy reference of the diagram. As seen through the activation energy E_a in Table S4 and the Gibbs free energy values ΔG in Figure 2D, it can be seen that the CH_4 dissociation on pure Ni and Fe is energetically more favorable in comparison to the composite alloy of Fe_5Ni_5, postulating that the comparative higher C and H yield will be obtained on these catalytic surfaces.

We have compared our DFT calculation results with existing literature to ensure that the model is not treated in isolation. Our results show that the activation energy barriers for the stepwise dehydrogenation of methane on single-metal Ni surfaces fall well within the ranges reported in numerous previous studies [27]. In addition, our calculations indicate that the Fe-Ni alloy exhibits higher activation barriers than the corresponding pure Ni and Fe surfaces, which is consistent with literature reports on alloy-induced electronic effects [28–29].

By examining the activation energies of individual elementary steps, we further find that the first and fourth steps of methane cracking are kinetically more demanding, in agreement with prior mechanistic analyses [30]. Quantitative differences in reported activation energies among studies mainly arise from variations in surface models (e.g., Ni(111) vs. Ni(100)), alloy compositions (e.g., CuNi, NiCo), electronic effects, and computational settings [27,28,30]. The relatively larger deviations observed for Fe and Fe_5Ni_5 surfaces in our work can be attributed to the strong facet sensitivity of Fe surfaces and the explicit treatment of alloy-induced electronic modulation, whereas many earlier studies employed idealized Fe facets or simplified alloy models [28,30]. Although absolute activation energies may differ, all calculated barriers remain within reported literature ranges and consistently reproduce the same mechanistic trends.

Through CI-NEB technique implemented in VTST toolkit [26], we studied the transition and intermediate geometries for CH_4 dissociation on Fe, Ni, and Fe_5Ni_5 alloy. The initial geometry was modelled as the stable CH_4 molecule adsorbed on the surface of the catalysts mentioned

above, and the final geometry was modelled as C and $2H_2$ entities available on the surface of given catalysts. Through Gibbs free energy diagram, that pure Ni and Fe catalytic surfaces provided lower energy barriers and effective kinetics for CH_4 dissociation process in comparison to Fe_5Ni_5 alloy. Corresponding TSs structures of the dissociation reactions on the catalytic surfaces in Figure S12 agreed well with earlier worked upon transition metal surfaces [29][31][32]. Through the TSs and IMs structures provided in Figure S12, CH_4 molecule sits atop of Ni or Fe atoms during pure and alloy configurations. However, as observed through the IM_1, the CH_3^* fragment still maintained its atop position and the dissociated H atom attempted to adsorb near the bridge site. Furthermore, the dissociated C atom of CH_4 made bonding with Fe atom, thereby getting chemically adsorbed. However, the dissociated C atom was physically adsorbed by Ni atom.

After analyzing the relaxed initial and final structures, Gibbs free energy and TSs structures of various CH_4 adsorption pure metals and composite alloy, we investigated the partial density of state (PDOS) diagrams for all given systems and are presented in Figure S13. The PDOS diagrams were generated for all the initial structures of CH_4 adsorption on Fe, Ni, and Fe_5Ni_5 catalytic surfaces, so as to confirm the CH_4 adsorption capability of given systems. As observed in Figure S13 that, the orbitals of C atom, 3d TM atoms and H atoms show overlapping peaks at -10 to -5 eV energy range, i.e., deep valence bands, thus suggesting a suitable hybridization behavior between adsorbate catalyst surface and the adsorbent CH_4 molecule. This indicates that initially CH_4 can be easily adsorbed on these catalytic surfaces. Furthermore, if the we observe the d orbitals of Ni and Fe atoms in between -2 to 2 eV energy range, the d orbital peaks of Fe and Ni lie in same energy range, which further indicates that the stable Fe and Ni alloys can be developed with varying composition of Fe and Ni atoms, respectively. Furthermore, C atom p orbitals and H atom s orbitals show multiple peaks in valence bands, these multiple hybridizing peaks illustrate that CH_4 can be tightly adsorbed on these pure metal and composite alloy. The stronger hybridization between CH_4 and catalytic surfaces indicates that external stimuli such as higher temperatures will be required to dissociate CH_4 into and C and H_2 entities, respectively.

Techno-economic analysis (TEA)

Process simulation

The TEA is explicitly based on experimentally measured H_2 and CNT yields obtained in this work, rather than on idealized assumptions or literature-derived values. This ensures that the economic evaluation accurately reflects the actual performance and technical limitations of the proposed induction-heated pyrolysis-reforming system. The analysis focuses on key economic metrics relevant to this process, namely the H_2 production cost and the sensitivity of overall economics to the CNT selling price, following methodologies widely adopted in comparable techno-economic studies [33–36].

A system for producing CNT from plastic waste is proposed, based on the investigation of induction-heated reforming in this study. The proposed system, illustrated in Figure S35, includes a pyrolizer, an induction-heated reformer, a water-gas shift (WGS) module, and a gas separation unit utilizing Pressure Swing Adsorption (PSA) technology. In this system, H_2 is also produced as a primary product alongside CNT. Additionally, CO_2 is separated downstream in the process. A process model of the proposed system was developed using Aspen Plus to conduct heat and mass balance calculations. The Peng-Robinson (PR) property method was employed to

assess the properties of the hydrocarbon streams in the process model. The conceptual design and modeling assumptions for each process step are outlined below.

Feedstock

The plastic feedstock used for the process simulation consists of WPE and WPP samples, with their ultimate and proximate analyses detailed in Table S5. Based on their ultimate analysis, the higher heating value (HHV) of these samples was estimated using the correlation proposed by Channiwala and Parikh (2002) [37]. This resulted in HHVs of 43.6 MJ/kg for the WPE sample and 35.1 MJ/kg for the WPP sample. In the process simulation, the plant is designed to process 1000 kg of plastic per hour, which corresponds to approximately 9.8–12.1 MWh of HHV, depending on the type of plastic waste.

Pyrolysis

The pyrolysis process is assumed to be conducted using an auger reactor, a well-established technology for pyrolysis. In this process simulation, the auger reactor is assumed to be heated via Joule heating, using a screw made from electrically conductive materials inside the reactor. During pyrolysis, WPE and WPP are converted into solid char and a vapor fraction at 500 °C, which consists of condensable oil and permanent gases. The yield and composition of these products are based on lab-scale experimental work conducted in this study. Detailed product yields for both samples are provided in Table S12 [2][12]. The representative compounds of the condensable oil used in the process simulation were selected based on GC/MS analysis results. For simplicity, compounds with the same number of carbon atoms are represented by a single compound with that carbon number. In the process simulation, the pyrolysis is modeled using an RYield block, with electricity consumption determined by the heat duty calculated from the RYield block. For simplification, heat loss from the reactor is neglected in this study. In addition, the pyrolysis process generates a significant amount of solid residue, which may pose environmental and health risks depending on the nature of the original feedstock. Therefore, a disposal cost of 0.26 $/kg is assumed for the char reside from the pyrolysis process. This value represents a typical cost for the treatment and disposal of solid hazardous waste in China [38].

Induction-heated reformer

After the pyrolysis process, the vapor fraction from the pyrolysis reactor is fed into the reformer unit, which is heated by an induction heater. Before entering the reformer, a heat exchanger (HX-1, as shown in Figure S35) is used to raise the vapor temperature by recovering heat from the syngas exiting the reformer. In the reformer, the pyrolysis vapor is cracked into CNT and syngas, primarily composed of H_2, CO, and CH_4, as previously described in the experimental section of this study. The reforming process operates at 900 °C and is modeled using an RGibbs block, which employs the Gibbs free energy minimization method to predict product composition. In the model, the solid carbon produced is assumed to be 100% CNT. The CNT yield is based on experimental results using induction heating at 900 °C, resulting in 72.4 wt.% and 57.1 wt.% for WPE and WPP samples, respectively. Based on the continuous-feeding experimental results, approximately 60 wt.% of the generated CNT can be collected using a purge gas at 0.15 MPa. The remaining CNT can be recovered through mechanical vibration after the catalyst's operational lifespan concludes. The gas yield is determined using the Gibbs equilibrium approach.

The total electricity consumption is calculated with an assumed reactor efficiency of 90%, corresponding to the estimated efficiency of an induction-heated reactor at the proposed scale, based on the model developed by Almind et al. [39]. The reformer is equipped with a cyclone, modeled using a simple separator block, to separate CNT from the hot syngas with 100% recovery of CNT. The remaining hot syngas is cooled through a heat exchanger and cooler before being fed to the WGS section.

WGS section
The water-gas shift (WGS) conversion is modeled as a two-stage process: a high-temperature shift at 400 °C, followed by a low-temperature shift at 250 °C. In these stages, most of the CO is converted into CO_2 and H_2 through the water-gas shift reaction by reacting with H_2O. The gas exiting the high-temperature reactor is cooled before entering the low-temperature reactor. In the Aspen Plus simulation, two REquil blocks are used to represent the two conversion stages. The steam-to-carbon ratio for the shift conversion is set at 2 moles of H_2O per mole of CO. During the reaction, CH_4 is assumed to be inert, consistent with the findings of Chianese et al. [40], who observed minimal CH_4 change during the water-gas shift of tar-rich syngas from biomass gasification at similar WGS temperatures. The H_2-rich gas exiting the low-temperature reactor is then cooled through a heat exchanger (HX-2) and a cooler before being compressed for the subsequent gas separation step.

Gas separation
The gas separation process involves two adsorption stages. The first stage utilizes a Vacuum PSA (VPSA) system to separate high-purity streams of H_2 and CO_2 from the H_2-rich gas produced in the WGS process. This VPSA system aims to generate H_2 with a purity of ≥99.97% (suitable for industrial applications and fuel cells) and CO_2 with a purity exceeding 95%, both at a recovery rate of 90%. The VPSA operates with a feed pressure of 30 bar, achieved using a multi-stage compressor, and an evacuation pressure of less than 0.1 bar. The energy consumption for the VPSA unit is estimated at 600 kJ/kg_{CO2}, with detailed system configuration and performance described in other sources [41]. The offgas from the VPSA is then directed to a second separation stage, which focuses on recovering CH_4. This stage is modeled after a PSA unit developed for biogas upgrading, as reported by Augelleti et al. [42]. The VPSA offgas composition, similar to raw biogas with over 40 vol.% CH_4, is processed with a feed pressure of 6 bar and an evacuation pressure of 0.2 bar [42], with an energy consumption of 0.232 kWh/kg_{CH4}. The recovered CH_4 is recycled back into the production system as feedstock for the reformer unit, enhancing the yields of CNT and H_2. In the simulation, the compressors are assumed to have an isentropic efficiency of 85% and a mechanical efficiency of 95%, while the heat exchangers are designed with a minimum temperature difference of 10 °C.

Techno-economic calculations
The primary economic metric used is the Levelized Cost of Hydrogen (LCOH), given the growing interest in H_2 production, including through reforming processes involving alternative solid feedstocks. This allows for a comparison of the proposed induction-heated catalytic reformer with other technologies based on economic performance. The LCOH is calculated by determining the hydrogen selling price needed to reach a net present value (NPV) of zero by the end of the plant's operational lifespan, factoring in possible revenue from selling CNT at specified prices.

The fixed capital and operating costs are estimated from the simulation results, primarily using information from public scientific publications and engineering handbooks. Equipment sizes, determined from mass and energy balances, serve as the basis for estimating the costs of process equipment. For calculating capital costs at various scales, general cost correlations are applied for standard components like heat exchangers and cyclones (as outlined in [43]), while simplified scaling correlations are used for more complex units, as described in Eq. S48, where R represents the scaling factor. The breakdown of costs and scaling exponents for individual process units can be found in Tables S11–13. These cost estimates are adjusted for inflation to reflect 2024 values, using the Chemical Engineering Plant Cost Index (CEPCI), as outlined in Eq. S49.

$$\frac{Cost_a}{Cost_b} = \left(\frac{Size_a}{Size_b}\right)^R \tag{S48}$$

$$Cost_{€\ Year\ Y} = Cost_{€\ Year\ X} \cdot \frac{CEPCI_{Year\ Y}}{CEPCI_{Year\ X}} \tag{S49}$$

The Total Capital Investment (TCI) is estimated based on the equipment purchase costs, adjusted using scaling factors specific to solid/fluid processing plants, as indicated in reference [44] (with a factor of F = 4.87). A 20% contingency is added to account for the significant uncertainty in estimating the costs of the production system. The capital cost estimation accuracy using this methodology usually varies between -30% and +50 % [45]. The detailed factors used for calculating the TCI are listed in Table S13. Annualized capital costs are calculated based on the TCI, the plant's operational lifespan, and the interest rate. Additionally, annual operating and maintenance (O&M) costs are included to finalize the LCOH calculation. Table S14 outlines the primary assumptions used in the economic evaluation. Operating costs cover expenses for feedstock and utilities, revenue generated from CNT products, and variable operational costs, as detailed in Table S15.

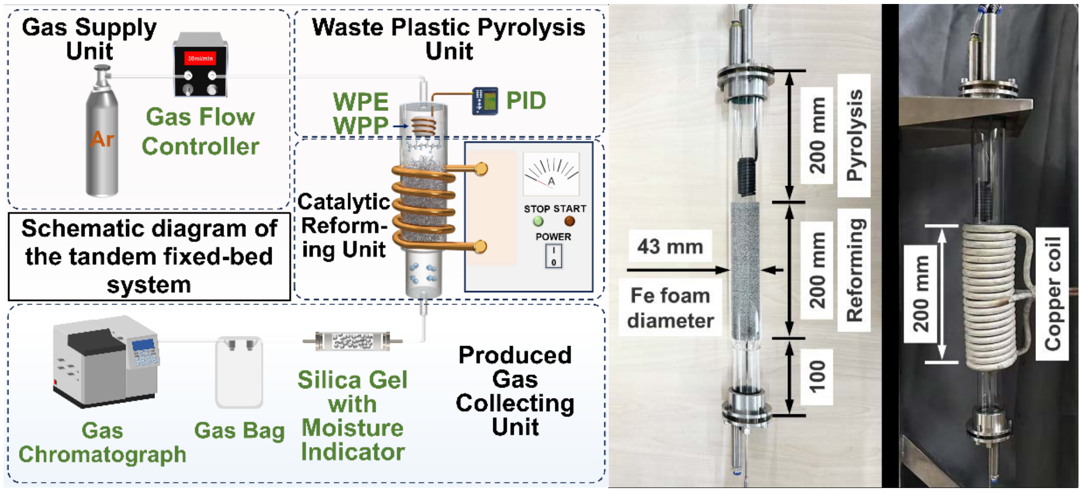

Figure S1. The schematic diagram of the tandem fixed-bed system for waste plastics pyrolysis-catalysis process.

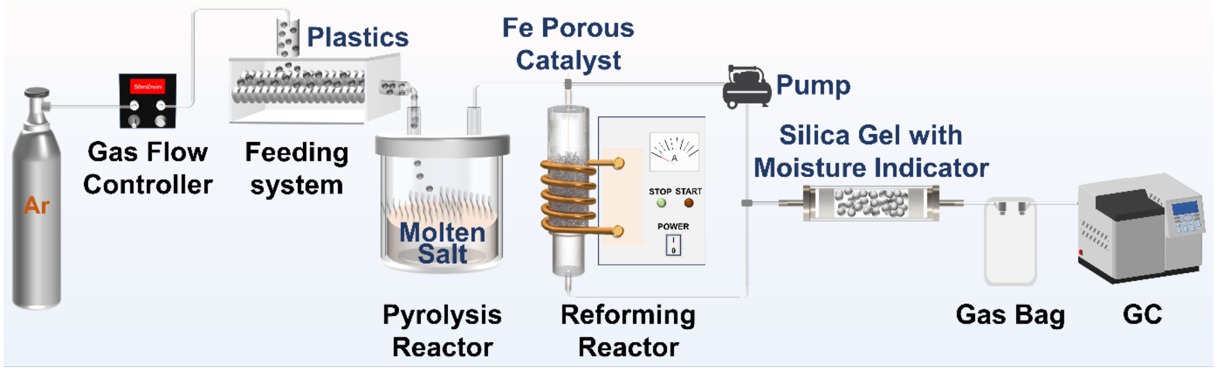

Figure S2. The schematic diagram of the pyrolysis-catalysis of plastic waste with continuous feeding.

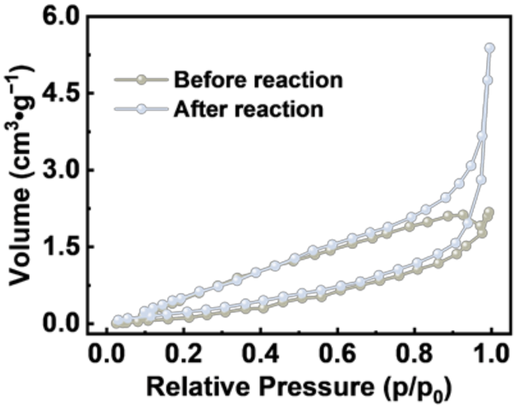

Figure S3. N_2 adsorption-desorption isotherms of Fe porous skeleton before and after reaction.

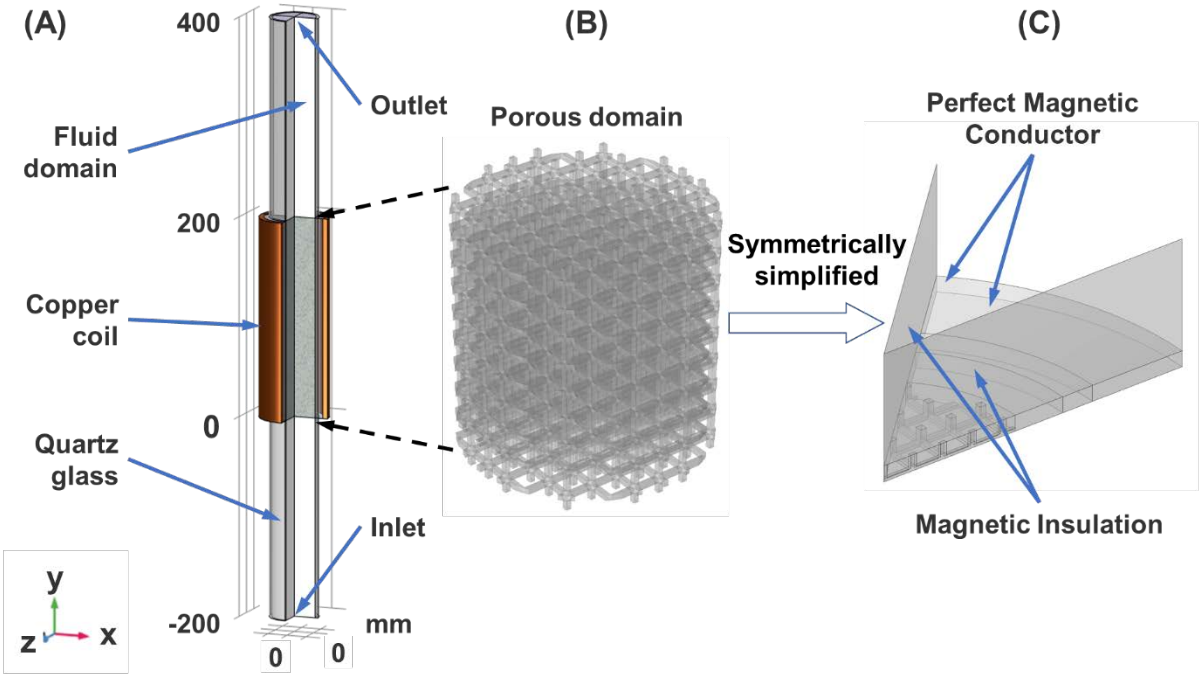

Figure S4. Physical model description for CFD simulation.

(**A**) Physical model of catalytic reforming of plastic waste pyrolysis volatiles with electromagnetic induction monolith heating. (**B**) Cubic skeleton porous media structure. (**C**) Porous media symmetry simplification.

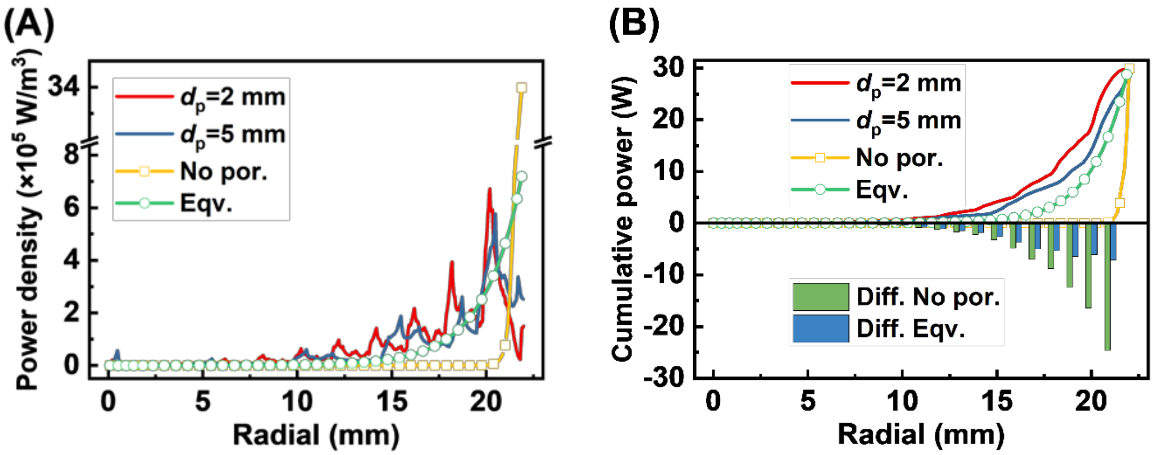

Figure S5. Simulation results of direct numerical simulations using different pore sizes with calculations using the solid metal and the equivalent metal porous media model.

(**A**) Power density and (**B**) cumulative power distributions. Diff. represents the difference between the predictions of the equivalent porous media (EPM) model and the direct numerical simulation (DNS); No por. and Eqv. represent the solid metal model and equivalent metal porous media model.

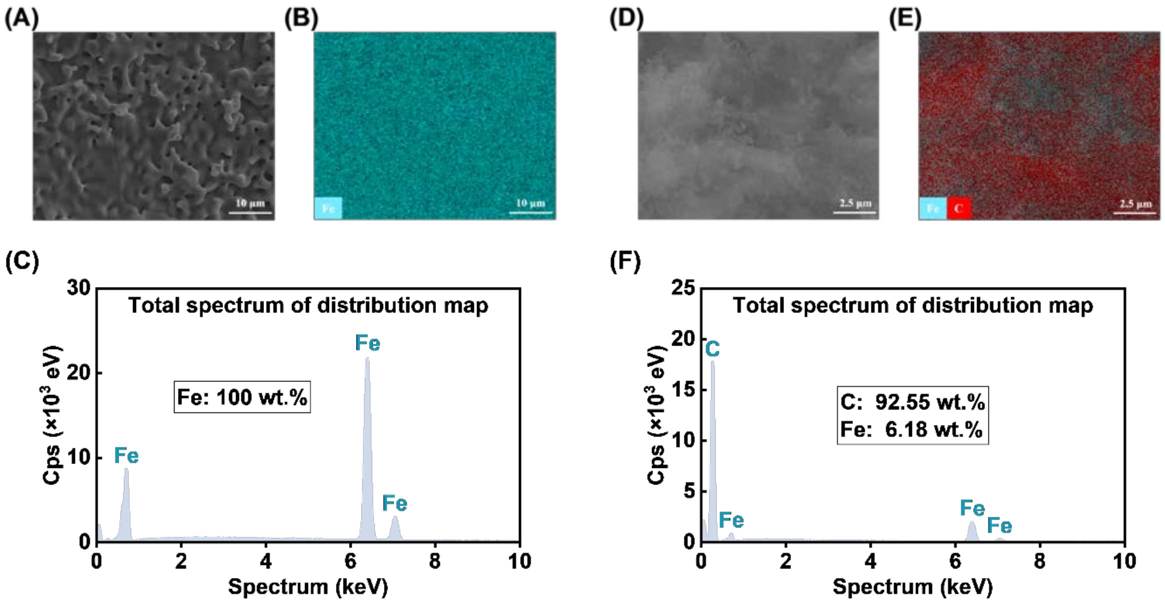

Figure S6. Surface morphology and elemental distribution of Fe porous catalyst before and after reaction.

(**A**) Surface morphology before reaction. (**B**) Surface elemental distribution before reaction. (**C**) Surface elemental quantitative analysis before reaction. (**D**) Surface morphology after reaction. (**E**) Surface elemental distribution after reaction. (**F**) Surface elemental quantitative analysis after reaction.

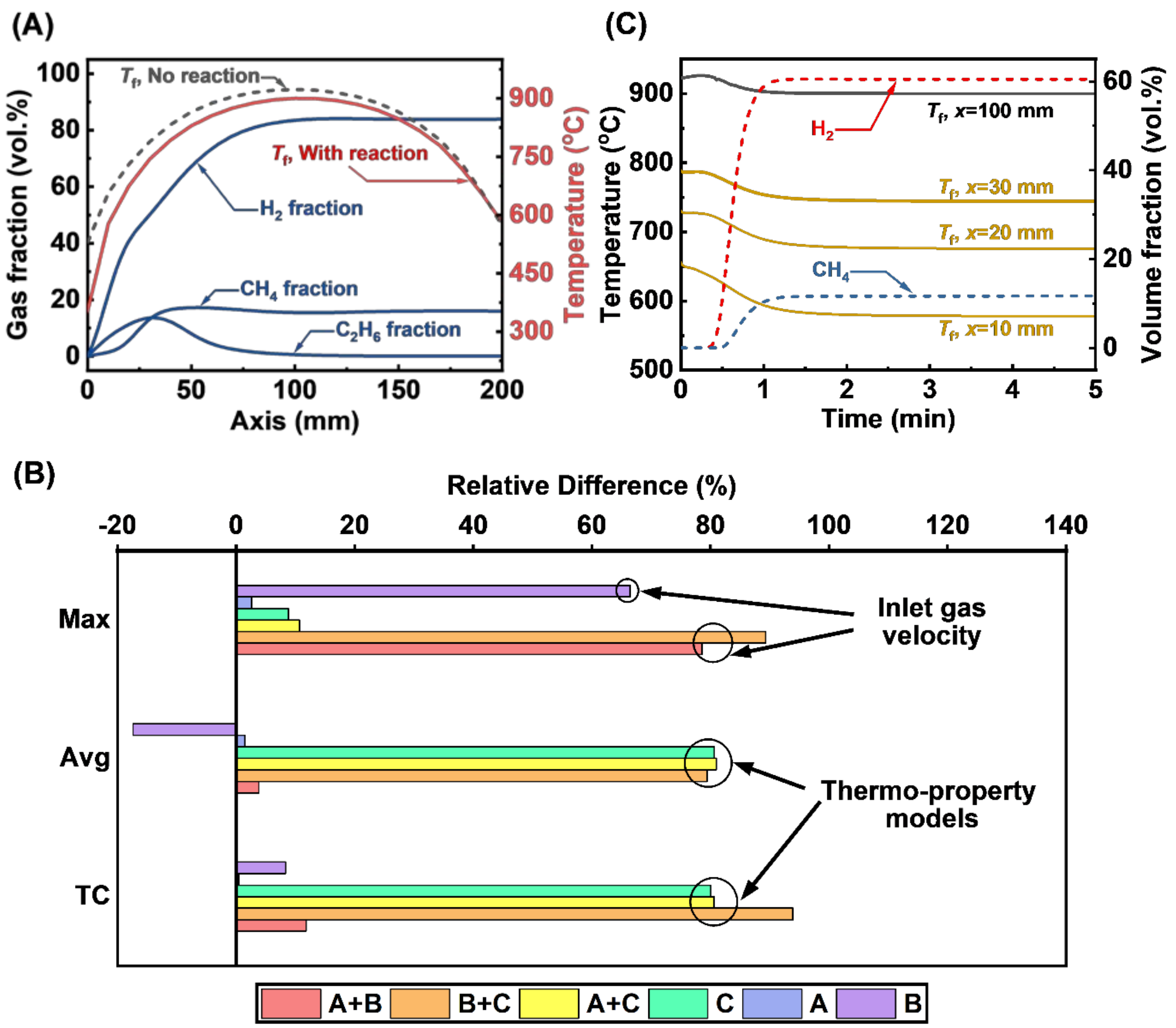

Figure S7. Numerical simulation results of plastic pyrolysis volatiles reforming driven by electromagnetic induction heating.

(**A**) Comparison of fluid temperature T_f with and without reaction at r = 0 mm, and distribution of H_2, CH_4, and C_2H_6 fractions at steady state. (**B**) Sensitivity analyses of factors affecting temperature change. (**C**) Transient T_f changes at different axial positions and changes in outlet H_2 and CH_4 fractions. Max, Avg, and TC represent the inlet, average, and center point fluid temperatures. A denotes a model that takes into account the reaction enthalpy, B denotes a model that takes into account the change in gas velocity, and C denotes a model that takes into account the change in gas thermophysical properties; A+B denotes a model that takes into account the reaction enthalpy and the change in gas velocity, B+C denotes a model that takes into account the change in gas velocity and gas thermophysical properties, A+C denotes a model that takes into account the reaction enthalpy and the change in gas thermophysical properties.

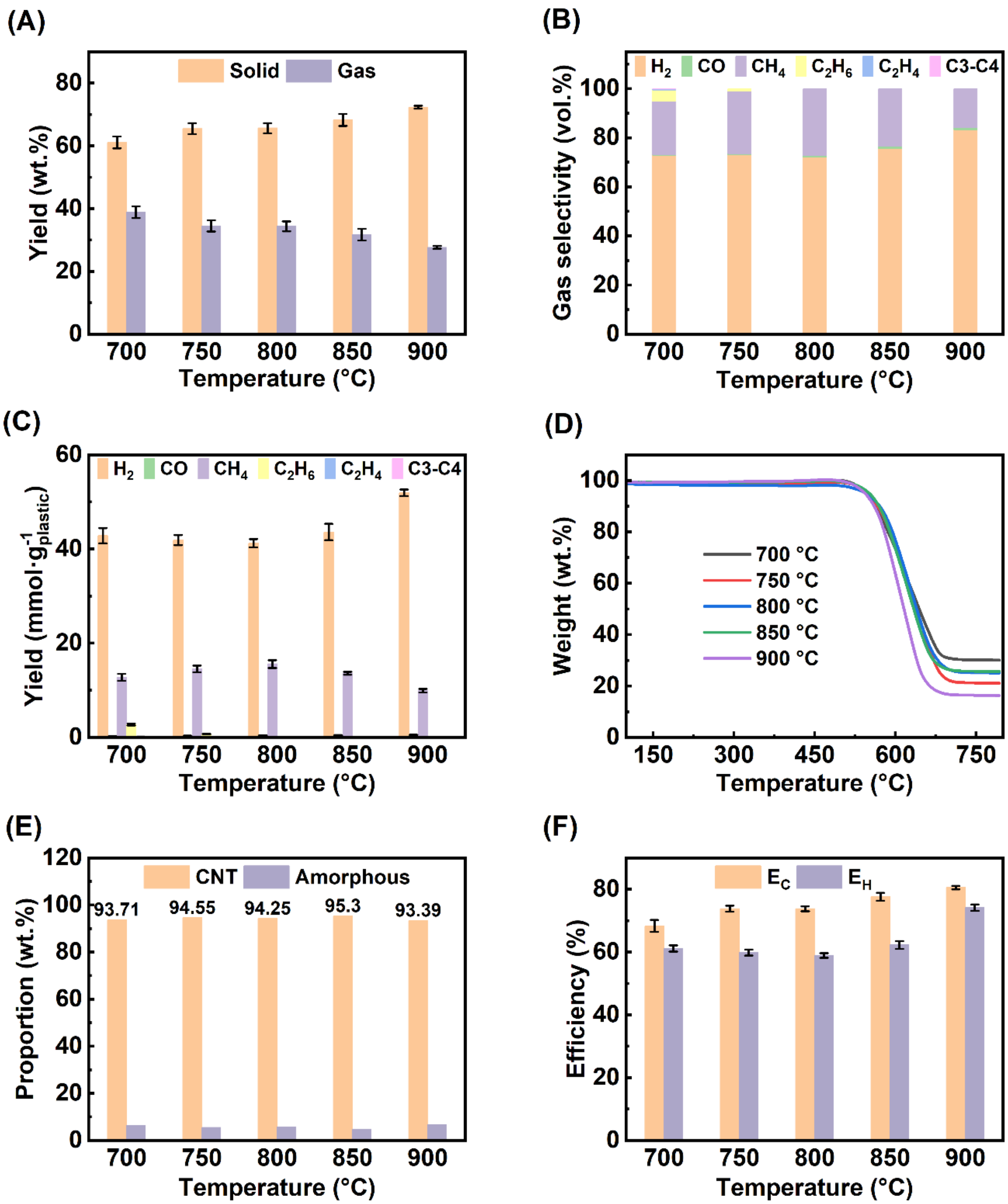

Figure S8. The effect of catalytic temperature on plastic pyrolysis-catalysis products. (**A**) Solid and gas yields at different temperatures. (**B**) Gas selectivity at different temperatures. (**C**) Specific gas yields at different temperatures. (**D**) TGA curves of the produced carbon at different temperatures. (**E**) CNT and amorphous carbon proportions of the produced carbon at different temperatures. (**F**) E_C and E_H at different temperatures. E_C and E_H represent the atom recovery efficiencies of C and H.

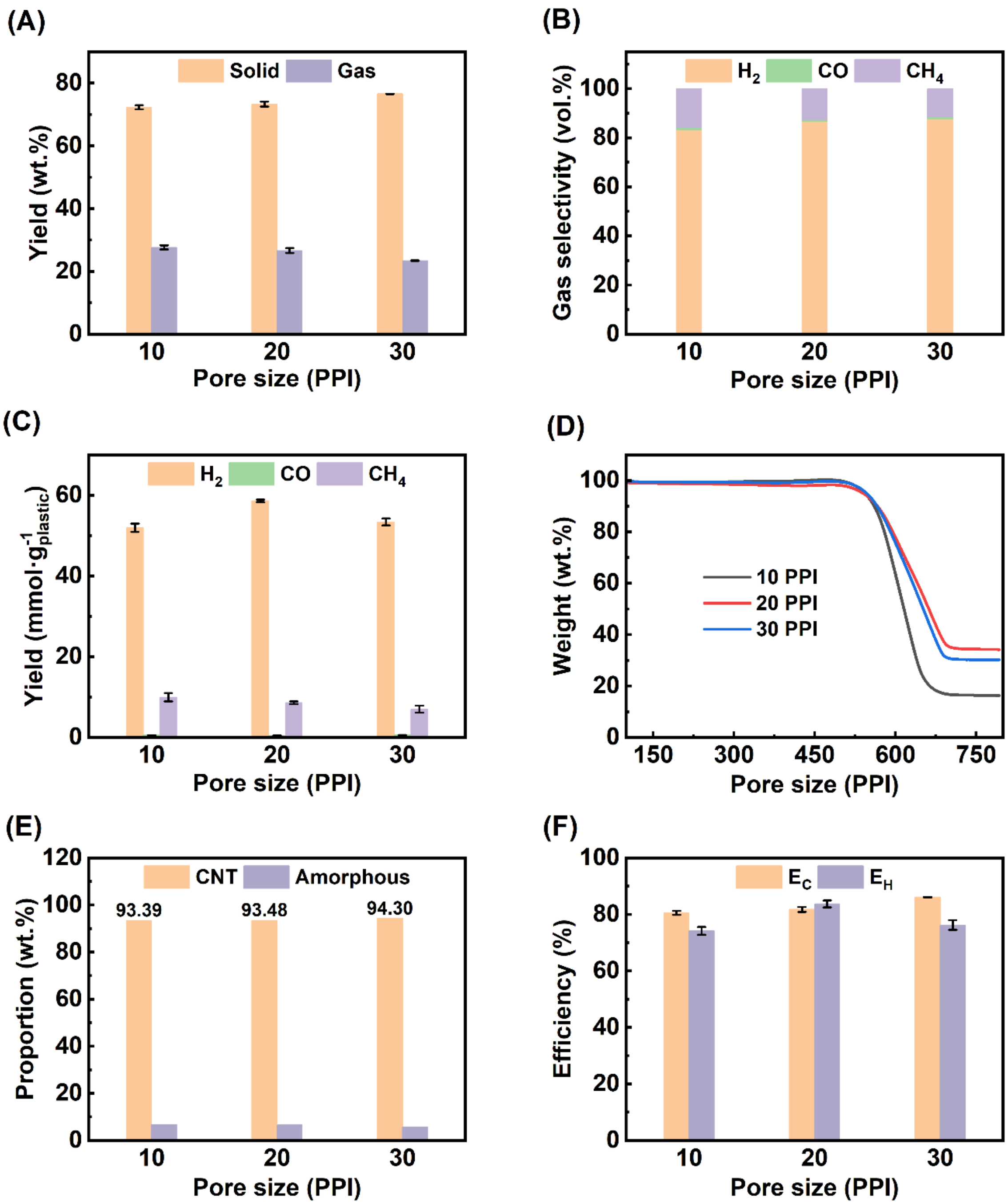

Figure S9. The effect of porous media pore size on plastic pyrolysis-catalysis products. (**A**) Solid and gas yields at different pore sizes. (**B**) Gas selectivity at different pore sizes. (**C**) Specific gas yields at different pore sizes. (**D**) TGA curves of the produced carbon at different pore sizes. (**E**) CNT and amorphous carbon proportions of the produced carbon at different pore sizes. (**F**) E_C and E_H at different pore sizes.

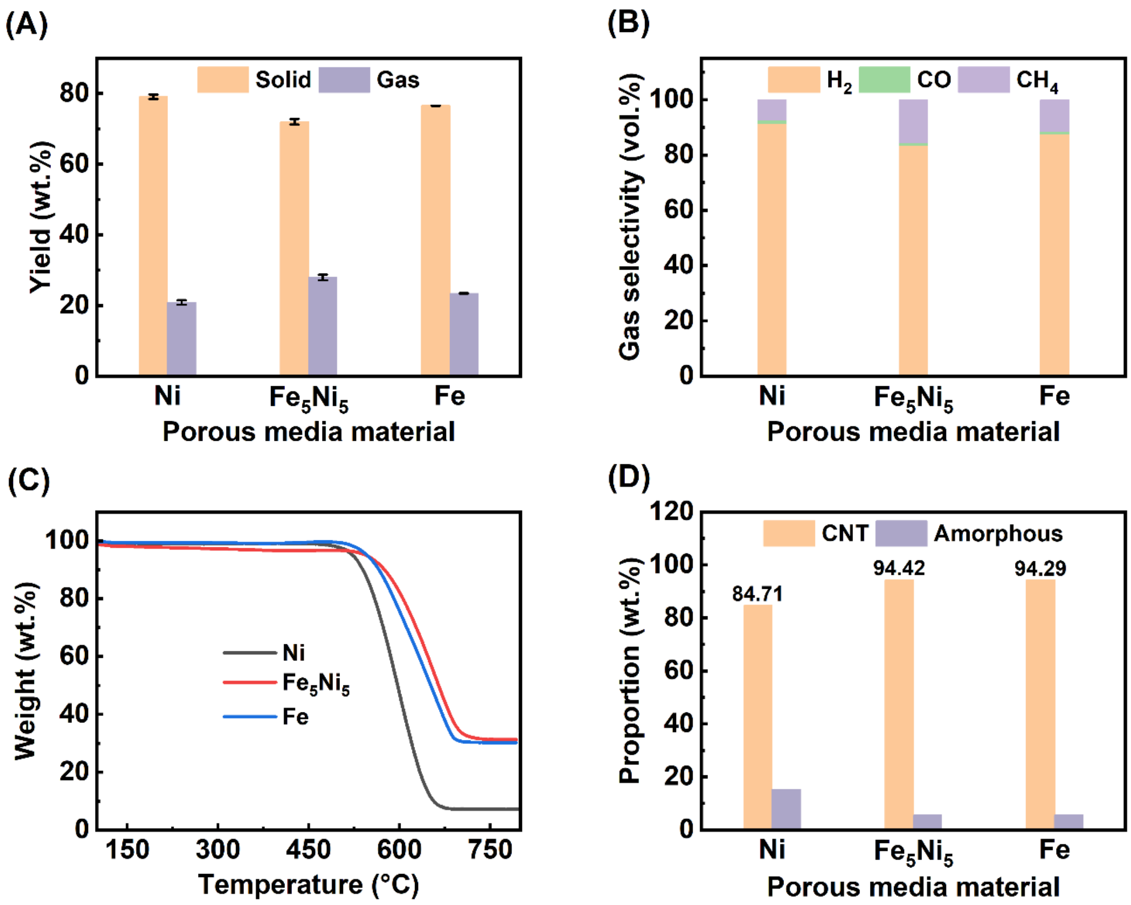

Figure S10. The effect of porous media material on plastic pyrolysis-catalysis products. (**A**) Solid and gas yields at different pore sizes. (**B**) Gas selectivity at different pore sizes. (**C**) TGA curves of the produced carbon at different pore sizes. (**D**) CNT and amorphous carbon proportions of the produced carbon at different pore sizes.

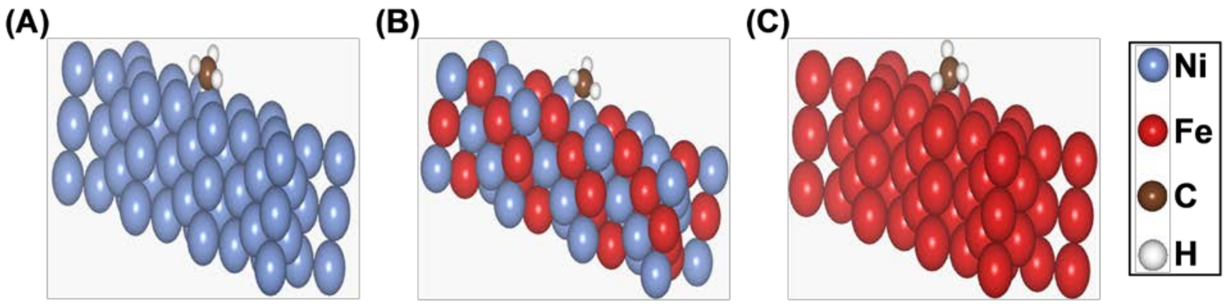

Figure S11. **Initial atomic structures of CH_4 adsorption and cracking on different catalysts.** (**A**) Ni, (**B**) Fe_5Ni_5 alloy, and (**C**) Fe.

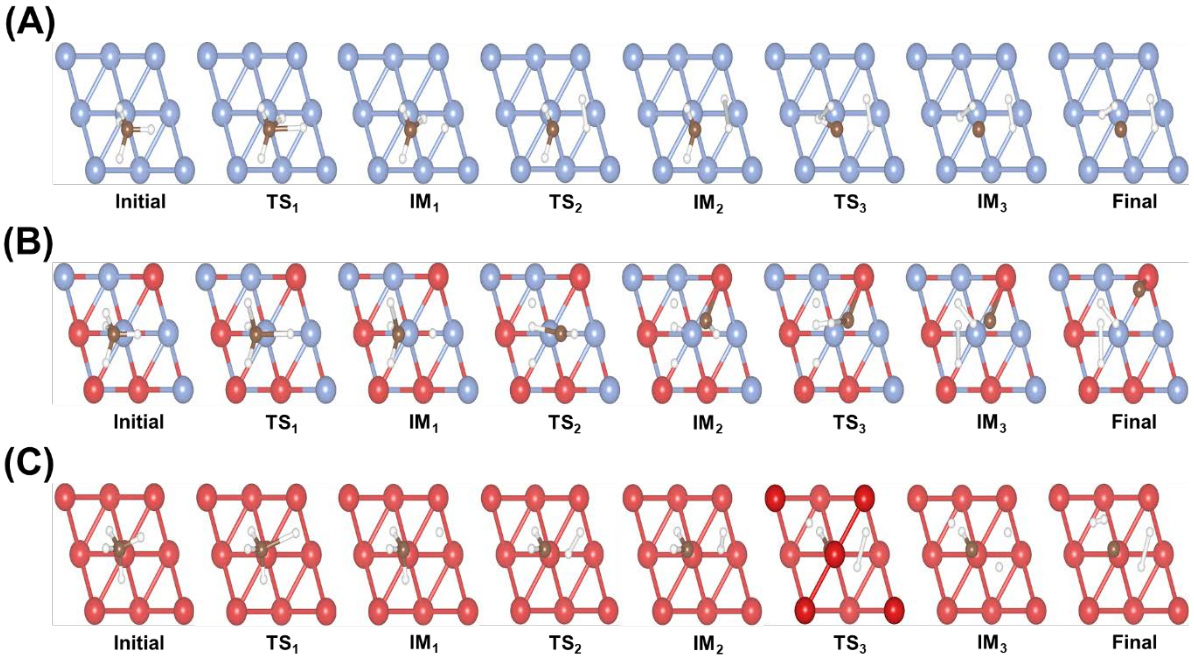

Figure S12. Illustration for the TSs (Transitions States) and IMs (Intermediate States) of CH_4 dissociation on different catalytic surfaces.

(**A**)Ni, (**B**) Fe_5Ni_5, (**C**) and Fe catalytic surfaces. Blue, red, grey and white balls represent Ni, Fe, C and H atoms, respectively.

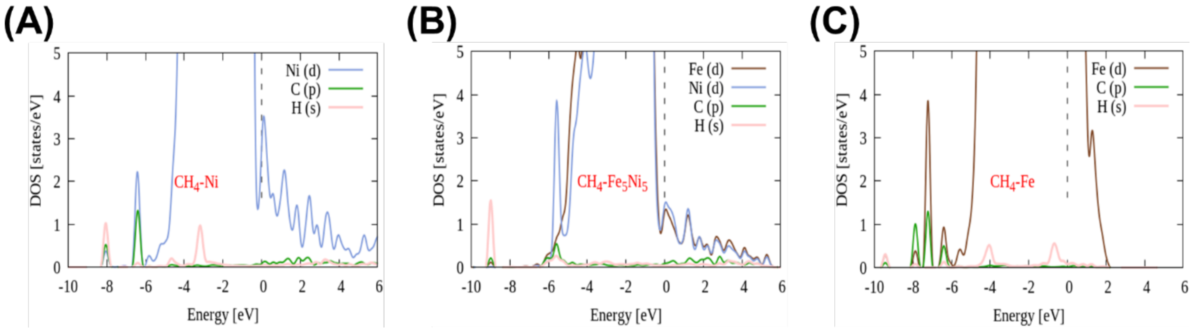

Figure S13. PDOS diagrams of CH4 adsorbed on different catalysts.
(**A**) Ni, (**B**) Fe_5Ni_5 alloy, and (**C**) Fe.

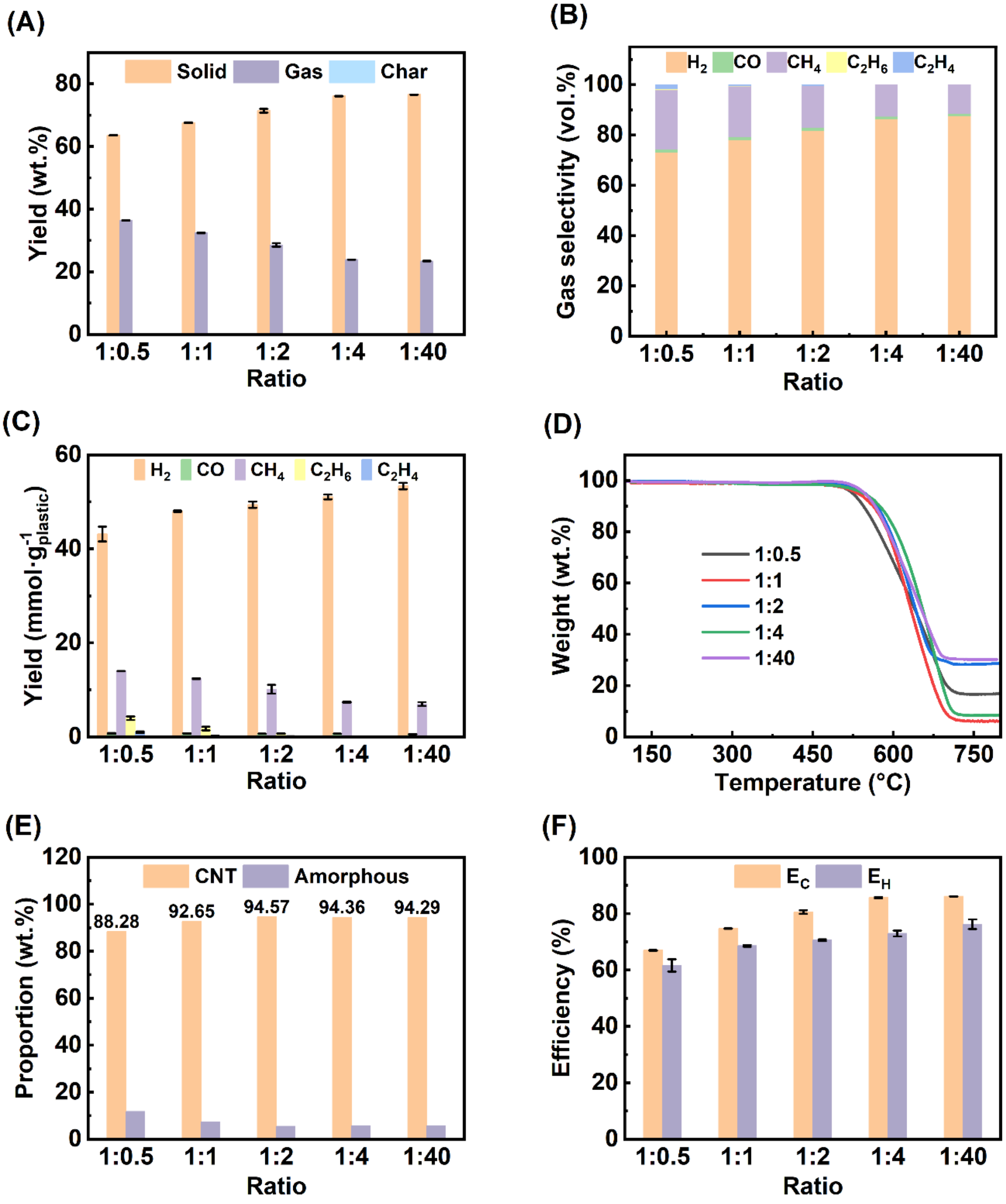

Figure S14. The effect of plastic-to-catalyst mass ratio on plastic pyrolysis-catalysis products.

(**A**) Solid and gas yields at different mass ratios. (**B**) Gas selectivity at different mass ratios. (**C**) Specific gas yields at different mass ratios. (**D**) TGA curves of the produced carbon at different mass ratios. (**E**) CNT and amorphous carbon proportions of the produced carbon at different mass ratios. (**F**) E_C and E_H at different mass ratios.

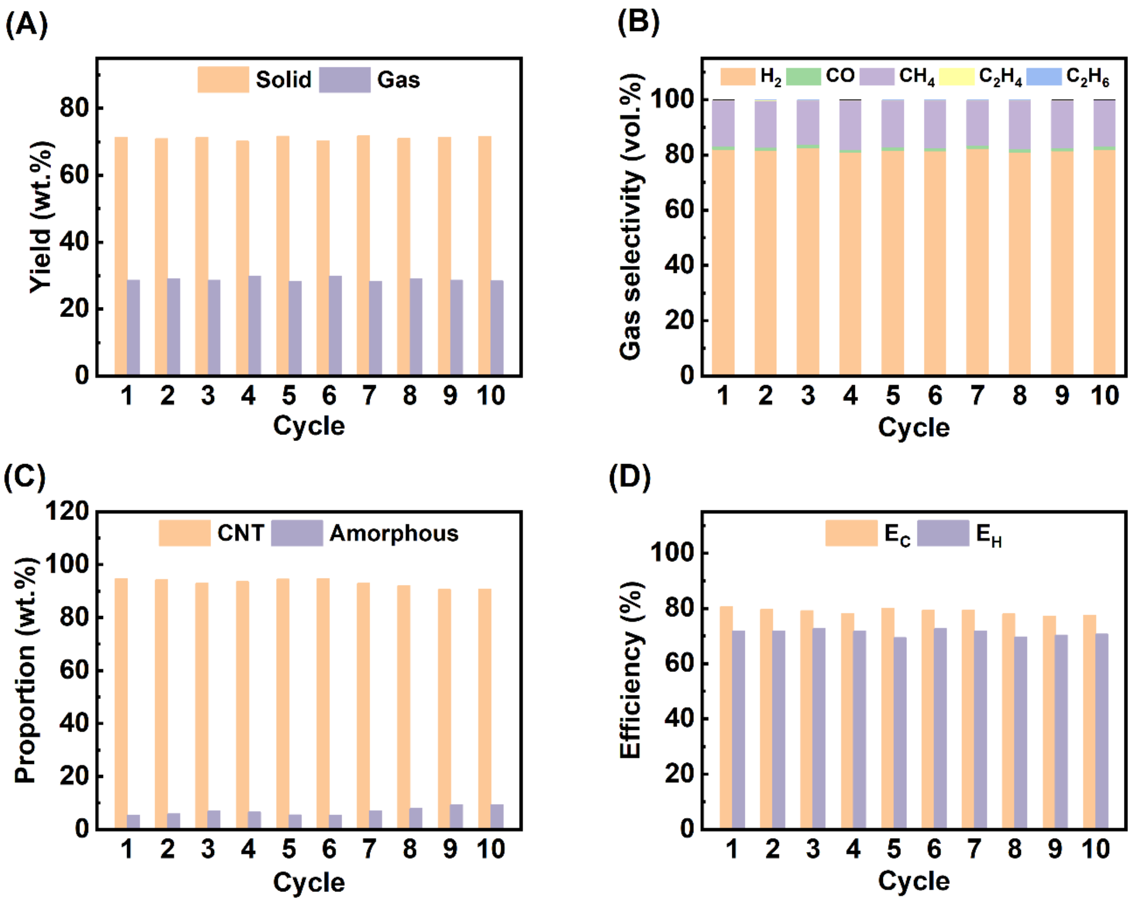

Figure S15. The effect of catalyst cycling performance on plastic pyrolysis-catalysis products.

(**A**) Solid and gas yields at different cycles. (**B**) Gas selectivity at different cycles. (**C**) CNT and amorphous carbon proportions of the produced carbon at different cycles. (**D**) E_C and E_H at different cycles.

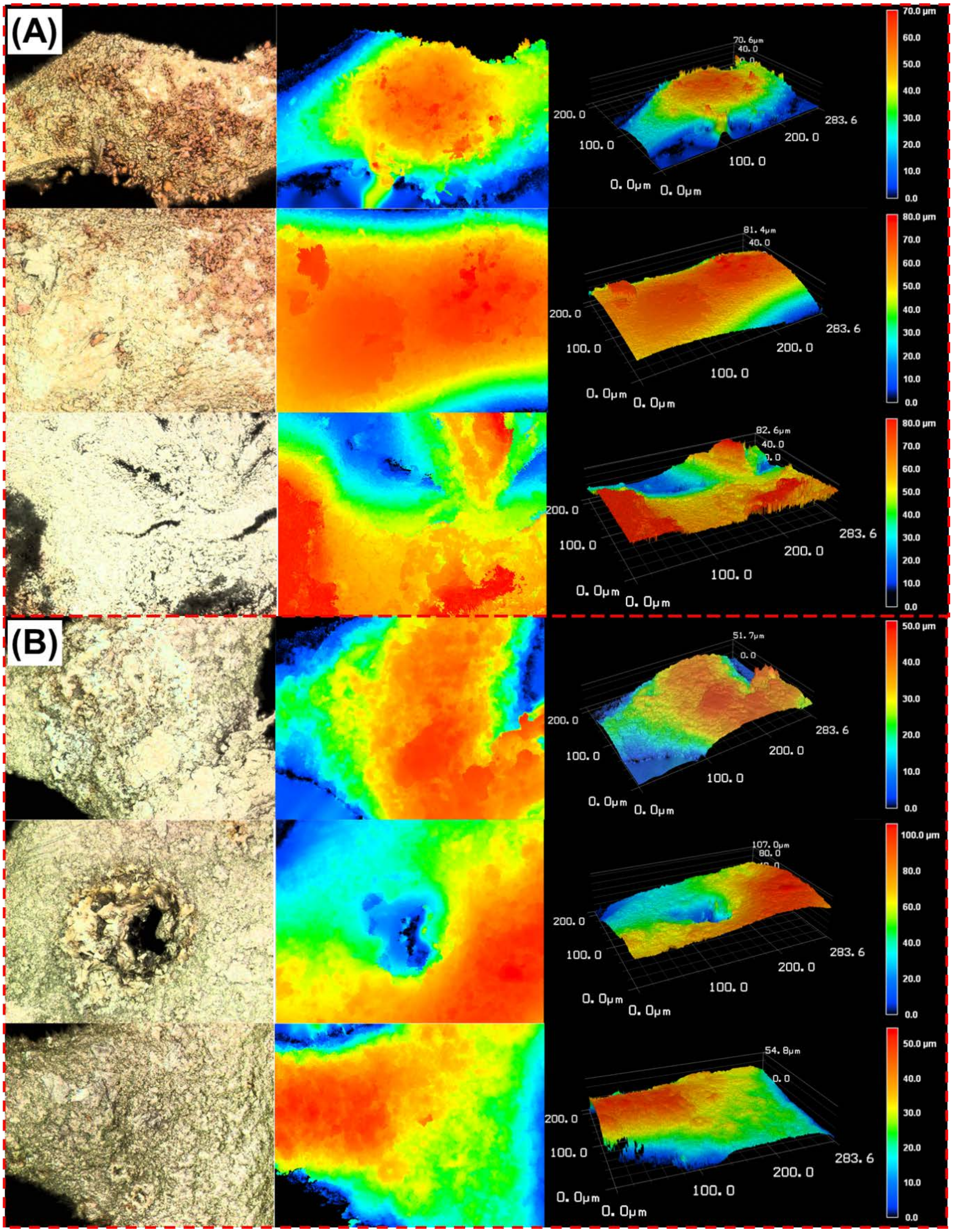

Figure S16. The surface roughness characterization.

Fe porous media (**A**) before and (**B**) after reaction.

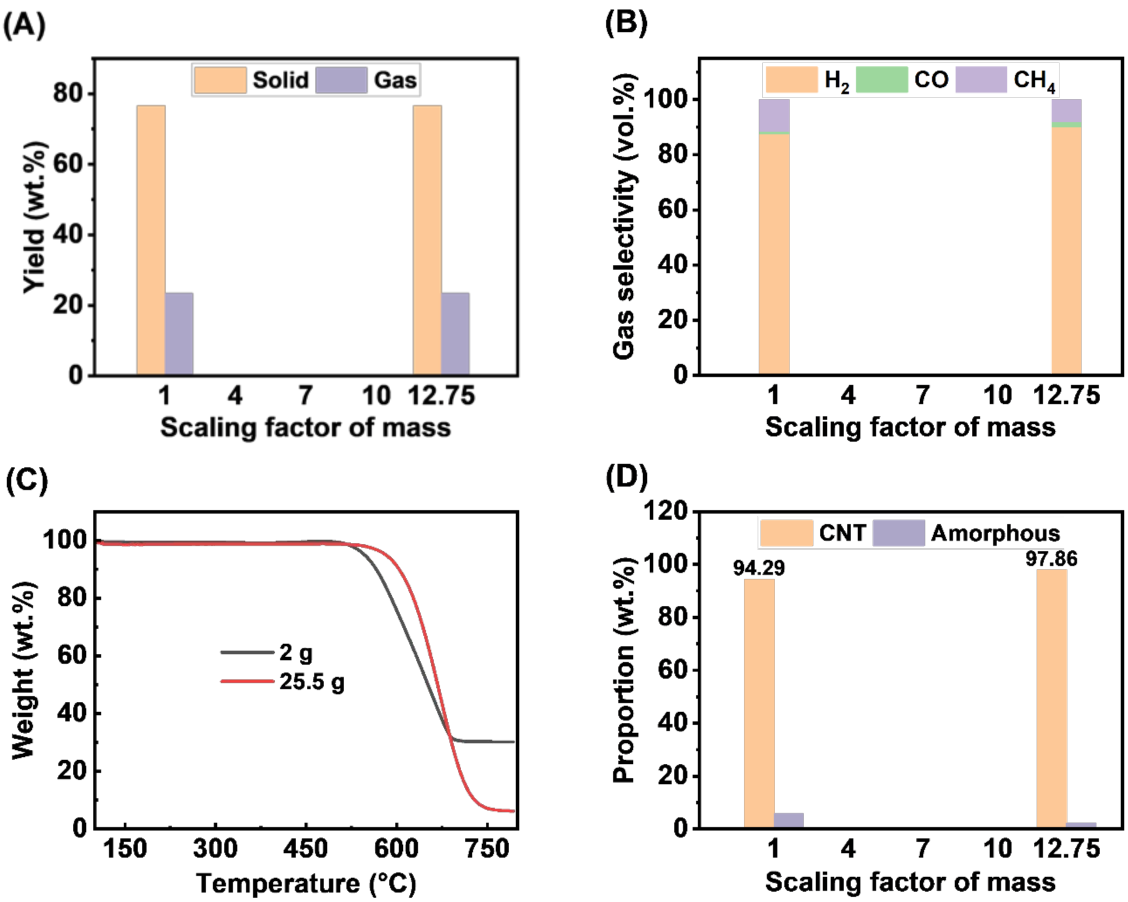

Figure S17. The effect of mass scaling factor on plastic pyrolysis-catalysis products.
(**A**) Solid and gas yields at different mass scaling factors. (**B**) Gas selectivity at different mass scaling factors. (**C**) TGA curves of the produced carbon at different mass scaling factors. (**D**) CNT and amorphous carbon proportions of the produced carbon at different mass scaling factors.

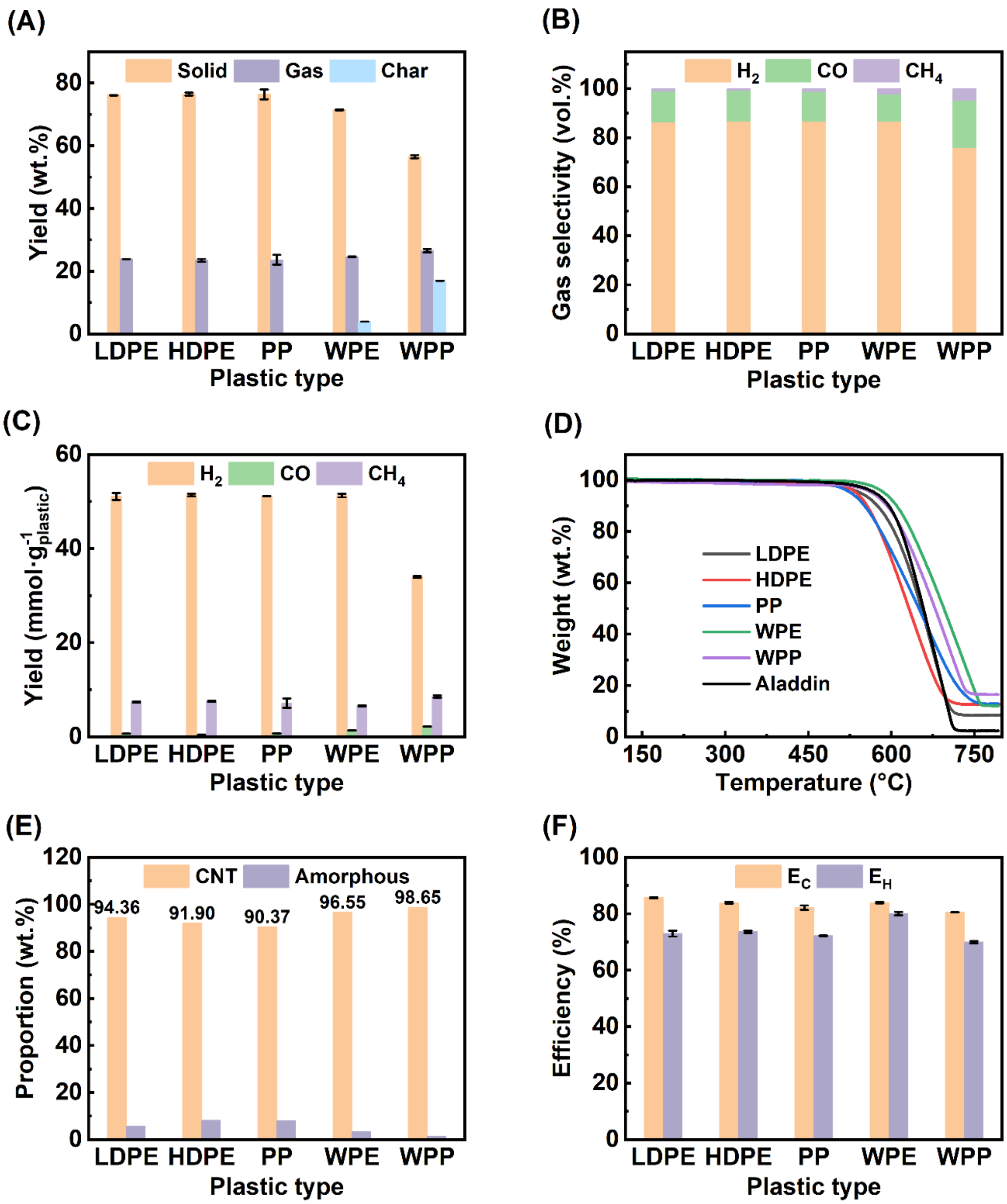

Figure S18. The effect of plastic type on plastic pyrolysis-catalysis products.
(**A**) Solid and gas yields at different plastic types. (**B**) Gas selectivity at different plastic types. (**C**) Specific gas yields at different plastic types. (**D**) TGA curves of the produced carbon at different plastic types. (**E**) CNT and amorphous carbon proportions of the produced carbon at different plastic types. (**F**) E_C and E_H at different plastic types. LDPE, HDPE, PP, WPE, and WPP represent low-density polyethylene, high-density polyethylene, polypropylene, waste polyethylene, and waste polypropylene.

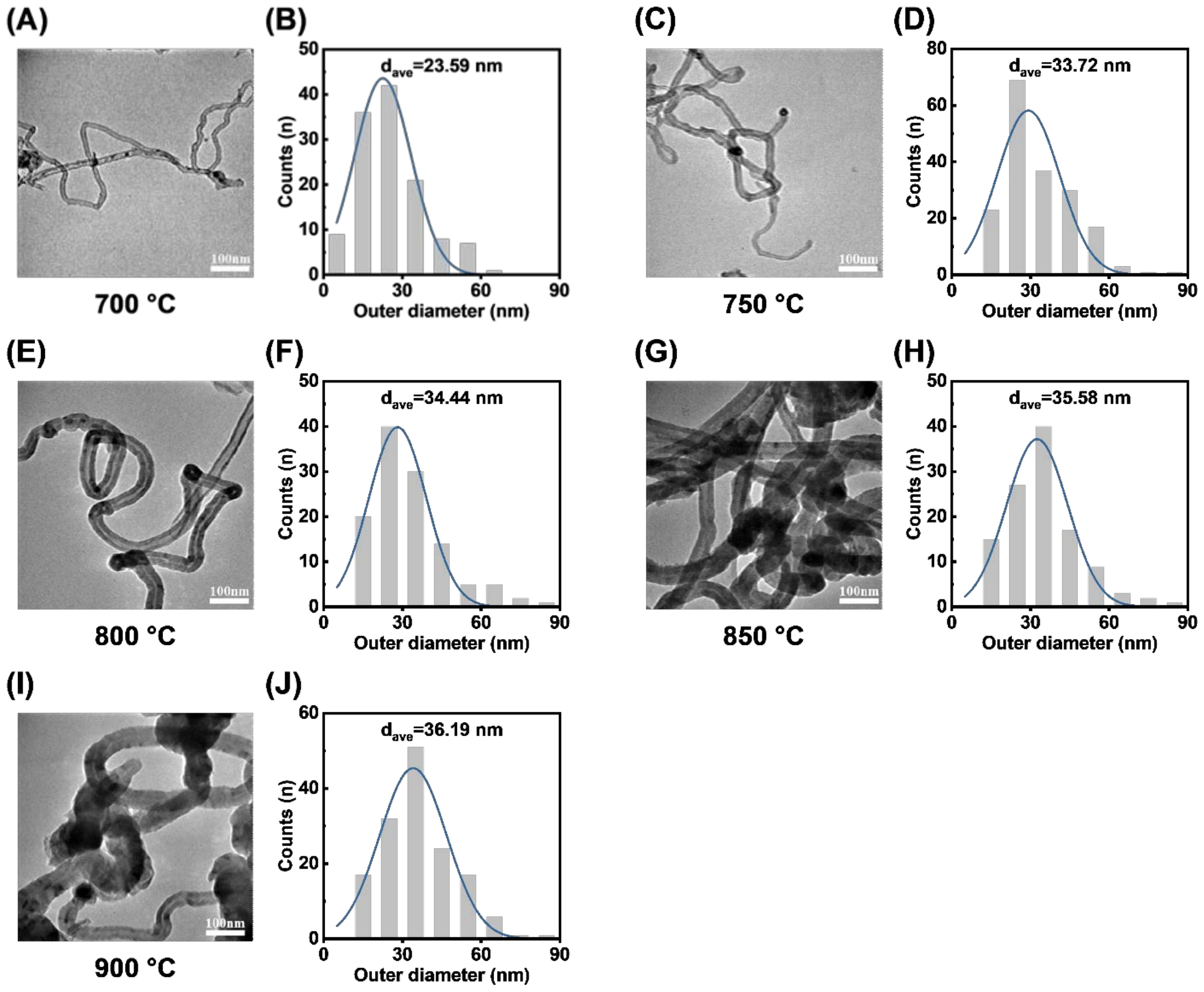

Figure S19. Effect of catalytic temperature on CNT produced using Fe porous catalyst of 10 PPI.

Transmission electron microscopy (TEM) image and outer diameter distribution of CNT produced at catalytic temperatures of (**A–B**) 700 °C, (**C–D**)750 °C, (**E–F**) 800 °C, (**G–H**) 850 °C, and (**I–J**) 900 °C.

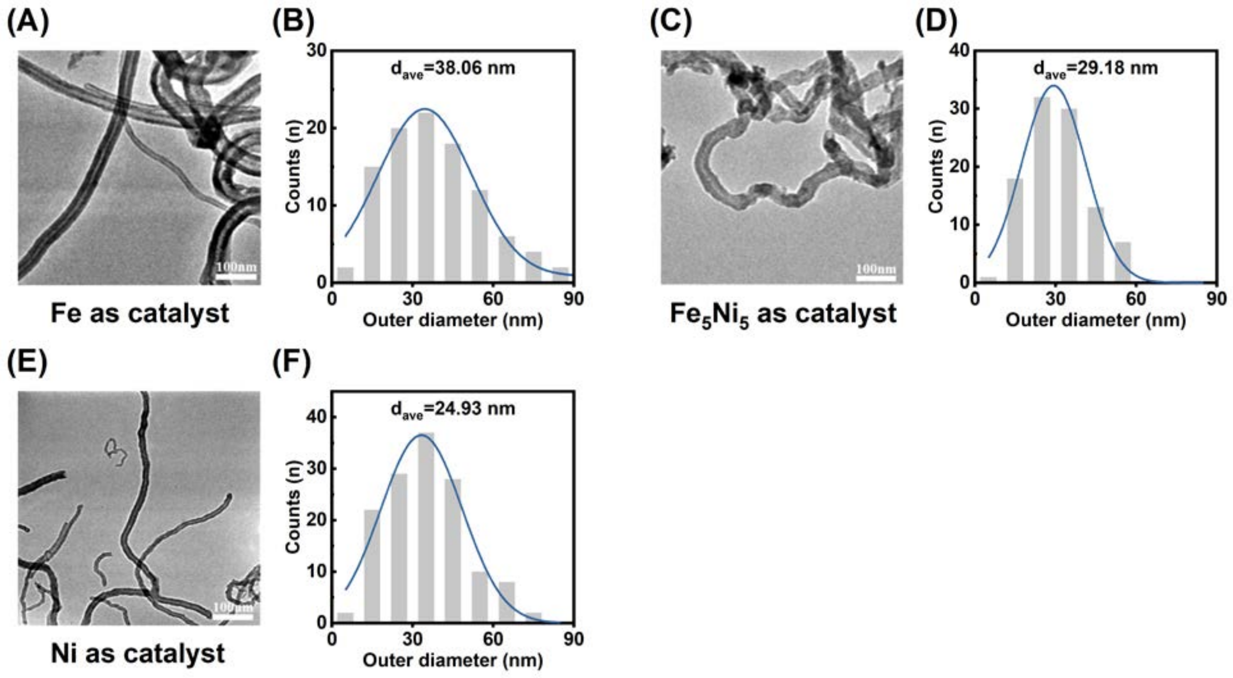

Figure S20. Effect of catalyst type on CNT produced using Fe porous catalyst of 30 PPI at catalytic temperature of 900 °C.

(**A–B**) Transmission electron microscopy (TEM) image and outer diameter distribution of CNT produced using Fe porous catalyst, (**C–D**) Fe_5Ni_5 porous catalyst, (**E–F**) Ni porous catalyst. (**G**) X-ray diffraction (XRD) pattern of CNT produced using Fe porous catalyst.

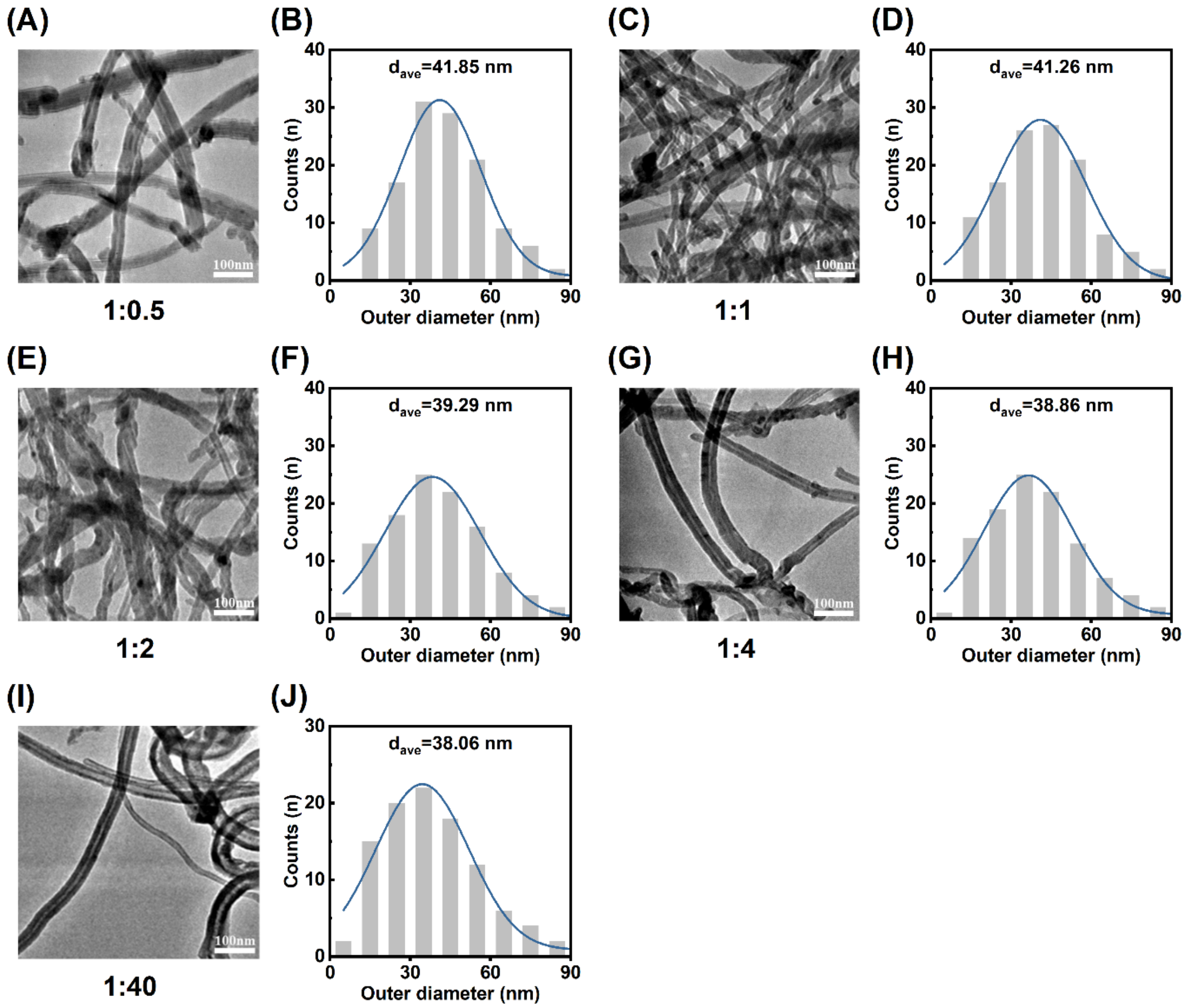

Figure S21. Effect of plastic-to-catalyst mass ratio on CNT produced using Fe porous catalyst of 30 PPI at catalytic temperature of 900 °C.

Transmission electron microscopy (TEM) image and outer diameter distribution of CNT produced at plastic-to-catalyst mass ratios of (**A–B**) 1:0.5, (**C–D**) 1:1, (**E–F**) 1:2, (**G–H**) 1:4, and (**I–J**) 1:40.

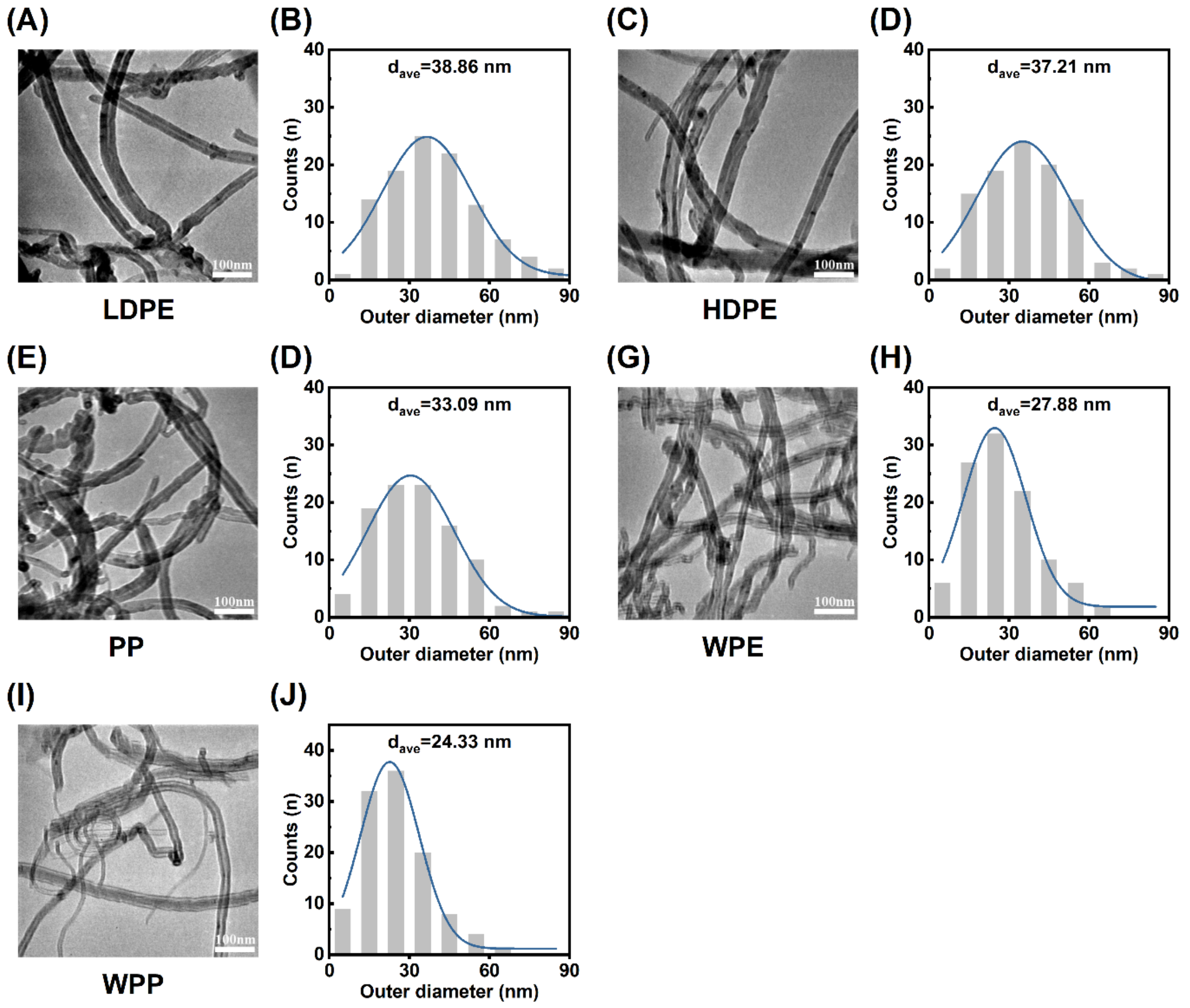

Figure S22. Effect of plastic type on CNT produced using Fe porous catalyst of 30 PPI at catalytic temperature of 900 °C.

TEM image and outer diameter distribution of CNT from (**A–B**) LDPE, (**C–D**) HDPE, (**E–F**) PP, (**G–H**) WPE, and (**I–J**) WPP.

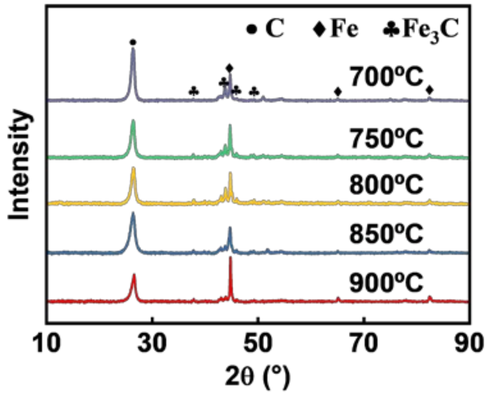

Figure S23. Effect of catalytic temperature on XRD pattern of CNT produced using Fe porous catalyst of 10 PPI.

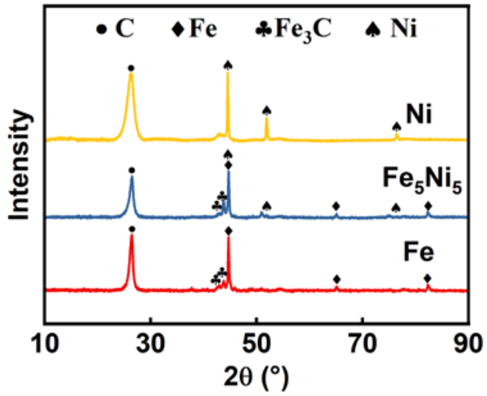

Figure S24. Effect of porous catalyst type on XRD pattern of CNT produced using at catalytic temperature of 900 °C.

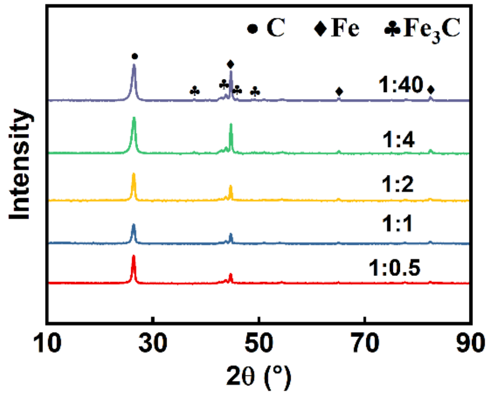

Figure S25. Effect of plastic-to-catalyst mass ratio on XRD pattern of CNT produced using at catalytic temperature of 900 °C.

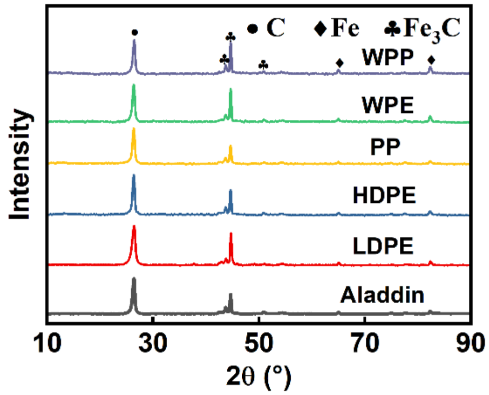

Figure S26. Effect of plastic type on XRD pattern of CNT produced using Fe porous catalyst of 30 PPI at catalytic temperature of 900 °C.

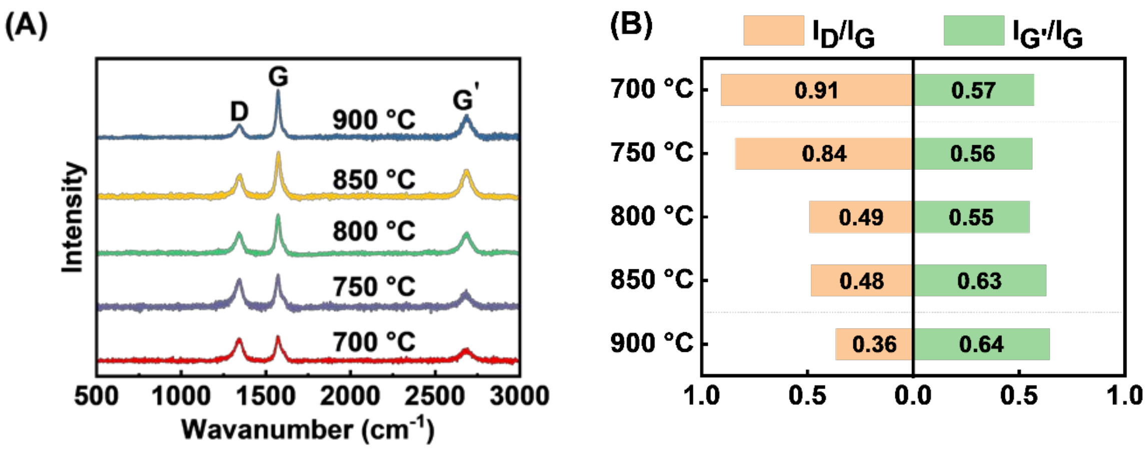

Figure S27. Raman spectra characterization of CNT produced at different catalytic temperatures.

(**A**) Raman spectra and (**B**) I_D/I_G and $I_{G'}/I_G$ ratios of CNT produced at different catalytic temperatures using Fe porous catalyst of 10 PPI.

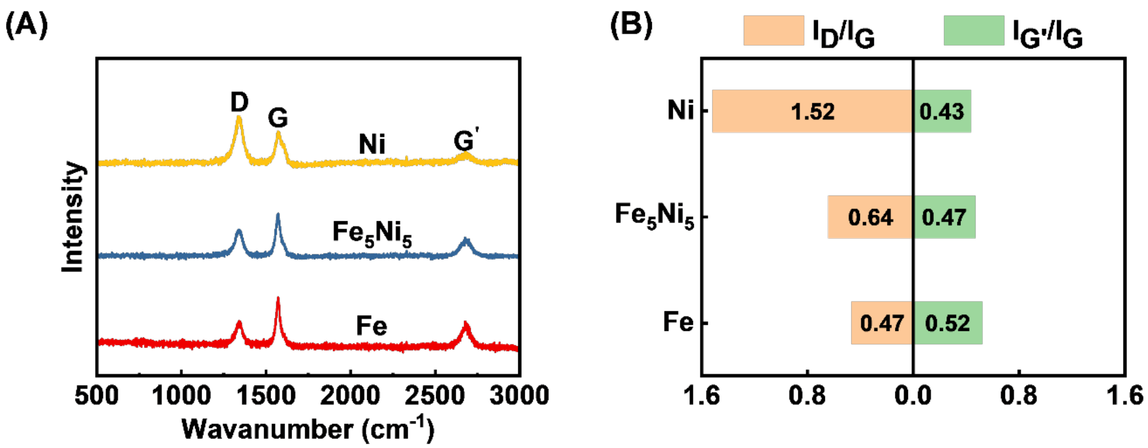

Figure S28. Raman spectra characterization of CNT produced using different metal porous catalysts.

(A) Raman spectra and (**B**) I_D/I_G and $I_{G'}/I_G$ ratios of CNT produced using different metal porous catalysts of 30 PPI at catalytic temperature of 900 °C.

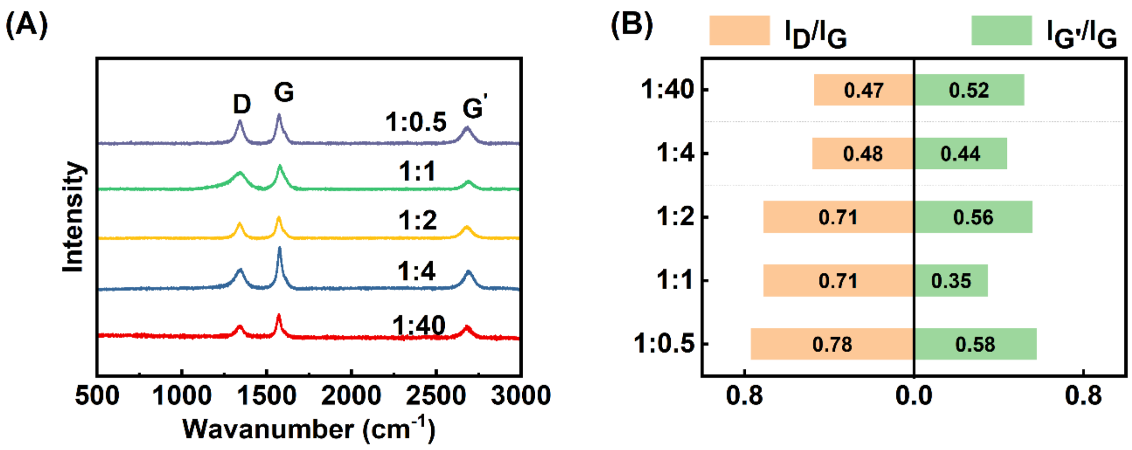

Figure S29. Raman spectra characterization of CNT produced from different plastic-to-catalyst mass ratios.

(**A**) Raman spectra and (**B**) I_D/I_G and $I_{G'}/I_G$ ratios of CNT produced from different plastic-to-catalyst mass ratios using Fe porous catalyst of 30 PPI at catalytic temperatures of 900 °C.

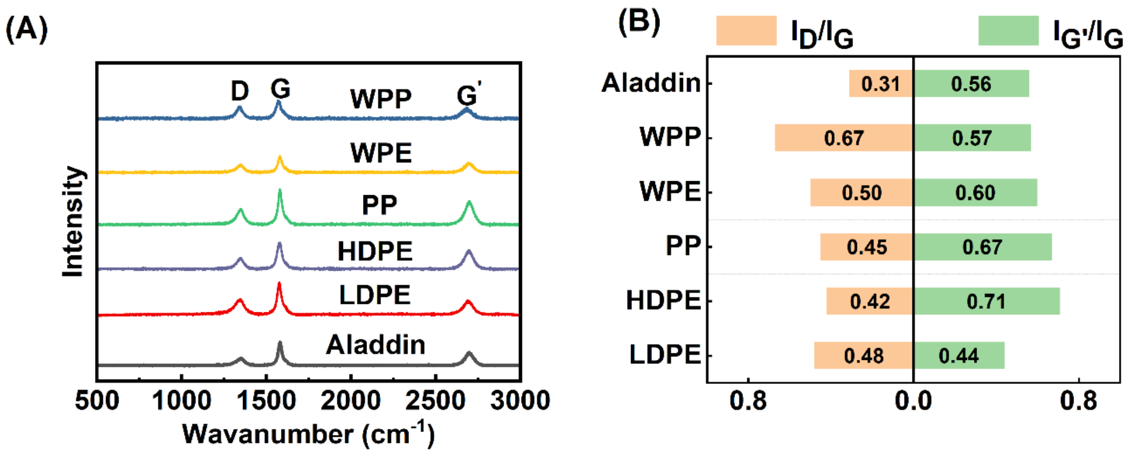

Figure S30. Raman spectra characterization of CNT produced from different plastics. (**A**) Raman spectra and (**B**) I_D/I_G and $I_{G'}/I_G$ ratios of CNT produced from different plastics using Fe porous catalyst of 30 PPI at catalytic temperatures of 900 °C.

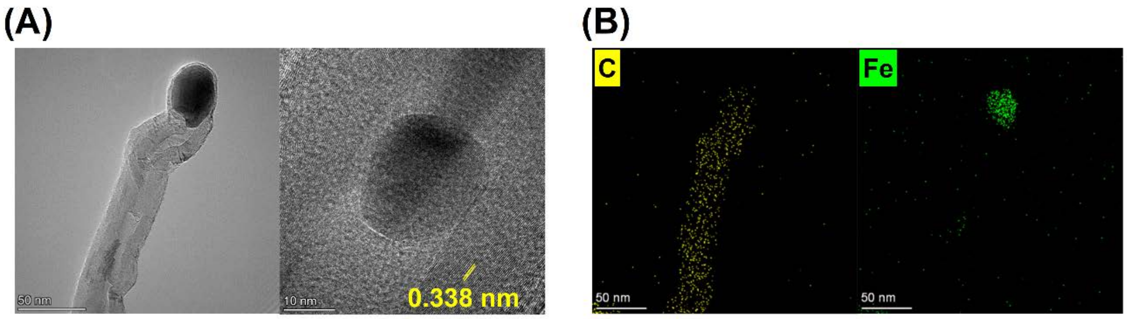

Figure S31. HRTEM coupled with HAADF characterization of CNT produced using Fe porous catalyst.

(**A**) HRTEM image of CNT produced using Fe porous catalyst. (**B**) Elemental distribution of CNT produced using Fe porous catalyst determined by HRTEM coupled with HAADF.

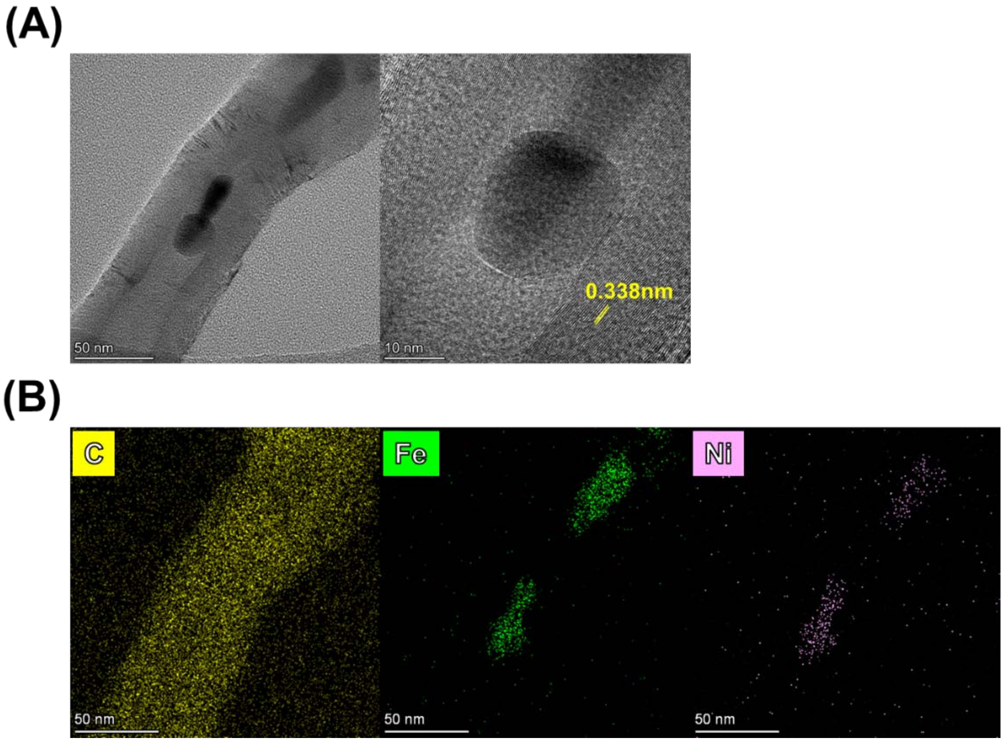

Figure S32. HRTEM coupled with HAADF characterization of CNT produced using Fe_5Ni_5 porous catalyst.

(**A**) HRTEM image of CNT produced using Fe_5Ni_5 porous catalyst. (**B**) Elemental distribution of CNT produced using Fe_5Ni_5 porous catalyst determined by HRTEM coupled with HAADF.

(A)

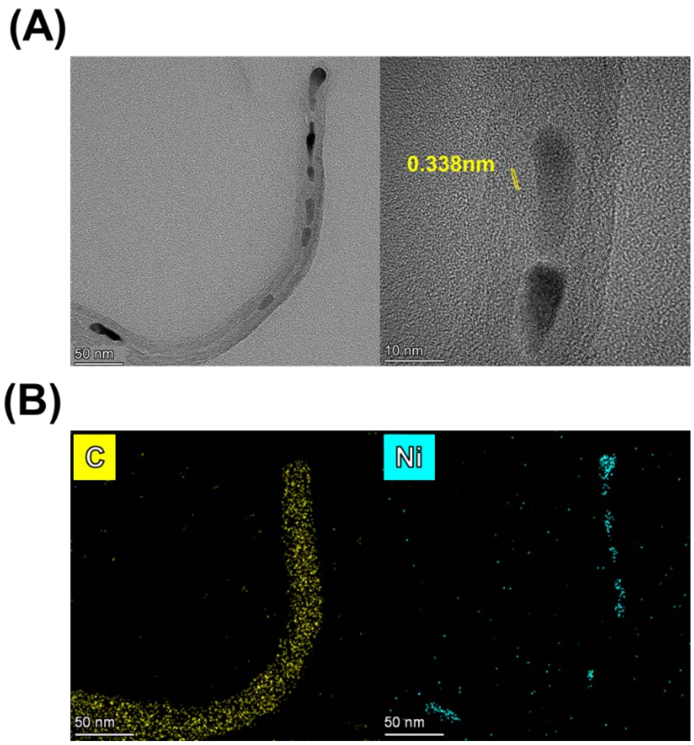

Figure S33. HRTEM coupled with HAADF characterization of CNT produced using Ni porous catalyst.

(**A**) HRTEM image of CNT produced using Ni porous catalyst. (**B**) Elemental distribution of CNT produced using Ni porous catalyst determined by HRTEM coupled with HAADF.

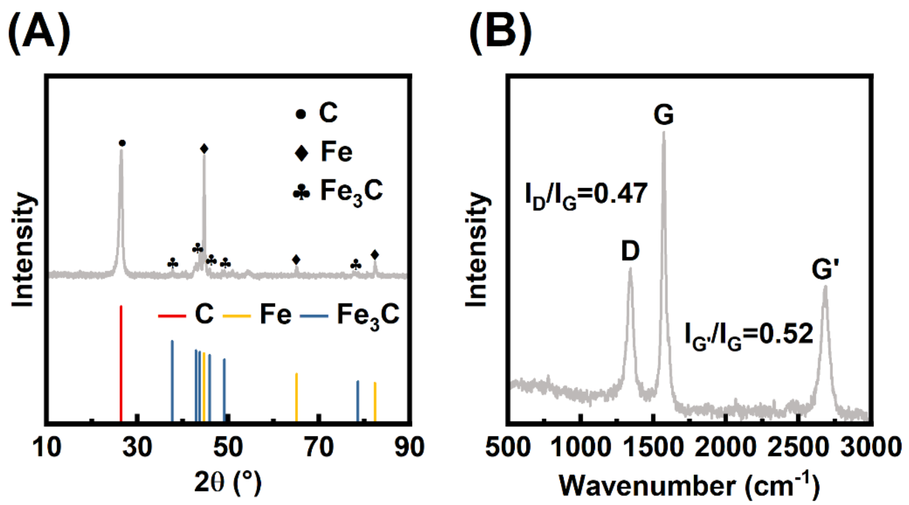

Figure S34. (A) X-ray diffraction (XRD) pattern of CNT produced using Fe porous catalyst. (B) Raman spectra of CNT produced using Fe porous catalyst.

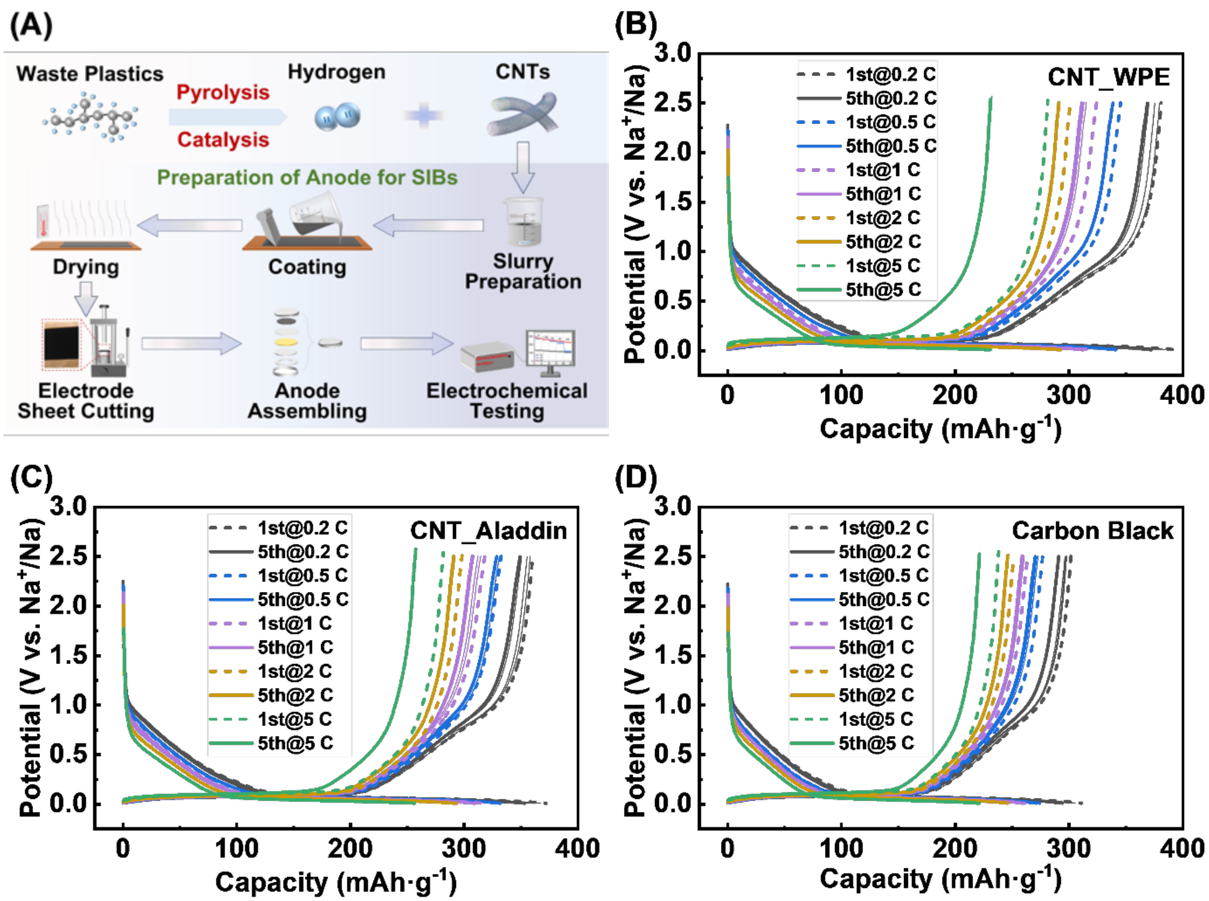

Figure S35. Performance test of different conductive materials in hard carbon anodes for SIBs.

(**A**) The schematic diagram of using CNT as conductive materials in anodes for SIBs. (**B**) Galvanostatic discharge-charge profiles of CNT_WPE as conductive materials at 0.2–5 C. (**C**) Galvanostatic discharge-charge profiles of CNT_Aladdin as conductive materials at 0.2–5 C. (**D**) Galvanostatic discharge-charge profiles of carbon black as conductive materials at 0.2–5 C. CNT_WPE and CNT_Aladdin represent CNT obtained from WPE and commercial products of Aladdin.

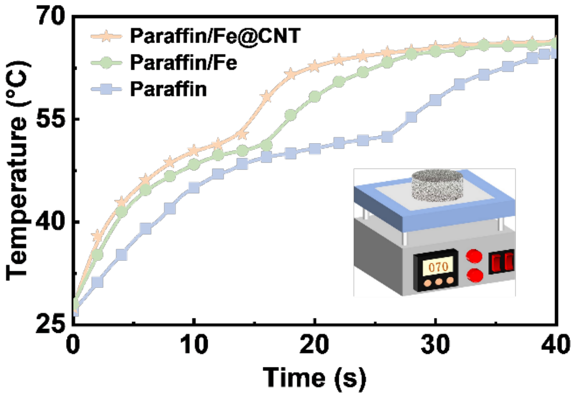

Figure S36. Temperature profiles of paraffin, paraffin/Fe, and paraffin/Fe@CNT under constant boundary temperature heating at 70 ^{o} C.

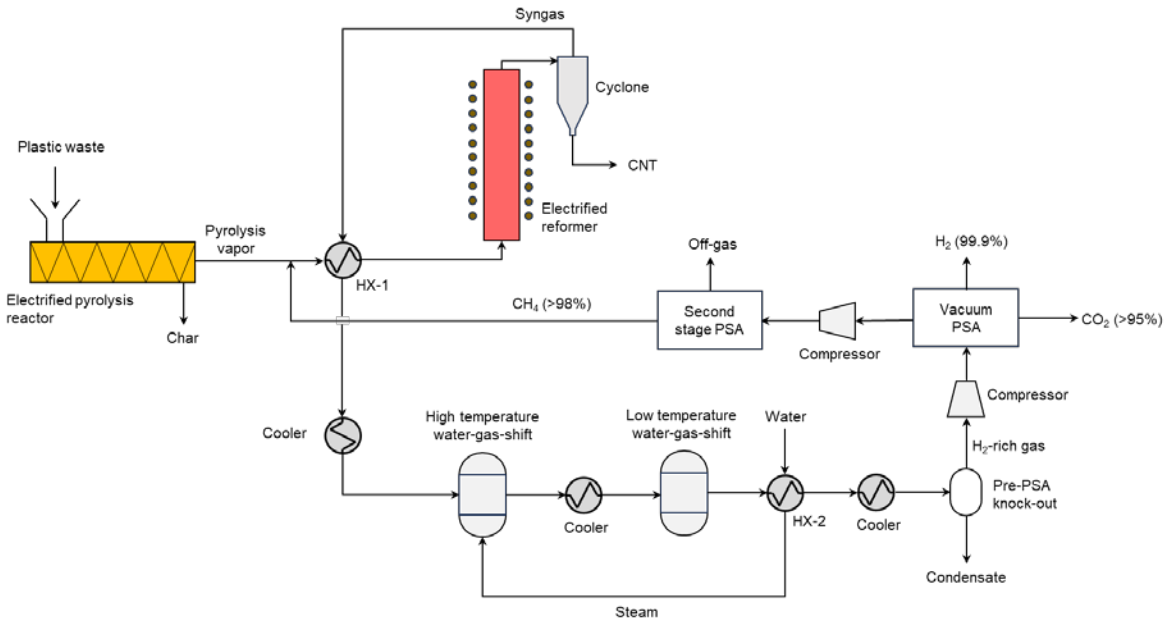

Figure S37. Schematic of the proposed CNT and H_2 co-production systems.

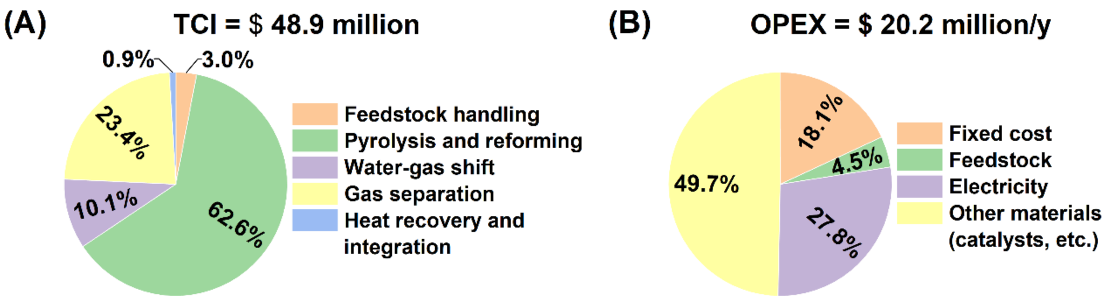

Figure S38. The total capital investment (TCI) and operating expenses (OPEX) of CNT and H_2 production using WPE feedstock for the base case scenario.

Calculated (**A**) TCI and (**B**) OPEX of CNT and H_2 production using WPE feedstock for the base case scenario.

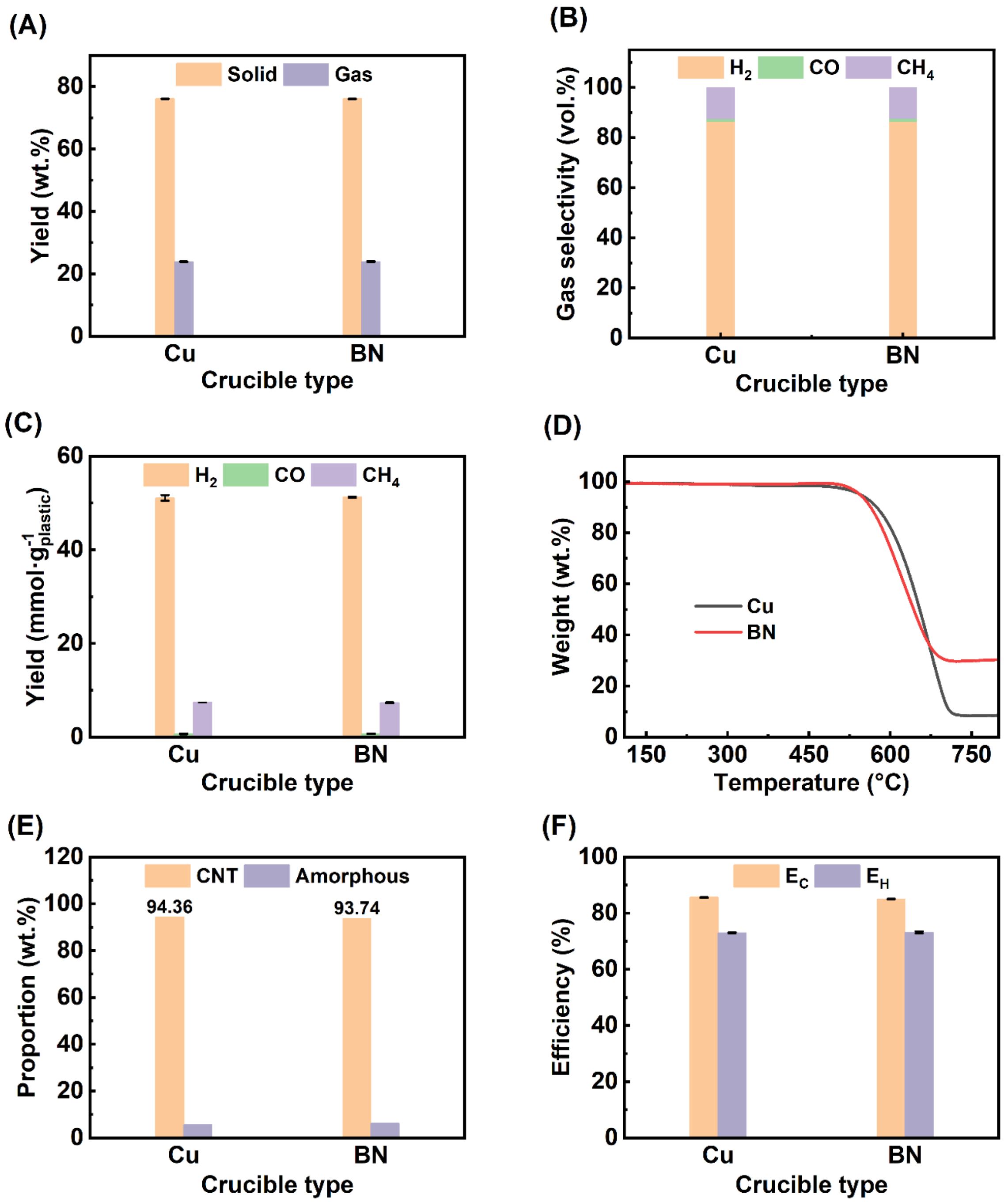

Figure S39. The effect of crucible types on plastic pyrolysis-catalysis products.

(A) Solid and gas yields at different crucible types. **(B)** Gas selectivity at different crucible types. **(C)** Specific gas yields at different crucible types. **(D)** TGA curves of the produced carbon at different crucible types. **(E)** CNT and amorphous carbon proportions of the produced carbon at different crucible types. **(F)** EC and EH at different crucible types.

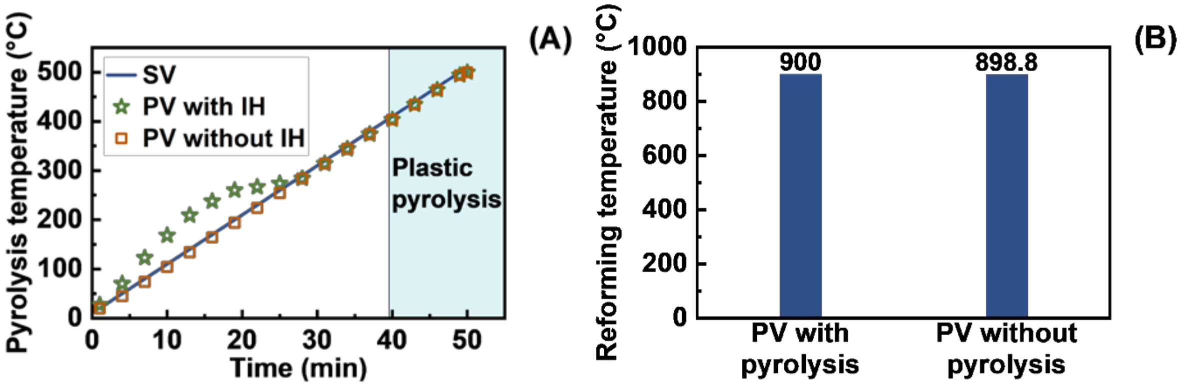

Figure S40. Pyrolysis and reforming temperature independence test.

(**A**) Pyrolysis temperature profiles with and without induction heating. (**B**) Reforming temperature values with and without pyrolysis heating. SV and PV represent the setting and practical temperature values. IH represents the induction heating.

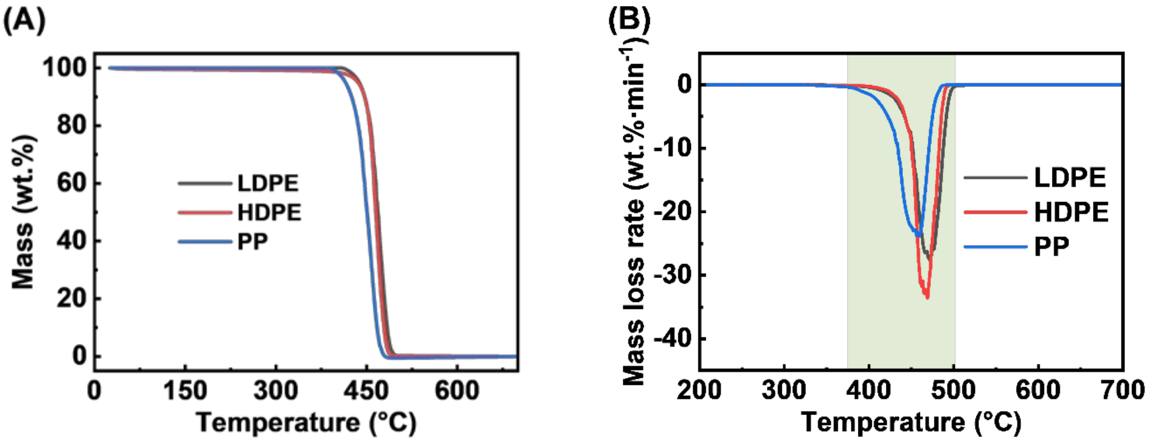

Figure S41. Thermogravimetric analysis of LDPE, HDPE, and PP at 10 K·min^{-1}.
(**A**) Mass and (**B**) mass loss rate.

Table S1.
Kinetic parameters of R1–R3.

Reaction number	*A* ($kg \cdot m^{-2} \cdot s^{-1}$)	*E* (kJ/mol)	*n*
R1	6.17×10^{-1}	29.0	0.75
R2	7.38×10^{-2}	17.2	1
R3	8.15×10^{-3}	17.0	1.37

Table S2.

E_C and E_H using the Fe porous media catalyst with electromagnetic induction monolith heating and values in previous studies.

Plastic type	E_C(%)	E_H(%)	Plastic-to-catalyst ratio	Catalyst	Code	Ref.
LDPE	67.92	61.69	1:0.5	Fe metal foam	TW	This work
LDPE	84.81	72.96	1:4	Fe metal foam	TW	This work
LDPE	67.60	64.00	1:15	SS316	S1	[46]
LDPE	30.40	26.80	1:0.5	10%Fe/Al_2O_3	S2	[47]
LDPE	32.00	31.50	1:0.5	10%Fe/Al_2O_3	S3	[48]
HDPE	66.02	60.94	1:0.5	Fe metal foam	TW	This work
HDPE	83.66	73.55	1:4	Fe metal foam	TW	This work
HDPE	72.80	64.70	1:15	SS316	S1	[46]
HDPE	33.80	33.60	1:0.5	10%Fe/Al_2O_3	S3	[48]
HDPE	72.33	48.07	1:1	20%Fe/Al_2O_3	S4	[49]
PP	67.58	61.03	1:0.5	Fe metal foam	TW	This work
PP	83.03	72.15	1:4	Fe metal foam	TW	This work
PP	79.80	74.90	1:15	SS316	S1	[46]
PP	43.00	36.70	1:0.4	FeNi=3:1	S5	[50]
PP	23.10	-	1:0.5	10%Ni/cordierite	S6	[51]
WPE	66.04	66.98	1:0.5	Fe metal foam	TW	This work
WPE	85.74	80.05	1:4	Fe metal foam	TW	This work
WPE	52.97	66.22	1:1	20%Fe/Al_2O_3	S4	[49]
WPE	29.98	55.97	1:1	Ni-Fe/ZSM5	S7	[52]
WPE	38.03	78.82	1:0.5	Ni–Mn–Al	S8	[53]
WPP	62.70	62.26	1:0.5	Fe metal foam	TW	This work
WPP	78.86	69.95	1:4	Fe metal foam	TW	This work
WPP	63.98	63.73	1:1	20%Fe/Al_2O_3	S4	[49]
WPP	27.42	49.14	1:0.5	$(Mn,Fe)_2O_3$	S9	[54]
WPP	33.83	35.84	1:0.4	Fe/SiO_2	S10	[55]

Table S3.

E_C and E_H using the Fe porous media catalyst with electromagnetic induction monolith heating and values in previous studies.

Plastic type	I_D/I_G ratio	CNT proportion (wt.%)	Reference
WPE	0.512	96.55	This work
WPE	0.890	92.31	[49]
WPE	1.280	92.75	[53]
WPP	0.671	98.65	This work
WPP	0.730	95.23	[54]
WPP	-	95.75	[55]

Table S4.

Resultant activation energies E_a for the intermediate steps involved in CH_4 cracking on the Fe, Ni, and Fe_5Ni_5 surfaces in this study and references [27–29].

Reaction Path	E_a in this study		
	Ni	Fe	Fe_5Ni_5
**$CH_4 \rightarrow CH_3^* + H^*$**	0.85	1.02	1.65
**$CH_3^* + H^* \rightarrow CH_2^* + H_2$**	0.7	1.3	1.4
**$CH_2^* + 2H^* \rightarrow CH^* + 3H^*$**	0.33	0.4	0.55
**$CH^* + 3H^* \rightarrow C + 2H_2$**	1.02	1.36	1.5
Reaction Path	E_a in the references [27–29]		
	Ni [27]	Fe [28–29]	Fe_5Ni_5 [28]
**$CH_4 \rightarrow CH_3^* + H^*$**	0.91–1.31	1.02–1.25	0.89–1.25
**$CH_3^* + H^* \rightarrow CH_2^* + H_2$**	0.68–0.89	0.58–0.61	0.65–0.71
**$CH_2^* + 2H^* \rightarrow CH^* + 3H^*$**	0.30–0.41	0.13	0.32–0.36
**$CH^* + 3H^* \rightarrow C + 2H_2$**	1.36–1.40	1.04–1.06	1.11–1.16

Table S5.
Metal content of ash derived from WPE and WPP.

Ash metal content (wt.%)	WPE	WPP
Na	2.20	0.50
Mg	1.66	1.56
Al	11.47	8.53
K	0.13	0.80
Ca	20.25	27.99
Fe	0.55	0.86
Si	60.93	57.64
Zn	0.08	0.08

Table S6.

Textural properties of Fe porous skeleton before and after reaction.

Property	Before reaction	After reaction
BET surface area ($m^2 \cdot g^{-1}$)	4.376	1.215
External surface area ($m^2 \cdot g^{-1}$)	4.376	1.215
Micropore area ($m^2 \cdot g^{-1}$)	0	0
Micropore volume (MPV) ($cm^3 \cdot g^{-1}$)	0	0
Total pore volume (TPV) ($cm^3 \cdot g^{-1}$)	3.336×10^{-3}	8.239×10^{-3}
Average pore size (nm)	3.077	27.420

Table S7.

Graphitization parameters of CNT at different catalytic temperatures using Fe porous catalyst of 10 PPI.

Temperature	$2\theta^a$ (°)	$D_C{}^b$ (nm)	$d_{(002)}{}^c$ (nm)	g^d	n^e
700 °C	26.28	14.13	0.338	0.60	41.80
750 °C	26.33	14.22	0.338	0.67	42.07
800 °C	26.34	14.47	0.338	0.69	42.81
850 °C	26.35	14.96	0.338	0.70	44.26
900 °C	26.39	17.22	0.338	0.72	50.95

[a] 2θ represents the angle between the incoming and outgoing beam directions.
[b] D_C represents the average crystallite size.
[c] $d_{(002)}$ represents the lattice distance.
[d] g represents the graphitization degree.
[e] n represents the average number of graphene layers.

Table S8.

Graphitization parameters of CNT using different metal porous catalysts of 30 PPI at catalytic temperature of 900 °C.

Catalyst	2θ (°)	D_C (nm)	$d_{(002)}$ (nm)	g	n
Fe	26.34	14.81	0.338	0.69	43.82
Fe_5Ni_5	26.32	11.70	0.338	0.65	34.62
Ni	26.26	10.64	0.339	0.57	31.39

Table S9.

Graphitization parameters of CNT produced from different plastic-to-catalyst mass ratios using Fe porous catalyst of 30 PPI at catalytic temperature of 900 °C.

Plastic-to-catalyst mass ratio	2θ (°)	D_C (nm)	$d_{(002)}$ (nm)	g	n
1:0.5	26.28	16.13	0.339	0.60	47.60
1:1	26.31	15.79	0.338	0.64	46.64
1:2	26.33	14.96	0.338	0.67	44.25
1:4	26.34	14.82	0.338	0.69	43.84
1:40	26.34	14.81	0.338	0.69	43.80

Table S10.

Graphitization parameters of CNT produced from different plastics using Fe porous catalyst of 30 PPI at catalytic temperature of 900 °C and commercial CNT.

Plastic type	2θ (°)	D_C (nm)	$d_{(002)}$ (nm)	g	n
LDPE	26.34	14.82	0.338	0.69	43.84
HDPE	26.34	14.43	0.338	0.69	42.68
PP	26.33	14.31	0.338	0.67	42.31
WPE	26.31	14.24	0.338	0.64	42.06
WPP	26.31	14.09	0.338	0.64	41.64
Commercial CNT	26.38	18.53	0.338	0.75	54.89

Table S11.

Comparison of the performance of hard carbon@CNT with reported carbon materials.

Carbon type	Current density	Capacity ($mAh\cdot g^{-1}$)	Code	Ref.	Note
Hard carbon@CNT	2C	288.6	TW	This work	Average value of 5 cycles
Expanded carbon	2C	280.0	R1	[56]	/
Hard carbon	<2C (600 mA·g-1)	187.3	R2	[57]	Average value of 10 cycles
Hard carbon	<2C (500 mA·g-1)	122.9	R3	[58]	/
Hard carbon	2C	85.8	R4	[59]	Average value of 4 cycles
Hard carbon@CNT	5C	254.7	TW	This work	Average value of 5 cycles
Expanded carbon	5C	248.7	R1	[56]	/
Hard carbon	5C	153.8	R5	[460]	Calculated in [R1]
Hard carbon	<5C (1200 mA·g-1)	138.4	R2	[57]	Average value of 10 cycles
Hard carbon	5C	108.7	R6	[61]	Calculated in [R1]

Table S12.

Pyrolysis product composition and yield used in the process model.

Yields (wt.% of sample)	Samples	
	WPE	WPP
**Solid char**	3.95	17.00
**Permanent gases**		
H_2	0.10	0.002
CO	0.02	0.05
CO_2	2.61	6.70
CH_4	2.70	2.54
C_2H_4	4.16	4.38
C_2H_6	0.22	0.23
C_3H_6	2.92	3.94
C_3H_8	0.53	0.79
C_4H_8	1.69	1.29
C_4H_{10}	0.28	0.21
**Condensable oil**		
n-heptane	1.08	
1-octene	1.51	
heptane, 4-methyl-		0.82
1-nonene	1.73	
2,6-dimethyl-1-heptene		9.08
1-decene	1.70	
p-menthane		2.77
1-undecene	2.04	
cycloundecane		0.82
1-dodecene	2.21	
2,4-dimethyl-3-decene		3.75
1-tridecene	3.12	
1,1,3,5-tetramethylhexahyroindan		1.42
1-tetradecene	2.88	
1-pentadecene	3.10	12.05
1-hexadecene	3.60	2.90
1-heptadecene	3.79	
1-octadecen	4.25	
3-octadecene,-(E)-		4.09
1-nonadecene	4.11	
7-octadecyne		2.27
1-eicosene	4.07	
1-heneicosen	4.28	6.33
1-docosene	4.45	2.18
1-tricosene	3.93	
n-tetracosane	3.92	

1-tetracosene		1.67
n-pentacosane	3.79	
$C_{25}H_{48}$-N4		2.40
1-hexacosene	3.53	
n-heptacosane	3.08	
1-heptacosene		2.26
n-octacosane	3.17	2.51
n-nonacosane	2.62	0.36
n-triacontane	2.50	0.96
n-hentriacontane	1.76	1.75
n-dotriacontane	1.46	0.81
n-tritriacontane	1.24	0.37
n-tetratriacontane	0.81	1.31
n-pentatriacontane	0.58	
n-hexatriacontane	0.47	

Table S13.
Input data for capital cost estimation.

System	Base cost	Base scale	Scale unit	Scaling Factor	Ref.
**Equipment cost**					
Feedstock handling					
Storage	1 M$2001	33.5	tonne/h	1.00	[62]
Feeding	0.41 M$2001	33.5	tonne/h	0.41	[62]
Pyrolysis unit					
Reactor	0.018 M$1989	1	tonne/h	0.60	[43]
Reformer unit					
Furnace	28746 €2005	18.91	kg natural gas/h	0.7	[63]
Inductive heating element	1.27 M$2014	1	MW_{el}	1	[64]
Cyclone			Gas flow rate (1000 cfm)		[43]
Water-gas shift	9.54 M€2007	1246	MW-LHV input	0.67	
HT Heat Exchanger (total installed)			A in m^2	Cost chart	[43]
Gas separation					
Compressor	3300 €2006	5	kW_{el}	0.82	[65]
VPSA unit	27.95 M€2013	73.91	Nm^3/s	0.67	[66]
PSA unit	6.25 M€2002	0.294	kmol/s-purge gas	0.74	[67]
**Direct Plant Cost**	Cost estimation (% of TPEC)				[68]
Installation	39				
Instrumentation	13				
Piping	31				
Electrical installation	10				
Buildings	29				
Yard improvements	10				
Service facilities	55				
Land	6				
Direct Plant Cost (DPC)	293% of TPEC	DPC = Total Installed cost (TIC)			
**Indirect capital cost (IDC)**					
Engineering and	32				

supervision	
Construction expenses	34
Contractor's fee	18
Contingency + Additional 20%	36 + 20% FCI
Fixed capital Investment (FCI)	DPC + IDC = 413% of TPEC + 0.83
Working capital (WC)	74
**Total Capital Investment (TCI)**	FCI + WC = 487% of TPEC + 0.83

Table S14.
Summary of financial assumptions for economic analysis [33].

Parameter	Assumption
Currency	USD 2024
Operating time or uptime	8000/h per year
Plant financing	100% equity financing
Internal rate of return	8%
Plant life	25 years
Income tax rate	21%
Working capital	15% of fixed capital investment
Depreciation schedule	10-year straight line
Construction period	2.5 years (8% Y1, 60% Y2, 32% Y3)
Plant salvage value	No value
Start up time	6 months (Revenue = 50% normal, Variable costs = 75% normal; Fixed costs = 100% normal)
Government support	No financial support

Table S15.
Input data for operating cost estimation.

Specification	Cost estimation		Ref.	
Fixed operating cost				
Labor	2.5% TCI		[69]	
Maintanance	2.5% TCI		[70]	
Insurance and local taxes	1% TCI		[70]	
Plant overhead	50% operating labor		[70]	
Misc. OPEX	10% operating labor		[70]	
Variable operating cost				
Items	Value	Unit	Ref.	Notes
WPE and WPP feedstock	112.7	\$/t	[71]	Prices of both WPE and WPP are the same.
Reforming catalyst	4.46<br>1255.3<br>1021.8	$\$/kg_{catalyst}$<br>$\$/t_{WPE}$<br>$\$/t_{WPP}$		Estimated based on the catalyst consumption during the lab-scale test, including the cost of iron powder (0.939 \$/kg [72]), polyurethane foam (0.767 \$/kg-catalyst [73]), sintering process (0.181 \$/kg-catalyst [74]), dipping process (0.075 \$/kg-catalyst [75]), overhead costs (2.5 \$/kg-catalyst, estimated based on lab-scale process). The Fe porous catalyst needs to be replaced when the skeleton remaining mass fraction is below 20 wt.%.
WGS catalyst	14	k€/m^3	[68,75]	
Disposal of pyrolysis char	0.26	\$/kg	[38]	A typical cost for the treatment and disposal of solid hazardous waste in China .
Electricity	0.104	\$/kWh	[76]	Price of electricity in China.
Cooling water	0.03	€/m^3	[43]	

Supplementary References

[1] Pan, R., Martins, M.F. and Debenest, G., 2022. Optimization of oil production through ex-situ catalytic pyrolysis of waste polyethylene with activated carbon. Energy, 248, p.123514.

[2] Pan, R., Martins, M.F. and Debenest, G., 2023. Interactions of operating parameters on the production of waste polypropylene pyrolysis oil: neural fuzzy model and genetic algorithm optimization. Journal of Material Cycles and Waste Management, 25(1), pp.198-210.

[3] Yao, D., Zhang, Y., Williams, P.T., Yang, H. and Chen, H., 2018. Co-production of hydrogen and carbon nanotubes from real-world waste plastics: Influence of catalyst composition and operational parameters. Applied Catalysis B: Environmental, 221, pp.584-597.

[4] Zareidarmiyan, A., Parisio, F., Makhnenko, R.Y., Salarirad, H. and Vilarrasa, V., 2021. How equivalent are equivalent porous media? Geophysical Research Letters, 48(9), p.e2020GL089163.

[5] Kuhwald, C., Türkhan, S. and Kirschning, A., 2022. Inductive heating and flow chemistry–a perfect synergy of emerging enabling technologies. Beilstein Journal of Organic Chemistry, 18(1), pp.688-706.

[6] Elsaady, W., Moughton, C. and Nasser, A., 2023. Coupled numerical modelling and experimental analysis of domestic induction heating systems. Applied Thermal Engineering, 227, p.120170.

[7] Wu, Z., Caliot, C., Bai, F., Flamant, G., Wang, Z., Zhang, J. and Tian, C., 2010. Experimental and numerical studies of the pressure drop in ceramic foams for volumetric solar receiver applications. Applied Energy, 87(2), pp.504-513.

[8] Dietrich, B., Schabel, W., Kind, M. and Martin, H., 2009. Pressure drop measurements of ceramic sponges—determining the hydraulic diameter. Chemical Engineering Science, 64(16), pp.3633-3640.

[9] Vafai, K. ed., 2015. Handbook of porous media. Crc Press.

[10] Zhang, H., Shuai, Y., Lougou, B.G., Jiang, B., Wang, F., Cheng, Z. and Tan, H., 2020. Effects of multilayer porous ceramics on thermochemical energy conversion and storage efficiency in solar dry reforming of methane reactor. Applied Energy, 265, p.114799.

[11] Fuqiang, W., Lanxin, M., Ziming, C., Jianyu, T., Xing, H. and Linhua, L., 2017. Radiative heat transfer in solar thermochemical particle reactor: a comprehensive review. Renewable and sustainable energy reviews, 73, pp.935-949.

[12] Pan, R., Martins, M.F. and Debenest, G., 2021. Pyrolysis of waste polyethylene in a semi-batch reactor to produce liquid fuel: Optimization of operating conditions. Energy Conversion and Management, 237, p.114114.

[13] Philippe, R., Serp, P., Kalck, P., Kihn, Y., Bordère, S., Plee, D., Gaillard, P., Bernard, D. and Caussat, B., 2009. Kinetic study of carbon nanotubes synthesis by fluidized bed chemical vapor deposition. AIChE journal, 55(2), pp.450-464.

[14] Endo, H., Kuwana, K., Saito, K., Qian, D., Andrews, R. and Grulke, E.A., 2004. CFD prediction of carbon nanotube production rate in a CVD reactor. Chemical Physics Letters, 387(4-6), pp.307-311.

[15] Kuwana, K., Endo, H., Saito, K., Qian, D., Andrews, R. and Grulke, E.A., 2005. Catalyst deactivation in CVD synthesis of carbon nanotubes. Carbon, 43(2), pp.253-260.

[16] Inayat, A., Freund, H., Zeiser, T. and Schwieger, W., 2011. Determining the specific surface area of ceramic foams: The tetrakaidecahedra model revisited. Chemical Engineering Science, 66(6), pp.1179-1188.

[17] Ranjan, P., Kannan, P., Al Shoaibi, A. and Srinivasakannan, C., 2012. Modeling of ethane thermal cracking kinetics in a pyrocracker. Chemical engineering & technology, 35(6), pp.1093-1097.
[18] Kuvshinov, G.G., Mogilnykh, Y.I. and Kuvshinov, D.G., 1998. Kinetics of carbon formation from CH4–H2 mixtures over a nickel containing catalyst. Catalysis today, 42(3), pp.357-360.
[19] Zavarukhin, S.G. and Kuvshinov, G.G., 2004. The kinetic model of formation of nanofibrous carbon from CH4–H2 mixture over a high-loaded nickel catalyst with consideration for the catalyst deactivation. Applied Catalysis A: General, 272(1-2), pp.219-227.
[20] Argaman, N. and Makov, G., 2000. Density functional theory: An introduction. American Journal of Physics, 68(1), pp.69-79.
[21] Perdew, J.P., Burke, K. and Ernzerhof, M., 1996. Generalized gradient approximation made simple. Physical Review Letters, 77(18), p.3865.
[22] Luo, L., Wang, M., Cui, Y., Chen, Z., Wu, J., Cao, Y., Luo, J., Dai, Y., Li, W.X., Bao, J. and Zeng, J., 2020. Surface iron species in palladium–iron intermetallic nanocrystals that promote and stabilize CO2 methanation. Angewandte Chemie, 132(34), pp.14542-14550.
[23] Qiu, M., Tao, H., Li, Y., Li, Y., Ding, K., Huang, X., Chen, W. and Zhang, Y., 2018. Toward improving CO2 dissociation and conversion to methanol via CO-hydrogenation on Cu (100) surface by introducing embedded Co nanoclusters as promoters: A DFT study. Applied Surface Science, 427, pp.837-847.
[24] Grimme, S., Ehrlich, S. and Goerigk, L., 2011. Effect of the damping function in dispersion corrected density functional theory. Journal of Computational Chemistry, 32(7), pp.1456-1465.
[25] Bhunia, K., Chandra, M., Sharma, S.K., Pradhan, D. and Kim, S.J., 2023. A critical review on transition metal phosphide based catalyst for electrochemical hydrogen evolution reaction: Gibbs free energy, composition, stability, and true identity of active site. Coordination Chemistry Reviews, 478, p.214956.
[26] Henkelman, G., Uberuaga, B.P. and Jónsson, H., 2000. A climbing image nudged elastic band method for finding saddle points and minimum energy paths. The Journal of Chemical Physics, 113(22), pp.9901-9904.
[27] Li, J., Croiset, E. and Ricardez-Sandoval, L., 2012. Methane dissociation on Ni (1 0 0), Ni (1 1 1), and Ni (5 5 3): a comparative density functional theory study. Journal of Molecular Catalysis A: Chemical, 365, pp.103-114.
[28] Liu, H., Wang, B., Fan, M., Henson, N., Zhang, Y., Towler, B.F. and Harris, H.G., 2013. Study on carbon deposition associated with catalytic CH4 reforming by using density functional theory. Fuel, 113, pp.712-718.
[29] Fan, C., Zhou, X.G., Chen, D., Cheng, H.Y. and Zhu, Y.A., 2011. Toward CH4 dissociation and C diffusion during Ni/Fe-catalyzed carbon nanofiber growth: a density functional theory study. The Journal of Chemical Physics, 134(13).
[30] Liu, H., Zhang, R., Yan, R., Wang, B. and Xie, K., 2011. CH4 dissociation on NiCo (1 1 1) surface: A first-principles study. Applied surface science, 257(21), pp.8955-8964.
[31] Zhu, Y.A., Chen, D., Zhou, X.G. and Yuan, W.K., 2009. DFT studies of dry reforming of methane on Ni catalyst. Catalysis Today, 148(3-4), pp.260-267.
[32] An, W., Zeng, X.C. and Turner, C.H., 2009. First-principles study of methane dehydrogenation on a bimetallic Cu/Ni (111) surface. The Journal of Chemical Physics, 131(17).

[33] Yang, H., Nuran Zaini, I., Pan, R., Jin, Y., Wang, Y., Li, L., Caballero, J.J.B., Shi, Z., Subasi, Y., Nurdiawati, A. and Wang, S., 2024. Distributed electrified heating for efficient hydrogen production. Nature Communications, 15(1), p.3868.
[34] Shokrollahi, M., Teymouri, N., Ashrafi, O., Navarri, P. and Khojasteh-Salkuyeh, Y., 2024. Methane pyrolysis as a potential game changer for hydrogen economy: Techno-economic assessment and GHG emissions. International Journal of Hydrogen Energy, 66, pp.337-353.
[35] Razmi, A.R., Hanifi, A.R. and Shahbakhti, M., 2024. Techno-economic analysis of a novel concept for the combination of methane pyrolysis in molten salt with heliostat solar field. Energy, 301(C).
[36] Pangestu, M.R.G. and Zahid, U., 2024. Techno-economic analysis of integrating methane pyrolysis and reforming technology for low-carbon ammonia. Energy Conversion and Management, 322, p.119125.
[37] Channiwala, S.A. and Parikh, P.P., 2002. A unified correlation for estimating HHV of solid, liquid and gaseous fuels. Fuel, 81(8), pp.1051-1063.
[38] Zhang, J., Zhang, Y., Leong, Z.H., Zhang, Y., Chen, T., Fei, F. and Wen, Z., 2025. Mapping the Recycling Potential of Bottom Ashes from Waste-to-Energy Plants toward Circular Economy: Evidence from China. Environmental Science & Technology, 59(38), pp.20805-20816.
[39] Almind, M.R., Vendelbo, S.B., Hansen, M.F., Vinum, M.G., Frandsen, C., Mortensen, P.M. and Engbæk, J.S., 2020. Improving performance of induction-heated steam methane reforming. Catalysis Today, 342, pp.13-20.
[40] Chianese, S., Loipersböck, J., Malits, M., Rauch, R., Hofbauer, H., Molino, A. and Musmarra, D., 2015. Hydrogen from the high temperature water gas shift reaction with an industrial Fe/Cr catalyst using biomass gasification tar rich synthesis gas. Fuel Processing Technology, 132, pp.39-48.
[41] Streb, A., Hefti, M., Gazzani, M. and Mazzotti, M., 2019. Novel adsorption process for co-production of hydrogen and CO2 from a multicomponent stream. Industrial & Engineering Chemistry Research, 58(37), pp.17489-17506.
[42] Augelletti, R., Conti, M. and Annesini, M.C., 2017. Pressure swing adsorption for biogas upgrading. A new process configuration for the separation of biomethane and carbon dioxide. Journal of Cleaner Production, 140, pp.1390-1398.
[43] Garrett, D.E., 2012. Chemical engineering economics. Springer Science & Business Media.
[44] Peters, M.S., Timmerhaus, K.D. and West, R.E., 2003. Plant design and economics for chemical engineers (Vol. 4). New York: McGraw-hill.
[45] Gerdes, K., Summers, W.M. and Wimer, J., 2011. Cost Estimation Methodology for NETL Assessments of Power Plant Performance DOE/NETL-2011/1455. no. September, p.26.
[46] Liu, Q., Jiang, D., Zhou, H., Yuan, X., Wu, C., Hu, C., Luque, R., Wang, S., Chu, S., Xiao, R. and Zhang, H., 2023. Pyrolysis–catalysis upcycling of waste plastic using a multilayer stainless-steel catalyst toward a circular economy. Proceedings of the National Academy of Sciences, 120(39), p.e2305078120.
[47] Acomb, J.C., Wu, C. and Williams, P.T., 2016. The use of different metal catalysts for the simultaneous production of carbon nanotubes and hydrogen from pyrolysis of plastic feedstocks. Applied Catalysis B: Environmental, 180, pp.497-510.
[48] Cai, N., Li, X., Xia, S., Sun, L., Hu, J., Bartocci, P., Fantozzi, F., Williams, P.T., Yang, H. and Chen, H., 2021. Pyrolysis-catalysis of different waste plastics over Fe/Al2O3 catalyst:

High-value hydrogen, liquid fuels, carbon nanotubes and possible reaction mechanisms. Energy Conversion and Management, 229, p.113794.

[49] Jie, X., Li, W., Slocombe, D., Gao, Y., Banerjee, I., Gonzalez-Cortes, S., Yao, B., AlMegren, H., Alshihri, S., Dilworth, J. and Thomas, J., 2020. Microwave-initiated catalytic deconstruction of plastic waste into hydrogen and high-value carbons. Nature Catalysis, 3(11), pp.902-912.

[50] Yao, D. and Wang, C.H., 2020. Pyrolysis and in-line catalytic decomposition of polypropylene to carbon nanomaterials and hydrogen over Fe-and Ni-based catalysts. Applied Energy, 265, p.114819.

[51] Wang, J., Shen, B., Lan, M., Kang, D. and Wu, C., 2020. Carbon nanotubes (CNT) production from catalytic pyrolysis of waste plastics: The influence of catalyst and reaction pressure. Catalysis Today, 351, pp.50-57.

[52] Zhang, X., Jiang, Y., Kong, G., Liu, Q., Zhang, G., Wang, K., Cao, T., Cheng, Q., Zhang, Z., Ji, G. and Han, L., 2023. CO2-mediated catalytic upcycling of plastic waste for H2-rich syngas and carbon nanomaterials. Journal of Hazardous Materials, 460, p.132500.

[53] Wu, C., Nahil, M.A., Miskolczi, N., Huang, J. and Williams, P.T., 2014. Processing real-world waste plastics by pyrolysis-reforming for hydrogen and high-value carbon nanotubes. Environmental Science & Technology, 48(1), pp.819-826.

[54] He, S., Li, C., Sun, H., Parlett, C.M., Qiao, Y., Wang, Y., Xu, Y., Gao, N. and Wu, C., 2024. Promotion of manganese on Fe-based catalyst for the production of carbon nanotubes (CNT) from plastics. Chemical Engineering Journal, 492, p.152306.

[55] Liu, X., Zhang, Y., Nahil, M.A., Williams, P.T. and Wu, C., 2017. Development of Ni-and Fe-based catalysts with different metal particle sizes for the production of carbon nanotubes and hydrogen from thermo-chemical conversion of waste plastics. Journal of Analytical and Applied Pyrolysis, 125, pp.32-39.

[56] Cheng, Z., Zhang, H., Cui, J., Zhao, J., Dai, S., Zhang, Z., Song, K., Wang, S., Yuan, Y., Chen, Q. and Kong, X., 2025. Interlayer-expanded carbon anodes with exceptional rates and long-term cycling via kinetically decoupled carbonization. Joule.

[57] Lu, P., Sun, Y., Xiang, H., Liang, X. and Yu, Y., 2018. 3D amorphous carbon with controlled porous and disordered structures as a high‐rate anode material for sodium‐ion batteries. Advanced Energy Materials, 8(8), p.1702434.

[58] He, X.X., Zhao, J.H., Lai, W.H., Li, R., Yang, Z., Xu, C.M., Dai, Y., Gao, Y., Liu, X.H., Li, L. and Xu, G., 2021. Soft-carbon-coated, free-standing, low-defect, hard-carbon anode to achieve a 94% initial coulombic efficiency for sodium-ion batteries. ACS Applied Materials & Interfaces, 13(37), pp.44358-44368.

[59] Gao, X., Sun, Y., He, B., Nuli, Y., Wang, J. and Yang, J., 2024. A bifuctional presodiation reagent for hard carbon anodes enhancing performance of sodium-ion batteries. ACS Energy Letters, 9(3), pp.1141-1147.

[60] Chen, D., Zhang, W., Luo, K., Song, Y., Zhong, Y., Liu, Y., Wang, G., Zhong, B., Wu, Z. and Guo, X., 2021. Hard carbon for sodium storage: mechanism and optimization strategies toward commercialization. Energy & Environmental Science, 14(4), pp.2244-2262.

[61] Aslam, M.K., Niu, Y., Hussain, T., Tabassum, H., Tang, W., Xu, M. and Ahuja, R., 2021. How to avoid dendrite formation in metal batteries: Innovative strategies for dendrite suppression. Nano Energy, 86, p.106142.

[62] Hamelinck, C.N. and Faaij, A.P., 2002. Future prospects for production of methanol and hydrogen from biomass. Journal of Power sources, 111(1), pp.1-22.

[63] Feedstock, E.W., 2006. Equipment design and cost estimation for small modular biomass systems, synthesis gas cleanup, and oxygen separation equipment; Task 1: Cost estimates of small modular systems. Nature Renew Energy Laboratory.
[64] Bera, A. and Babadagli, T., 2015. Status of electromagnetic heating for enhanced heavy oil/bitumen recovery and future prospects: A review. Applied energy, 151, pp.206-226.
[65] Marcoberardino, G.D., Vitali, D., Spinelli, F., Binotti, M. and Manzolini, G., 2018. Green hydrogen production from raw biogas: A techno-economic investigation of conventional processes using pressure swing adsorption unit. Processes, 6(3), p.19.
[66] Cordiano, B., 2019. Investigation of scale-up and techno-economic potential of VPSA technology for single cycle H2 and CO2 purification (Doctoral dissertation, Politecnico di Torino).
[67] Kreutz, T., Williams, R., Consonni, S. and Chiesa, P., 2005. Co-production of hydrogen, electricity and CO2 from coal with commercially ready technology. Part B: Economic analysis. International Journal of Hydrogen Energy, 30(7), pp.769-784.
[68] Spallina, V., Pandolfo, D., Battistella, A., Romano, M.C., Annaland, M.V.S. and Gallucci, F., 2016. Techno-economic assessment of membrane assisted fluidized bed reactors for pure H2 production with CO2 capture. Energy conversion and management, 120, pp.257-273.
[69] Yang, H., Nurdiawati, A., Gond, R., Chen, S., Wang, S., Tang, B., Jin, Y., Zaini, I.N., Shi, Z., Wang, W. and Martin, A., 2024. Carbon-negative valorization of biomass waste into affordable green hydrogen and battery anodes. International journal of hydrogen energy, 49, pp.459-471.
[70] Caballero, J.J.B., Zaini, I.N., Nurdiawati, A., Fedorova, I., Cao, P., Lewin, T., Jönsson, P.G. and Yang, W., 2025. Electrified catalytic steam reforming for renewable syngas production: Experimental demonstration, process development and techno-economic analysis. Applied Energy, 377, p.124556.
[71] Zhoushan Jinke Renewable Resources Co., China. n.d.
[72] Shanghai Metals Markets. n.d. https://www.metal.com/Lithium%20Battery%20Cathode%20Precursor%20and%20Material/202405170002
[73] Pingxiang Bestn Chemical Packing Co., Ltd. n.d. https://bestnpacking.en.made-in-china.com/product/hyYxrHGMgVWc/China-20ppi-30ppi-40ppi-Open-Cell-Bio-Filter-Foam-Reticulated-Polyurethane-Filter-Sponge-for-Aquarium.html?pv_id=1iaflst439a6&faw_id=1iaflta3c827
[74] Azevedo, J.M., CabreraSerrenho, A. and Allwood, J.M., 2018. Energy and material efficiency of steel powder metallurgy. Powder Technology, 328, pp.329-336.
[75] Samal, P. and Newkirk, J. eds., 2015. Powder Metallurgy. ASM International.
[76] China Economic Information Center. n.d. https://www.ceicdata.com/en/china/price-monitoring-center-ndrc-transaction-price-production-material-electricity/cn-usage-price-electricity-for-industry-35-kv--above-harbin
